# Synthesis, structure, and reactions of a copper–sulfido cluster comprised of the parent Cu_2_S unit: {(NHC)Cu}_2_(μ-S)†
†Electronic supplementary information (ESI) available. CCDC 1421010–1421012. For ESI and crystallographic data in CIF or other electronic format see DOI: 10.1039/c5sc03258j



**DOI:** 10.1039/c5sc03258j

**Published:** 2015-10-20

**Authors:** Junjie Zhai, Alexander S. Filatov, Gregory L. Hillhouse, Michael D. Hopkins

**Affiliations:** a Department of Chemistry , The University of Chicago , 929 East 57th Street , Chicago , Illinois 60637 , USA . Email: junjie@uchicago.edu

## Abstract

The synthesis of the first CuI2(μ-S) complex, {(IPr*)Cu}_2_(μ-S) (IPr* = 1,3-bis(2,6-(diphenylmethyl)-4-methylphenyl)imidazol-2-ylidene; **1**), has been accomplished *via* three synthetic routes: (1) salt metathesis between (IPr*)CuCl and Na_2_S; (2) silyl-deprotection reaction between (IPr*)Cu(SSiMe_3_) and (IPr*)CuF; and (3) acid–base reaction between (IPr*)Cu(SH) and (IPr*)Cu(O^*t*^Bu). The X-ray crystal structure of **1** exhibits two two-coordinate copper centers connected by a bent Cu–S–Cu linkage. Application of these synthetic routes to analogous precursors containing the sterically smaller ligand IPr (1,3-bis(2,6-di-isopropylphenyl)imidazol-2-ylidene), in place of IPr*, resulted in the formation of a transient product proposed as {(IPr)Cu}_2_(μ-S) (**2**), which decomposes quickly in solution. The instability of **2** probably results from the insufficient steric protection provided by IPr ligands to the unsaturated Cu_2_(μ-S) core; in contrast, **1** is stable both in solution and solid state for weeks. The nucleophilic sulfido ligand in **1** reacts with haloalkyl electrophiles (benzyl halides and dibromoalkanes) with formation of C–S bonds, affording (IPr*)Cu(SCH_2_Ph) and cyclic thioethers, respectively.

## Abstract 2

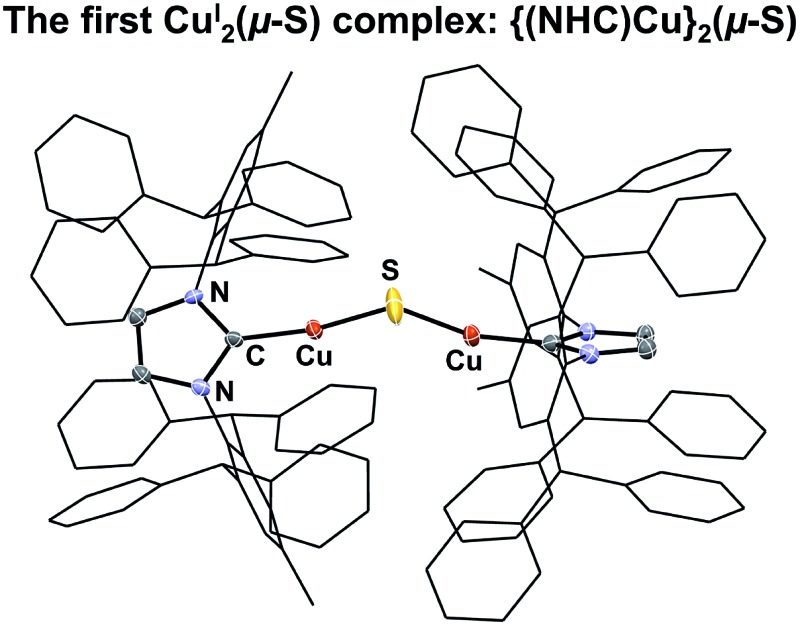
The first CuI2(μ-S) complex, {(IPr*)Cu}_2_(μ-S) (IPr* = 1,3-bis(2,6-(diphenylmethyl)-4-methylphenyl)imidazol-2-ylidene), has been synthesized, and its structure has been characterized crystallographically.

## Introduction

Copper–sulfido clusters have attracted considerable interest due to their compositional and structural diversity, interesting chemical properties, and role in biochemical processes.^1–6^ The wide variety of stoichiometries and structures found for these clusters arises from the fact that both the copper and sulfur centers can possess a range of coordination numbers. Among copper(i)–sulfido clusters, for example, copper centers are found in two, three, and/or four-coordinate geometries, with coordination numbers for the sulfido ligands ranging from three to nine.^1,2,5f–k^ The size range of these clusters is correspondingly broad, with examples to date spanning Cu_3_(μ_3_-S)L_*m*_ ^2a ,b^ to Cu_136_S_56_L_*n*_ ^5*k*^ (L = ancillary ligand).

**Chart 1:**
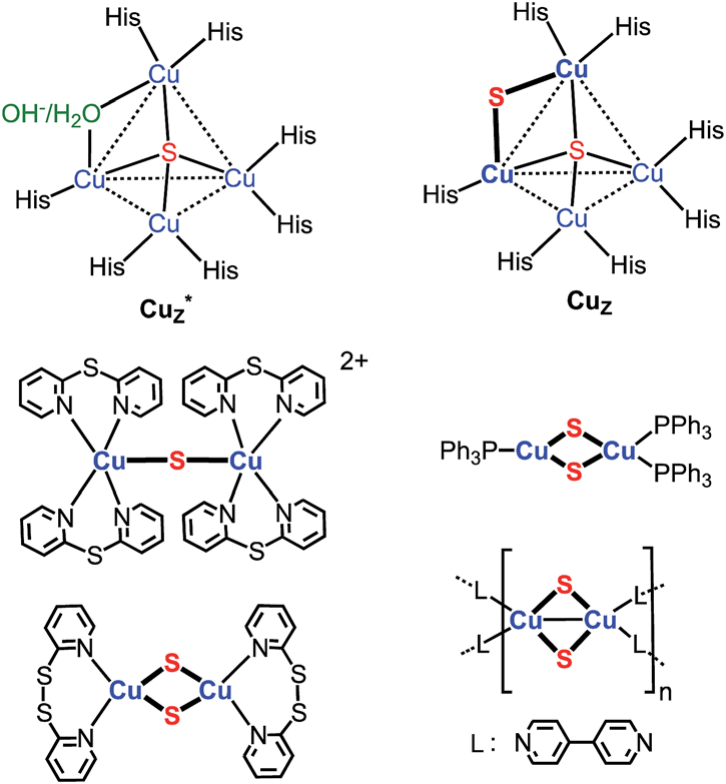
Copper–sulfido clusters in N_2_OR and reported copper sulfido complexes that contain Cu_2_(μ_2_-S) unit(s).^3a,4,6^

Implicit in this compositional richness is that it is challenging to design synthetic routes to clusters of specific nuclearity and structure. One synthetic target of interest, for example, are clusters that model the active site of nitrous-oxide reductase (N_2_OR), which catalyzes the reduction of nitrous oxide to dinitrogen and water.^6^ The histidine-ligated tetracopper cluster at this site (Chart 1) is known in two forms, one of which contains a single sulfido ligand (Cu_4_(μ_4_-S), denoted Cu*Z) and the other two sulfido ligands in the resting 2CuI2Cu^II^ redox state (Cu_4_(μ_4_-S)(μ_2_-S), denoted Cu_Z_).^6^ A few model clusters of the form Cu_4_(μ_4_-S)(μ-L)_4_ (L = phosphine, amidinate) have been synthesized that qualitatively replicate the geometry of the Cu_4_(μ_4_-S) core of Cu*Z and Cu_Z_.^2b–f,3c^ Compositional models for the Cu_2_(μ_2_-S) linkage in Cu_Z_ are also rare: there is a single example of a copper–sulfido complex that consists of the parent Cu_2_(μ_2_-S) unit, [{Cu(2,2′-dipyridylsulfide)_2_}_2_(μ_2_-S)]^2+^,^3a,19^ and three clusters that contain two singly bridging sulfido ligands, of the type Cu_2_(μ_2_-S)_2_L_*n*_ (Chart 1).^4^ None of the clusters shown in Chart 1 are available in high synthetic yield (4–37%).^3a,4^ A general challenge to preparing these and other low-nuclearity copper–sulfido clusters is inhibiting condensation of their unsaturated Cu–S units into higher-nuclearity clusters. Nevertheless, such clusters are of general interest because they should allow study of properties and reactions of Cu_*n*_S units in the absence of potentially complicating collective effects and multiple reaction sites.

**Chart 2:**
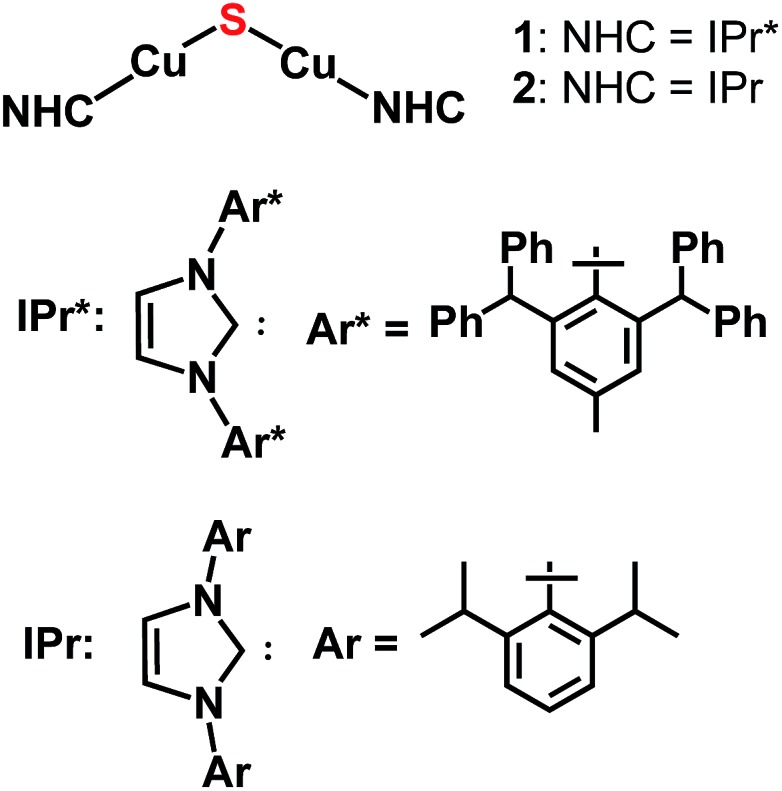
Target complexes of this study.

The lack of general synthetic routes to low-nuclearity copper–sulfido clusters motivated us to consider whether bulky N-heterocyclic carbene (NHC) ligands would provide the steric shielding necessary to suppress condensation to higher-nuclearity structures, given that NHC ligands are well-known for the ability to stabilize low-coordinate metal complexes.^7,8^ Recently, we provided support for this hypothesis with a report of the synthesis and characterization of the copper(i) cluster [{(IPr)Cu}_3_(μ_3_-S)]^+^ (IPr = 1,3-bis(2,6-di-isopropylphenyl)imidazol-2-ylidene).^2*a*^ This cluster, together with two clusters of form Cu_3_(μ_3_-S)(μ-L)_3_ reported recently by Mankad,^2*b*^ are the smallest CuI*n*(μ_*n*_-S) clusters known to date. In [{(IPr)Cu}_3_(μ_3_-S)]^+^, the steric protection provided by the IPr ligand^9^ is such that the copper centers are present in their lowest possible coordination number of two, and the cluster is stable without the bridging ancillary ligands found in all other examples of CuI*n*(μ_*n*_-S)L_*m*_ clusters.^2^ In view of this finding, we investigated whether NHC ligands would allow synthesis and stabilization of the parent Cu_2_(μ_2_-S) cluster, of which there is one example for Cu^II^ (Chart 1) and none for Cu^I^.^3*a*^ Herein, we describe three synthetic routes devised to provide {(NHC)Cu^I^}_2_(μ_2_-S) compounds (Chart 2). The NHC ligands employed are IPr and IPr* (**1**, NHC = IPr* (1,3-bis(2,6-(diphenylmethyl)-4-methylphenyl)imidazol-2-ylidene);^10^ **2**, NHC = IPr); these differ substantially from each other in steric bulk, in order to allow the relationship between stability of the complex and steric shielding to be assessed. It is found that all three synthetic routes provide these compounds as at least initial products, albeit in differing yields. Compound **1** is stable in the solid state and solution but **2** has only transient stability in solution, indicating that ancillary ligands with substantial steric bulk are necessary to stabilize these unsaturated clusters. Despite the steric protection provided by the IPr* ligands of **1**, it is found that this complex reacts with organic electrophiles *via* formal transfer of the sulfido ligand.

## Results and discussion

### Synthetic approaches to {(NHC)Cu^I^}_2_(μ_2_-S) compounds

**Scheme 1:**
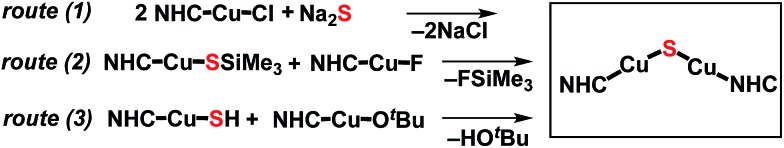
Synthetic routes to **1** and **2**.

The synthesis of the {(NHC)Cu^I^}_2_(μ_2_-S) compounds **1** and **2** was attempted using three routes, shown in Scheme 1. Route (1) is a salt metathesis reaction between (NHC)CuCl and Na_2_S. Routes (2) and (3) involve the reaction between a compound of the form (NHC)Cu(SR), in which the sulfido ligand of the ultimate product bears a protecting R group, and a (NHC)CuX compound for which X is a suitable deprotecting moiety. In route (2) the protecting group is SiMe_3_, where the reaction between (NHC)Cu(SSiMe_3_) and (NHC)CuF could lead to formation of {(NHC)Cu}_2_(μ_2_-S) *via* elimination of FSiMe_3_. This general approach has been applied in the synthesis of {(IPr)CuS}_2_Hg from the reaction between (IPr)Cu(SSiMe_3_) and Hg(OAc)_2_,^15^ of Cu_2*m*_S_*m*_L_*n*_ (L = phosphine) clusters *via* reactions between Cu(OAc) and S(SiMe_3_)_2_ in the presence of L,^1^ and by us to the synthesis of the cluster [{(IPr)Cu}_3_(μ_3_-S)][BF_4_], in which the reaction between [{(IPr)Cu}_2_(μ-SSiMe_3_)][BF_4_] and (IPr)CuF cleanly provides the product in 85% yield.^2*a*^ In route (3) the protecting group is a proton, which could be removed in an acid–base reaction between (NHC)Cu(SH) and (NHC)Cu(O^*t*^Bu) with formation of the desired product and *t*-butanol. A potential advantage to route (1) is that it uses readily available (NHC)CuCl complexes as starting materials, whereas the precursors in routes (2) and (3) must first be prepared from (NHC)CuCl. On the other hand, routes (2) and (3) could be used, in principle, to prepare mixed-ligand (NHC)Cu(μ_2_-S)Cu(NHC′) complexes, unlike route (1). The application of these approaches to the synthesis of **1** and **2** are described and compared below.

### Synthesis and characterization of **1**

Compound **1** can be prepared *via* routes (1), (2), and (3) (Scheme 1), although the purity of the crude product and final yields vary considerably. For route (1), the reaction between excess Na_2_S (2.5 equivalents) and (IPr*)CuCl^11^ in THF at 50 °C for 2 h resulted in complete consumption of the copper starting material and formation of a light-yellow product subsequently identified as **1** in 67% isolated yield. The reaction is much slower at room temperature, with only ∼50% conversion of (IPr*)CuCl to **1** being observed after 12 hours. Compound **1** is stable both in solution and the solid state under N_2_ atmosphere at room temperature for weeks.

**Fig. 1:**
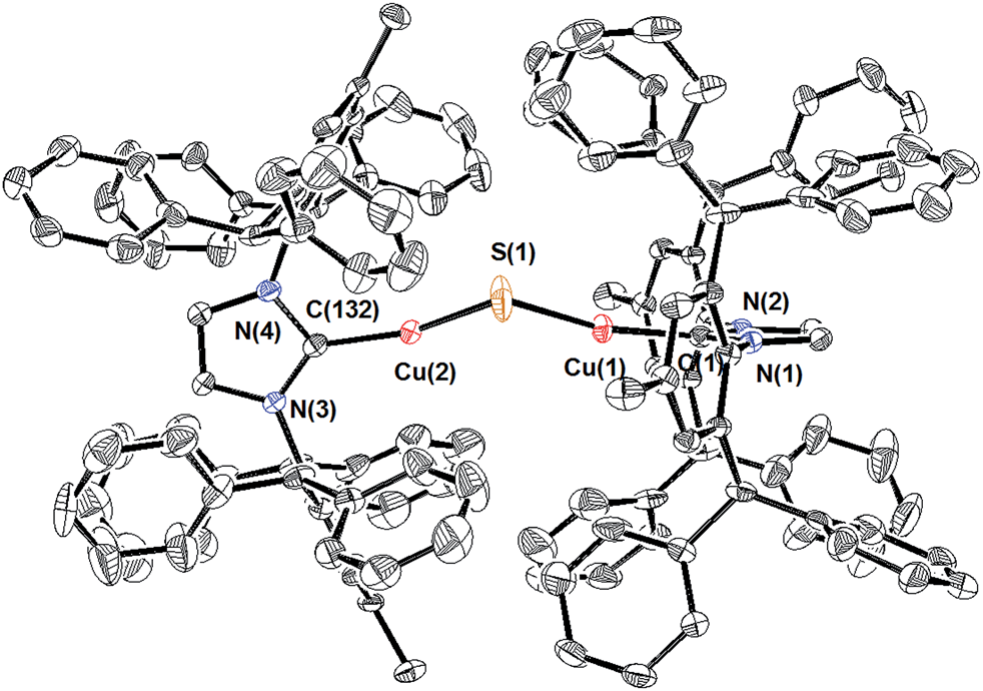
X-ray crystal structure of **1** (50% probability ellipsoids). Hydrogen atoms are omitted for clarity. Selected bond lengths (Å) and bond angles (°): Cu(1)–C(1), 1.873(5); Cu(2)–C(132), 1.869(5); Cu(1)–S(1), 2.0787(17); Cu(2)–S(1), 2.0848(18); Cu(1)···Cu(2), 3.6085(9); C(1)–Cu(1)–S(1), 162.92(16); C(132)–Cu(2)–S(1), 158.81(16); Cu(1)–S(1)–Cu(2), 120.15(9). The dihedral angle between the IPr* imidazole rings is 89.9(3)°.

The composition and structure of **1** were established by ^1^H- and ^13^C-NMR spectroscopy, elemental analysis, and X-ray crystallography (see ESI†). In solution at room temperature, the NMR resonances indicate that the two IPr* ligands are equivalent and that there is rapid rotation about the Cu–C bonds on this time scale, consistent with the compound possessing minimum *C*_2_ symmetry. The X-ray crystal structure of **1** (Fig. 1) shows the presence of two two-coordinate Cu^I^ centers connected *via* a bent Cu–S–Cu linkage (∠Cu–S–Cu = 120.15(9)°). The substantial steric requirements of the IPr* ligands are manifested in the deviation from a linear geometry at the Cu centers (∠C–Cu–S = 162.92(16)° and 158.81(16)°), and by the fact that the two imidazole rings of the IPr* ligands are perpendicular to each other (dihedral angle = 89.9(3)°). The Cu–S bond lengths and Cu–S–Cu angle of **1** differ significantly from those of the only other reported Cu_2_(μ-S) cluster, [{Cu^II^(2-dps)_2_}_2_(μ-S)]^2+^ (dps = 2,2′-dipyridylsulfide; Chart 1),^3*a*^ presumably due to their different metal oxidation states and coordination numbers, but closely resemble those of the tricopper(i) cluster [{(IPr)Cu}_3_(μ_3_-S)]^+^. In particular, the Cu–S bond distances in **1** (2.0787(17) Å and 2.0848(18) Å) are slightly shorter (by ∼0.05 Å) than the Cu–S bonds observed for [{(IPr)Cu}_3_(μ_3_-S)]^+^,^2*a*^ consistent with the smaller sulfur coordination number in **1**, but markedly shorter than those reported for [{Cu(2-dps)_2_}_2_(μ-S)]^2+^ (2.6666(7) Å).^3*a*^ Similarly, the bent Cu–S–Cu geometry (120.15(9)°) of **1** is comparable to that for [{(IPr)Cu}_3_(μ_3_-S)]^+^ (113.02(3)°)^2*a*^ and contrasts with the linear structure observed for [{Cu(2-dps)_2_}_2_(μ-S)]^2+^.^3*a*^

**Scheme 2:**
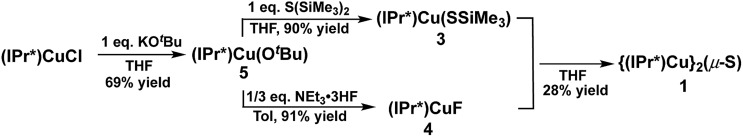
Trimethylsilyl-deprotection route to **1**.

The synthesis of **1** *via* route (2) involves the reaction between (IPr*)Cu(SSiMe_3_) (**3**) and (IPr*)CuF (**4**) (Scheme 2). Complexes **3** and **4** have not previously been reported. A logical approach to the synthesis of **3** would seem to be the reaction between (IPr*)CuCl and S(SiMe_3_)_2_, given that the analogous reaction between (IPr)CuCl and S(SiMe_3_)_2_ at room temperature for 1 hour provides the compound (IPr)Cu(SSiMe_3_) in 87% yield.^2*a*^ However, (IPr*)CuCl is observed not to react with S(SiMe_3_)_2_ under identical conditions after several days. Instead, the new complex (IPr*)Cu(O^*t*^Bu) (**5**) was found to be a suitable precursor for both **3** and **4**. Compound **5** was prepared in 69% yield from the reaction between (IPr*)CuCl and KO^*t*^Bu in THF (Scheme 2). Compound **3** is then cleanly prepared, in 90% isolated yield, from the reaction between **5** and one equivalent of S(SiMe_3_)_2_ in THF for 1 hour. Compound **4** was prepared by analogy to (IPr)CuF^12^ *via* the reaction between **5** and NEt_3_·3HF (91% yield). The compositions of **3**, **4**, and **5** were established by multinuclear NMR spectroscopy and elemental analysis (see ESI†).

Surprisingly, the reaction between an equimolar mixture of **3** and **4** in THF for 1 hour yielded multiple products, as shown by the ^1^H-NMR spectrum of the isolated crude product (see ESI†). The crude product contains **1** (∼28%, based on resonance integration), unreacted **3** (but not **4**), and one other set of IPr* signals that are not those of the free ligand, indicating it is another IPr*-containing compound. Attempts to separate these compounds by recrystallization of the crude product proved fruitless, and the identity of the secondary product remains unknown. Given the complexities of route (2) compared to route (1) for the preparation of **1**, it was not further investigated. The failure of route (2) stands in marked contrast to the success of this general approach in cleanly providing the related cluster [{(IPr)Cu}_3_(μ_3_-S)]^+^.^2*a*^

**Scheme 3:**
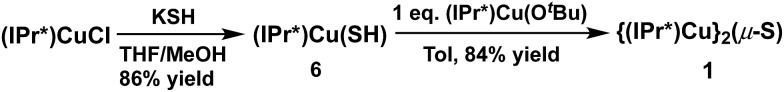
Thiolato-deprotonation route to **1**.

In contrast to route (2), the acid–base deprotection strategy employed in route (3) cleanly provides **1** (Scheme 3). The terminal thiolato complex (IPr*)Cu(SH) (**6**) was prepared in 86% isolated yield from the salt metathesis reaction between (IPr*)CuCl and KSH in methanol/THF (Scheme 3), and characterized by ^1^H- and ^13^C-NMR spectroscopy, elemental analysis, and X-ray crystallography (see ESI†). Compound **6** is a rare example (together with **7**, *vide infra*) of a terminal hydrosulfido complex of copper.^3a,13^ The reaction between **6** and **5** cleanly provides **1** in 84% isolated yield (Scheme 3). The overall yield of **1** from (IPr*)CuCl *via* route (3) is 64%, which is slightly lower than that for route (1) (67% yield). Route (1) is preferred for the synthesis of **1** because it requires fewer steps, but route (3) might be useful for the preparation of mixed-ligand (NHC)Cu(μ_2_-S)Cu(NHC′) complexes.

### Attempted synthesis of **2**

The synthesis of **2** (Chart 1) was attempted *via* the same three routes explored for the synthesis of **1** (Scheme 1) to determine whether the less bulky IPr ligand can support the Cu_2_(μ_2_-S) core. For each route, compounds of the form (IPr)CuX (X = Cl,^14^ SSiMe_3_,^2a,15^ F,^12^ O^*t*^Bu,^16^ SH (**7**)) were employed as starting materials; these are exactly analogous to the (IPr*)CuX starting materials used for **1**. The starting materials have been previously reported except for (IPr)Cu(SH) (**7**), which was prepared analogously to **6** *via* the reaction between (IPr)CuCl and KSH in THF/MeOH (77% yield) and characterized by ^1^H- and ^13^C-NMR spectroscopy, mass spectrometry, and X-ray crystallography (see ESI†). The Cu–S and Cu–C bond distances in **7** are 2.1270(12) and 1.890(4) Å, respectively, which are similar to those of other (IPr)CuSR compounds (*d*(Cu–SR) = 2.120–2.149 Å, R = alkyl, benzyl, aryl, triptycyl; *d*(Cu–C_IPr_) = 1.884–1.898 Å).^17^

**Scheme 4:**
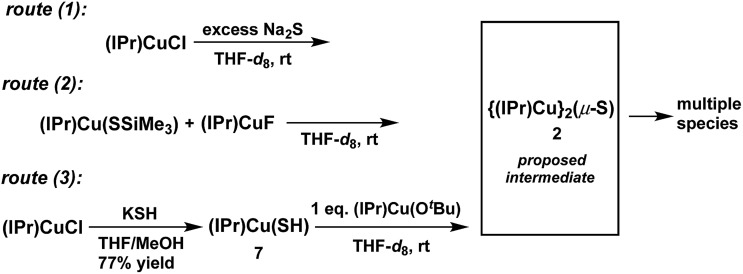
Routes for the attempted synthesis of **2**.

Reactions that implemented routes (1), (2), and (3) were performed on an NMR-tube scale in THF-*d*_8_ at room temperature and monitored by ^1^H-NMR spectroscopy. The results are summarized in Scheme 4. Within approximately 5 minutes, the ^1^H-NMR spectra of all three reaction mixtures (see ESI†) showed the presence of the same new IPr-containing species, denoted **X**,^18^ which is clearly distinguishable from the starting materials and free IPr. No ^1^H-NMR resonances are observed for **X** other than those of the IPr ligand. At the 5 minute mark, the ^1^H-NMR spectra of the three reaction mixtures showed that product **X** formed *via* routes (1) and route (3) is relatively clean, whereas route (2) produced multiple products; this parallels the observations for the synthesis of **1** by these routes. The reaction between (IPr)CuCl and Na_2_S (route (1)) contained **X** almost exclusively, together with a small amount of unreacted (IPr)CuCl (see ESI†). In contrast, the reaction between equimolar quantities of (IPr)Cu(SSiMe_3_) and (IPr)CuF (route (2)) generated **X**, a new set of IPr signals that are not attributable to the starting materials or free IPr, and a new singlet resonance centered at 0.19 ppm, which is attributed to a Me_3_Si-containing species that is neither **7** nor FSiMe_3_. The nature of these additional species is unclear. The mixture produced from the reaction between equimolar quantities of (IPr)Cu(O^*t*^Bu) and **8** (route (3)) generated **X** and ^*t*^BuOH, with the integration of the IPr and ^*t*^Bu resonances being in a 2 : 1 ratio. This latter observation suggests that **X** contains two IPr ligands, and that **X** is compound **2**.

At longer reaction times, the ^1^H-NMR spectra of the three reaction mixtures show that **2** begins to decompose (see ESI†). The decomposition products of **2** vary from reaction to reaction. For route (1) free IPr is the principal (NMR-observed) decomposition product of **2**; it is clearly evident within 30 minutes following the start of the reaction. In contrast, free IPr is not observed among the decomposition products of **2** for route (2). For route (3), decomposition of **2** provides free IPr and several other unidentified IPr-containing decomposition products. Due to its instability, attempts to isolate and characterize **2** and the accompanying products were unsuccessful.

**Fig. 2:**
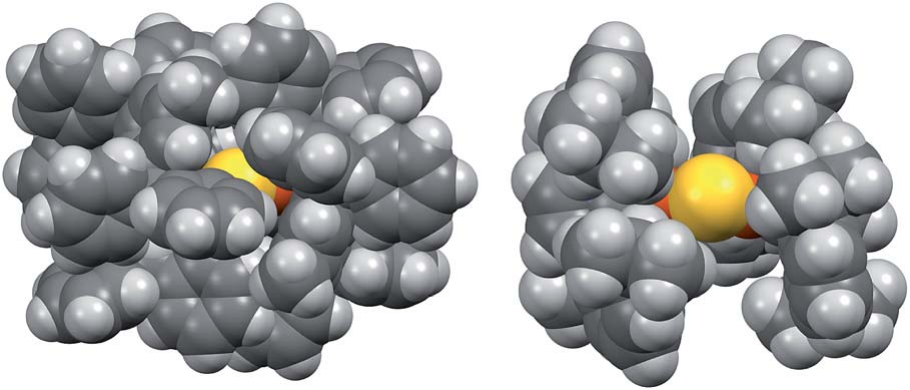
Space-filling models of **1** (left, X-ray crystallography) and **2** (right, DFT), viewed along the same direction.

The instability of **2** in solution is in stark contrast to **1**, which is stable for weeks at room temperature in solution and the solid state under nitrogen atmosphere. Given the electronic similarity of the IPr and IPr* ligands, we surmise that the substantial steric bulk of the IPr* ligands of **1** plays a key role in shielding the CuI2(μ_2_-S) core. To probe this further, the structure of **2** in the gas phase was calculated using density functional theory (see ESI†). The calculated structure of **2** resembles that determined by X-ray crystallography for **1**: it displays unremarkable Cu–C and Cu–S bond lengths and a smaller Cu–S–Cu bond angle (111° *vs.* 120° for **1**), reflecting the decreased steric demands of the IPr ligand. These metrical parameters do not point to pronounced electronic differences between **1** and **2** that would account for the instability of the latter. The space-filling models of **1** and **2**, shown in Fig. 2, suggest that the stability of **1** instead results from the substantial encapsulation of the Cu_2_S core by the IPr* ligands, whereas the IPr ligands of **2** leave the sulfido ligand exposed.

### Reactions of **1** with haloalkanes

The instability of **2** suggests that the bridging sulfido ligand of this class of compounds might be a reactive center. A DFT calculation of the frontier orbitals of **1** (see ESI†) shows that the HOMO and HOMO–1 possess substantial sulfur p orbital character, suggesting that the sulfido ligand might be reactive toward electrophiles. In this vein, previously reported dicopper(i) bridging-thiolato complexes of the form [{(IPr)Cu}_2_(μ_2_-SR)]^+^ (R = CH_2_Ph,^17*a*^ ^*t*^Bu,^17*a*^ SiMe_3_ ^2a,15^) have the appearance of the initial product that would result from formal attachment by R^+^ to the sulfido ligand of **1**. To investigate these possibilities, the reactions of **1** with haloalkanes were studied.

**Scheme 5:**
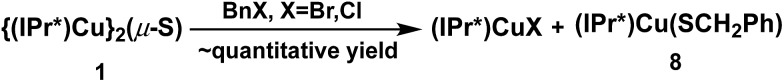
Reaction of **1** with benzyl halides.

The reactions between **1** and benzyl halides (BnBr and BnCl) were studied by ^1^H-NMR spectroscopy. Monitoring of an approximately equimolar mixture of **1** and BnBr in C_6_D_6_ at room temperature showed that BnBr was consumed in ∼1 h, with the quantitative formation of a 1 : 1 molar ratio of (IPr*)CuBr^20^ and the new compound (IPr*)Cu(SBn) (**8**) (Scheme 5). The identity of **8** was established by independent synthesis from the reaction between **5** and BnSH (see ESI†). The analogous reaction between **1** and 1.2 equivalent of BnCl forms (IPr*)CuCl and **8** but is much slower than the BnBr reaction, requiring ∼3 days to reach completion at room temperature. At higher temperature (50 °C) the reaction is complete in ∼12 h (see ESI†).

**Scheme 6:**
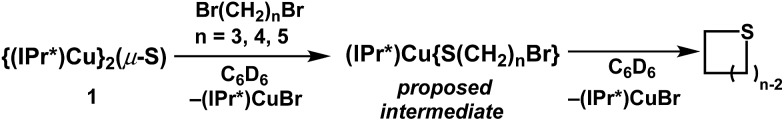
Reaction of **1** with 1,*n*-dibromoalkanes.

These clean reactions suggested that **1** might be competent to transfer the sulfido ligand to appropriate substrates. This was explored by reacting **1** with dibromo alkanes (Scheme 6). Treatment of **1** with 1.4 equivalents of 1,3-dibromopropane in C_6_D_6_ at room temperature resulted in the immediate consumption of **1** and concurrent formation of (IPr*)CuBr and a reaction intermediate, proposed as (IPr*)Cu{S(CH_2_)_3_Br} (**9**) on the basis of its ^1^H-NMR signals (*δ* 3.37 (t, SC*H*_2_), 2.85 (t, BrC*H*_2_), 1.82 (quin, CH_2_C*H*_2_CH_2_); see ESI†). Over the course of 7 h, **9** was gradually consumed, with the concomitant formation of the cyclic thioether thietane (identified by GC-MS and ^1^H-NMR spectroscopy) and (IPr*)CuBr in near quantitative yields. The reactions of **1** with 1,4-dibromobutane or 1,5-dibromopentane proceeded much faster at room temperature. Both reactions were complete within 1 h, with the formation of (IPr*)CuBr and tetrahydrothiophene or tetrahydrothiopyran in quantitative yields (see ESI†). These reactions probably involve the same pathway proposed for the reaction of **1** with 1,3-dibromopropane, although in neither case could reaction intermediates analogous to **9** be observed in the ^1^H-NMR spectrum recorded ∼5 min after the mixing of reactants in C_6_D_6_.

The mild conditions for these reactions contrast with those required for the synthesis of the same cyclic thioethers from 1,*n*-dibromoalkanes and Na_2_S.^21^ A thorough study of solvents and conditions for the latter reactions showed that optimal product yields (65–95%) are obtained in DMSO solvent at 150 °C, and that lower yields (30–75%) were obtained at lower temperatures or with use of other common solvents (C_6_H_6_, THF, EtOH, DMF), even with extended heating.^21*e*^ While the synthesis of these particular thioethers from **1** is not of practical importance, the mild conditions suggest that **1** might be useful to affect sulfido transfer to more complex organic molecules that present haloalkyl substituents. Preliminary evidence indicates that this mode of reactivity is not general to low-nuclearity copper–sulfido clusters: the related cluster [{(IPr)Cu}_3_(μ_3_-S)]^+^ shows no reactivity towards either BnBr or 1,4-dibromobutane at room temperature over five hours.

## Conclusions

The compound {(IPr*)Cu}_2_(μ-S) (**1**) is only the second example of a copper–sulfido cluster comprised of a single Cu_2_(μ-S) core and the first with the Cu^I^ oxidation state. Of the three synthetic routes tested (Scheme 1), the salt metathesis reaction between (IPr*)CuCl and Na_2_S (route (1)) is preferred due to its simplicity and good yield. The acid–base reaction between (IPr*)CuSH and (IPr*)Cu(O^*t*^Bu) (route (3)) provides **1** in comparable yield but requires more steps; however, it may be suitable for preparing mixed-ligand (NHC)Cu(μ_2_-S)Cu(NHC′) compounds. The synthetic routes that produce **1** appear also to generate **2** transiently, but it decomposes too quickly to allow isolation. The stability of **1** evidently depends upon encapsulation of the Cu_2_(μ-S) core by the bulky IPr* ligands. The comparative steric openness of **2** is evidenced by the capacity of the sulfido ligand to accommodate another (IPr)Cu^+^ unit, in the cluster [{(IPr)Cu}_3_(μ_3_-S)]^+^.^2*a*^ Despite the steric protection afforded by the IPr* ligands of **1**, it reacts with haloalkanes with resulting formation of C–S bonds.

The demonstration that the Cu_2_(μ-S) reactive core can be stabilized by ligands of suitable steric bulk suggests that other clusters of this class can be prepared with comparably sized NHC by the routes described here, and possibly with other bulky supporting ligands.
