# Supplementary material for: Synthesis, structure, and reactions of a copper–sulfido cluster comprised of the parent Cu2S unit: {(NHC)Cu}2(μ-S)
Source: Chem Sci. 2015 Oct 20;7(1):589–95. doi: 10.1039/c5sc03258j (PMC5523119; doi:10.1039/c5sc03258j)
Supplement: Supplementary file 1 [file SC-007-C5SC03258J-s001.pdf]

Electronic Supplementary Material (ESI) for Chemical Science.
This journal is © The Royal Society of Chemistry 2015

Supplementary Information

Synthesis, Structure, and Reactions of a Copper–Sulfido Cluster Comprised of the Parent Cu_2S Unit: $\{(NHC)Cu\}_2(\mu\text{-}S)$

Junjie Zhai,* Alexander S. Filatov, and Gregory L. Hillhouse and Michael D. Hopkins,
Department of Chemistry, The University of Chicago, Chicago, IL 60637

Table of Contents

1. General Considerations……S5

2. Preparation, Characterization and Reactions of Compounds……S6

Synthesis of (IPr*)Cu(O^{t}Bu) (**5**)……S6

Synthesis of (IPr*)Cu($SSiMe_3$) (**3**)……S6

Synthesis of (IPr*)CuF (**4**)……S7

Synthesis of (IPr*)Cu(SH) (**6**)……S8

Synthesis of (IPr)Cu(SH) (**7**)……S9

Synthesis of $\{(IPr^*)Cu\}_2(\mu\text{-}S)$ (**1**)……S10

Figure S1. ^{1}H-NMR spectrum of the crude product isolated from the reaction between **3** and **4** in THF……S12

Attempted Synthesis of $\{(IPr)Cu\}_2(\mu\text{-}S)$ (**2**)……S12

Figure S2. ^{1}H-NMR spectra of the reaction of (IPr)CuCl with excess Na_2S in THF-d_8……S15

Figure S3. ^{1}H-NMR spectra of the reaction between (IPr)CuF and (IPr)Cu($SSiMe_3$) in THF-d_8……S16

Figure S4. ^{1}H-NMR spectra of the reaction between (IPr)Cu(SH) and (IPr)Cu(O^{t}Bu) in THF-d_8……S17

Synthesis of (IPr*)Cu(SBn) (**8**)……S17

Reaction between $\{(IPr^*)Cu\}_2(\mu\text{-}S)$ and BnBr……S18

Figure S5. ^{1}H-NMR spectra of the reaction between $\{(IPr^*)Cu\}_2(\mu\text{-}S)$ and BnBr in C_6D_6..S19

Reaction between $\{(IPr^*)Cu\}_2(\mu\text{-}S)$ and BnCl……S19

Figure S6. ^{1}H-NMR spectra of the reaction between {(IPr*)Cu}$_2$(μ-S) and BnCl in Tol-d_8...S20

Reaction between {(IPr*)Cu}$_2$(μ-S) and 1,3-dibromopropane……………………..…...........S21

Figure S7. ^{1}H-NMR spectra of the reaction of **1** with 1,3-dibromopropane in C_6D_6............S22

Reaction between {(IPr*)Cu}$_2$(μ-S) and 1,4-dibromobutane…………………………............S22

Figure S8. ^{1}H-NMR spectrum of the reaction of **1** with 1,4-dibromobutane in C_6D_6...........S23

Reaction between {(IPr*)Cu}$_2$(μ-S) and 1,5-dibromopentane……………..………………S23

Figure S9. ^{1}H-NMR spectrum of the reaction of **1** with 1,5-dibromopentane in C_6D_6.........S24

Figure S10. ^{1}H-NMR spectrum of compound **5** in C_6D_6...S25

Figure S11. Expanded ^{1}H-NMR spectrum of compound **5** in C_6D_6…………………...........S26

Figure S12. ^{13}C{^{1}H}-NMR spectrum of compound **5** in THF-d_8………………………......S27

Figure S13. Expanded ^{13}C{^{1}H}-NMR spectrum of compound **5** in THF-d_8………..…......S28

Figure S14. ^{1}H-NMR spectrum of compound **3** in C_6D_6…………………………………......S29

Figure S15. Expanded ^{1}H-NMR spectrum of compound **3** in C_6D_6………………………......S30

Figure S16. ^{13}C{^{1}H}-NMR spectrum of compound **3** in CD_2Cl_2…………………………......S31

Figure S17. Expanded ^{13}C{^{1}H}-NMR spectrum of compound **3** in CD_2Cl_2……..……......S32

Figure S18. ^{1}H-NMR spectrum of compound **4** in CD_2Cl_2………………………………......S33

Figure S19. Expanded ^{1}H-NMR spectrum of compound **4** in CD_2Cl_2…………….....…......S34

Figure S20. ^{13}C{^{1}H}-NMR spectrum of compound **4** in CD_2Cl_2…………………………......S35

Figure S21. Expanded ^{13}C{^{1}H}-NMR spectrum of compound **4** in CD_2Cl_2...………….......S36

Figure S22. ^{19}F-NMR spectrum of compound **4** in CD_2Cl_2………………....……………....S37

Figure S23. ^{1}H-NMR spectrum of compound **6** in C_6D_6……………………………….......S38

Figure S24. Expanded ^{1}H-NMR spectrum of compound **6** in C_6D_6……..………………......S39

Figure S25. $^{13}C\{^{1}H\}$-NMR spectrum of compound **6** in CD_2Cl_2…………………………..S40

Figure S26. Expanded $^{13}C\{^{1}H\}$-NMR spectrum of compound **6** in CD_2Cl_2...……………..S41

Figure S27. ^{1}H-NMR spectrum of compound **7** in THF-d_8………………………….……..S42

Figure S28. Expanded ^{1}H-NMR spectrum of compound **7** in THF-d_8…...………………..S43

Figure S29. $^{13}C\{^{1}H\}$-NMR spectrum of compound **7** in THF-d_8…………………………..S44

Figure S30. Expanded $^{13}C\{^{1}H\}$-NMR spectrum of compound **7** in THF-d_8……...………..S45

Figure S31. ^{1}H-NMR spectrum of compound **1** in C_6D_6……...…………………..……..S46

Figure S32. Expanded ^{1}H-NMR spectrum of compound **1** in C_6D_6……...…...……..……..S47

Figure S33. ^{1}H-NMR spectrum of compound **1** in THF-d_8..…….......................................S48

Figure S34. Expanded ^{1}H-NMR spectrum of compound **1** in THF-d_8..…...........................S49

Figure S35. $^{13}C\{^{1}H\}$-NMR spectrum of compound **1** in THF-d_8……………………..........S50

Figure S36. Expanded $^{13}C\{^{1}H\}$-NMR spectrum of compound **1** in THF-d_8……….........S51

Figure S37. ^{1}H-NMR spectrum of compound **8** in C_6D_6………………...……….........S52

Figure S38. Expanded ^{1}H-NMR spectrum of compound **8** in C_6D_6……………………..…....S53

Figure S39. $^{13}C\{^{1}H\}$-NMR spectrum of compound **8** in CD_2Cl_2…………………….…....S54

Figure S40. Expanded $^{13}C\{^{1}H\}$-NMR spectrum of compound **8** in CD_2Cl_2…...…….…....S55

3. X-ray Data Collection and Crystal Structure Refinement…………….....…...……………S56

Figure S41. X-ray crystal structure of **1**…...………………………………………..…...S57

Table S1. Crystal and refinement data for **1**……………………………...……...…....S58

Figure S42. X-ray crystal structure of **6**……………….……………….……………..…...S59

Table S2. Crystal and refinement Data for **6**·CH_2Cl_2……..……………….……………....S60

Figure S43. X-ray crystal structure of **7**……………….……………….……………..…...S61

Table S3. Crystal and refinement Data for **7**……...…………………….……………….....S62

4. DFT calculations……………………………………………………………………………S63

Figure S44. DFT (B3LYP/6–31G*) optimized structure of **1**…………………...…………S64

Table S4. Comparison of selected bond distances and bond angles in the X-ray structure of **1** and optimized structure of **1** and **2**......…………….……………………………….............S64

Figure S45. DFT calculated HOMO and HOMO−1 of **1**……..S65

Figure S46. DFT (B3LYP/6–31G*) optimized structure of **2**…………………...…………S65

Table S5. Calculated (DFT) Cartesian coordinates of **1**...……...S66

Table S6. Calculated (DFT) Cartesian coordinates of **2**...……...S69

5. References……………………………………………………………………………..S71

1. General Considerations

Unless stated otherwise, all operations were performed in an MBraun *Lab Master* dry box under an atmosphere of purified nitrogen.[1] Anhydrous diethyl ether (Fisher) and benzene (Sigma Aldrich) were stirred over sodium metal and filtered through activated alumina. Anhydrous THF (Fisher) was refluxed with sodium in the presence of benzophenone for several days under N_2 atmosphere until the solution was purple, and then collected by distillation. Methylene chloride (Fisher) was dried by passage through activated alumina columns. Pentane and toluene (Fisher) were dried by passage through activated alumina and Q-5 columns. THF-d_8 (Cambridge Isotope Laboratories) was dried over sodium. CD_2Cl_2, toluene-d_8 and C_6D_6 (Cambridge Isotope Laboratories) were degassed by three cycles of freeze–pump–thaw and dried over CaH_2. Celite was activated by heating to 180 ºC while under dynamic vacuum for 12 h. (IPr*)CuCl,[2] (IPr*)CuBr,[3] (IPr)CuCl,[4] (IPr)CuF,[5] (IPr)Cu(O^{t}Bu)[6] and (IPr)Cu(SSiMe$_3$)[7] were synthesized according to standard procedures. Other chemicals were procured commercially and used as received. Elemental analyses were performed by Midwest Microlab (Indianapolis, IN) or Robertson Microlit (Ledgewood, NJ). NMR spectra were recorded on a Bruker 400 or 500 MHz NMR spectrometer; chemical shifts were measured with reference to solvent resonances (^{1}H and ^{13}C)[8] or an external standard (^{19}F: C_6F_6, δ –164.9). ESI-MS spectra and GC-MS spectra were acquired using an Agilent 6130 LC-MS and a Varian Saturn 2200 GC-MS, respectively.

2. Preparation and Characterization of Compounds

Synthesis of (IPr*)Cu(O^{t}Bu) (5). To a stirred, room temperature THF (15 mL) solution of (IPr*)CuCl[2] (0.420 g, 0.415 mmol) in a 50 mL round bottom flask, a THF (5 mL) solution of KO^{t}Bu (0.049 g, 0.44 mmol) was added via pipette. The solution gradually turned from light yellow to brown and a white precipitate formed. After stirring at room temperature for 1 h, the reaction mixture was evaporated to dryness under vacuum to give brown solids. The solid was extracted with toluene (15 mL), and the resulting mixture was filtered through celite. Addition of pentane (40 mL) to the filtrate induced partial precipitation of the product, the amount of which increased by cooling the mixture to –45 ºC in the glovebox refrigerator for 1 h. The off-white powder was collected by filtration via a glass frit and dried under vacuum for 1 h to afford 0.299 g of **5** (69% yield). ^{1}H NMR (22 ºC, 400 MHz, C_6D_6): δ 7.55 (d, 8H, $^3J_{HH}$ = 7.6 Hz, *o*-C_6H_5), 7.25 (t, 8H, $^3J_{HH}$ = 8.0 Hz, *m*-C_6H_5), 7.07–6.91 (m, 28H, -C_6H_5 and -$C_6H_2CH_3$), 5.67 (s, 4H, -C*H*Ph_2), 5.40 (s, 2H, -NC*H*=C*H*N-), 1.74 (s, 6H, -$C_6H_2CH_3$), 1.56 (s, 9H, -C(CH_3)$_3$). ^{13}C{^{1}H} NMR (24 ºC, 126 MHz, THF-d_8): δ 184.2 (*C*Cu), 144.2, 143.9, 142.2, 140.4, 136.0, 130.9, 130.8, 130.3, 129.1, 129.0, 127.2, 127.1, 123.7, 69.44 (-*C*(CH_3)$_3$), 52.23 (-*C*HPh_2), 37.22 (-C(CH_3)$_3$), 21.62 (-$C_6H_2CH_3$). Anal. Calcd. for $C_{73}H_{65}CuN_2O$: C, 83.51%; H, 6.24%; N, 2.67%. Found: C, 83.26%; H, 6.32%; N, 2.60%.

Synthesis of (IPr*)Cu(SSiMe$_3$) (3). Two 20 mL vials, one each containing a THF (10 mL) solution of **5** (0.688 g, 0.655 mmol) and a THF (3 mL) solution of S(SiMe$_3$)$_2$ (0.117 g, 0.656 mmol), were cooled to –45 ºC in the glovebox refrigerator. After removing the vials from the refrigerator, the solution of S(SiMe$_3$)$_2$ was added dropwise via pipette to the solution of **5** with stirring. The reaction solution was stirred at room temperature for 1 h, during which time it

turned from brown to greenish blue. The solution was evaporated to dryness under reduced pressure. The resulting green solid was triturated with pentane (3 mL). The mixture was filtered through a glass frit. The white powder collected on the frit was washed with pentane (2 × 3 mL) and dried under vacuum for 1 h to give 0.636 g of **3** (yield: 90%). ^{1}H NMR (22 ºC, 500 MHz, C_6D_6): δ 7.54 (d, 8H, $^{3}J_{HH}$ = 7.5 Hz, *o*-C_6H_5), 7.29 (t, 8H, $^{3}J_{HH}$ = 7.5 Hz, *m*-C_6H_5), 7.05 (t, 4H, $^{3}J_{HH}$ = 7.5 Hz, *p*-C_6H_5), 7.02 (s, 4H, -$C_6H_2CH_3$), 6.99–6.90 (m, 20H, -C_6H_5), 5.65 (s, 4H, -C*H*Ph$_2$), 5.38 (s, 2H, -NC*H*=C*H*N-), 1.73 (s, 6H, -$C_6H_2CH_3$), 0.38 (s, 9H, -Si(C*H*$_3$)$_3$). ^{13}C{^{1}H} NMR (24 ºC, 126 MHz, CD_2Cl_2): δ 181.7 (*C*Cu), 143.4, 143.2, 141.4, 140.5, 134.7, 130.4, 130.3, 129.6, 128.9, 128.7, 126.9(8), 126.9(7), 123.2, 51.78 (-*C*HPh$_2$), 21.84 (-C_6H_2*C*H$_3$), 7.39 (-Si(*C*H$_3$)$_3$). Anal. Calcd. for $C_{72}H_{65}CuN_2SSi$: C, 79.92%; H, 6.06%; N, 2.59%. Found: C, 79.52%; H, 5.98%; N, 2.51%.

Synthesis of (IPr*)CuF (4). A benzene (40 mL) solution of **5** (1.30 g, 1.24 mmol) was prepared in a 50 mL round bottom flask in the glove box. The flask was sealed with a septum and removed from the glove box. To this solution, $NEt_3 \cdot 3HF$ (0.06 mL, 0.368 mmol) was added dropwise via syringe with stirring at room temperature. A slight lightening of the light-yellow color of the solution was observed. The solution was stirred at room temperature for 4 h, and the flask was returned to the glove box. The solution was evaporated to dryness under reduced pressure, and then treated with 5 mL of toluene and 10 mL pentane. The resulting slurry was filtered via a glass frit. The white powder collected on the frit was dried under vacuum for 1 h to afford 1.12 g of **4** (yield: 91%). ^{1}H NMR (22 ºC, 500 MHz, CD_2Cl_2): δ 7.26–7.13 (m, 24H, -C_6H_5), 7.06 (d, 8H, $^{3}J_{HH}$ = 7.0 Hz, *o*-C_6H_5), 6.92 (s, 4H, -$C_6H_2CH_3$), 6.90–6.84 (m, 8H, -C_6H_5), 5.74 (s, 2H, -NC*H*=C*H*N-), 5.24 (s, 4H, -C*H*Ph$_2$), 2.25 (s, 6H, -$C_6H_2CH_3$). ^{13}C{^{1}H} NMR (23 ºC,

126 MHz, CD_2Cl_2): δ 180.9 (d, $^{2}J_{CF}$ = 35.2 Hz, *C*Cu), 143.4, 143.0, 141.3, 140.5, 134.8, 130.5, 130.0, 129.7, 128.9, 128.8, 127.1, 127.0, 123.7, 51.73 (-*C*HPh$_2$), 21.85 (-C_6H_2*C*H$_3$). ^{19}F NMR (22 ºC, 471 MHz, CD_2Cl_2): δ –236.8 (s). Anal. Calcd. for $C_{69}H_{56}CuN_2F$: C, 83.23%; H, 5.67%; N, 2.81%. Found: C, 82.81%; H, 5.92%; N, 2.67%.

Synthesis of (IPr*)Cu(SH) (6). Two 20 mL vials, one each containing a THF (5 mL) solution of (IPr*)CuCl[2] (0.191 g, 0.189 mmol) and a MeOH (2 mL) solution of KSH (0.014 g, 0.194 mmol), were cooled to –45 ºC in the glovebox refrigerator. After removing the vials from the refrigerator, the solution of KSH was added dropwise via pipette to the solution of (IPr*)CuCl with stirring. The yellow reaction mixture was stirred at room temperature for 1 h, during which time a fine white precipitate formed and the color of the solution darkened slightly. The mixture was evaporated to dryness under reduced pressure. The solid was extracted with 10 mL of toluene, and the resulting slurry was filtered through celite. Solvent was removed from the filtrate under reduced pressure to yield white powder, which was then triturated with Et_2O (3 mL). The mixture was filtered through a glass frit. The white powder collected on the frit was washed with Et_2O (3 mL) and pentane (3 mL) and dried under vacuum for 1 h to afford 0.163 g of **7** (86% yield). Colorless single crystals suitable for X-ray diffraction studies were grown from a concentrated CH_2Cl_2 solution of **6** layered with pentane at room temperature. ^{1}H NMR (23 ºC, 400 MHz, C_6D_6): δ 7.44 (d, 8H, $^{3}J_{HH}$ = 8.0 Hz, *o*-C_6*H*$_5$), 7.19 (t, 8H, $^{3}J_{HH}$ = 7.6 Hz, *m*-C_6*H*$_5$), 7.08–6.94 (m, 28H, -C_6*H*$_2$CH$_3$ & -C_6*H*$_5$), 5.59 (s, 4H, -C*H*Ph$_2$), 5.54 (s, 2H, -NC*H*=C*H*N-), 1.68 (s, 6H, -C_6H_2C*H*$_3$), –1.22 (s, 1H, -S*H*). ^{13}C{^{1}H} NMR (24 ºC, 126 MHz, CD_2Cl_2): δ 181.7 (*C*Cu), 143.3, 143.0, 141.4, 140.4, 134.7, 130.4, 130.0, 129.7, 128.9, 128.8, 127.1, 127.0, 123.5,

51.62 (-*C*HPh$_2$), 21.89 (-C_6H_2*C*H$_3$). Anal. Calcd. for $C_{69}H_{57}CuN_2S$: C, 82.07%; H, 5.69%; N, 2.77%. Found: C, 81.79%; H, 5.87%; N, 2.61%.

Synthesis of (IPr)Cu(SH) (7). Two 20 mL vials, one each containing a THF (5 mL) solution of (IPr)CuCl[4] (0.092 g, 0.19 mmol) and a MeOH (1 mL) solution of KSH (0.014 g, 0.19 mmol), were cooled to –45 °C in the glovebox refrigerator. After removing the vials from the refrigerator, the solution of KSH was added dropwise via pipette to the solution of (IPr)CuCl with stirring. The reaction mixture changed from light yellow to orange-brown, and was stirred at room temperature for 1 h, during which time a fine white precipitate formed. The mixture was evaporated to dryness under reduced pressure. The solid was extracted with THF (3 × 2 mL), and the resulting slurry was filtered through celite. Solvent was removed from the combined filtrate under reduced pressure to yield orange powder, which was then triturated with Et_2O (3 mL). The mixture was filtered through a glass frit. The white powder collected on the frit was washed with Et_2O (3 mL) and pentane (3 mL) and dried under vacuum for 1 h to afford 0.070 g of (IPr)Cu(SH) (77% yield). Colorless single crystals suitable for X-ray diffraction studies were grown from a concentrated THF/pentane solution of **7** at –45 °C. ^{1}H NMR (23 °C, 500 MHz, THF-d_8): δ 7.49–7.43 (m, 4H, *p*-$C_6H_3{}^i$Pr$_2$ and -NC*H*=C*H*N-), 7.33 (d, 4H, $^3J_{HH}$ = 8.0 Hz, *m*-$C_6H_3{}^i$Pr$_2$), 2.65 (sept., 4H, $^3J_{HH}$ = 7.0 Hz, -C*H*(CH$_3$)$_2$), 1.31 (d, 12H, $^3J_{HH}$ = 7.0 Hz, -CH(C*H*$_3$)$_2$), 1.22 (d, 12H, $^3J_{HH}$ = 7.0 Hz, -CH(C*H*$_3$)$_2$), –2.73 (s, 1H, -S*H*). ^{13}C{^{1}H} NMR (23 °C, 126 MHz, THF-d_8): δ 182.8 (*C*Cu), 146.6 (*ortho*-C), 136.0 (*ipso*-C), 130.9 (*para*-C), 124.7 (*meta*-C), 124.2 (-N*C*H=*C*HN-), 29.57 (-*C*H(CH$_3$)$_2$), 25.15 (-CH(*C*H$_3$)$_2$), 24.00 (-CH(*C*H$_3$)$_2$).[9] ESI-MS (CH$_3$CN, positive ion), *m*/*z*: 935.4 ([{(IPr)Cu}$_2$(SH)]$^+$), 492.3 ([(IPr)Cu(NCCH$_3$)]$^+$), 389.3 ([IPrH]$^+$); calculated, 935.4, 492.2 and 389.3, respectively. C, H, N elemental analyses of crystalline

samples provided carbon analyses that were not in satisfactory agreement with expectation. Composition and connectivity were confirmed via X-ray crystallography.

Synthesis of {(IPr*)Cu}$_2$(*μ*-S) (1). ***Route (1)***: To a THF (20 mL) solution of (IPr*)CuCl[2] (0.170 g, 0.168 mmol) in a 100 mL heavy-walled Schlenk tube equipped with a magnetic stir bar, anhydrous Na_2S powder (0.033 g, 0.42 mmol) was added. The tube was then sealed, taken out of glovebox, and immersed in a 50 ºC oil bath for 2 h, during which time the yellow suspension was stirred vigorously. The reaction mixture was allowed to cool to room temperature, and the Schlenk tube was returned to the glovebox. The reaction mixture was filtered through celite and the filtrate was evaporated to dryness under reduced pressure. The resulting yellow powder was triturated with Et_2O (3 mL). The mixture was filtered through a glass frit. The light yellow powder collected on the frit was washed with Et_2O (3 mL) and pentane (3 mL) and dried under vacuum for 1 h to afford 0.112 g of **1** (67% yield). ***Route (2)***: Two 20 mL vials containing separate THF (5 mL) solutions of **3** (0.109 g, 0.101 mmol) and **4** (0.100 g, 0.100 mmol) were cooled to –45 ºC in the glovebox refrigerator. After removing the vials from the refrigerator, the solution of **4** was added via pipette to the solution of **3** with stirring. The reaction mixture was stirred at room temperature for 1 h, during which time a darkening of the light yellow color was observed. The reaction mixture was evaporated to dryness under reduced pressure. The resulting light yellow solid was triturated with Et_2O (1 mL). The mixture was filtered through a glass frit. The light yellow powder collected on the frit was washed with Et_2O (2 × 1 mL) and dried under vacuum for 1 h to afford 0.163 g of crude product. The crude product was dissolved in C_6D_6 and studied by ^{1}H- and ^{19}F-NMR spectroscopy at room temperature. The ^{19}F-NMR spectrum is silent, indicating **4** is absent. The presence of **1** is

confirmed by the ^{1}H-NMR spectrum, with an estimated yield of ~28% based on integration (Figure S1). The ^{1}H NMR spectrum shows that some unreacted **3** is present (Figure S1). In addition to **1** and **3**, the crude product contains another IPr*-containing species, with singlet resonances at 1.68, 5.53 and 5.56 ppm, a triplet resonance centered at 7.20 ($^3J_{HH}$ = 7.5 Hz) and a doublet resonance centered at 7.38 ppm ($^3J_{HH}$ = 7.5 Hz) (Figure S1). Given that the intensities of the resonances integrate as ~6(s):4(s):2(s):8(t):8(d), they are assigned to the -CH_3 (s), -CHPh$_2$ (s), -NCH=CHN- (s), and Ph (d, t) groups in the IPr* ligand. Identification of the rest IPr* resonances was unsuccessful due to the signal overlap with those in **1** and **3**. Attempts to separate products from the crude mixture by recrystallization from THF/pentane several times proved fruitless. The identity of the new IPr*-containing species remains unclear. ***Route (3)***: Vials containing separate toluene (3 mL) solutions of **6** (0.104 g, 0.103 mmol) and **5** (0.108 g, 0.103 mmol) were cooled to –45 ºC in the glovebox refrigerator. After removing the vials from the refrigerator, the solution of **5** was added via pipette to the solution of **6** with stirring. The reaction solution was stirred at room temperature for 1 h, during which time it changed from colorless to light yellow. The reaction mixture was concentrated to ~1 mL under reduced pressure, and 1 mL of pentane was added to the resulting slurry to induce more precipitation. After filtration through a glass frit, the light yellow powder that was collected was washed with Et$_2$O (2 × 3 mL) and pentane (3 mL) and dried under vacuum for 1 h to afford 0.171 g of **1** (84% yield). Light-yellow single crystals suitable for X-ray studies were grown from slow diffusion of pentane vapor to the toluene solution of **1** at room temperature. ^{1}H NMR (23 ºC, 500 MHz, C$_6$D$_6$): δ 7.81 (d, 16H, $^3J_{HH}$ = 7.5 Hz, o-C$_6H_5$), 7.15 (s, 8H, -C$_6H_2$CH$_3$), 7.06 (t, 16H, $^3J_{HH}$ = 7.5 Hz, m-C$_6H_5$), 7.03–6.94 (m, 40H, -C$_6H_5$), 6.85 (t, 8H, $^3J_{HH}$ = 7.5 Hz, p-C$_6H_5$), 5.87 (s, 8H, -CHPh$_2$), 5.51 (s, 4H, -NCH=CHN-), 1.92 (s, 12H, -C$_6$H$_2$CH_3). ^{1}H NMR (22 ºC, 500 MHz, THF-d_8): δ 7.43 (d, 16H,

$^3J_{HH}$ = 8.0 Hz, *o*-C_6H_5), 7.14–7.03 (m, 24H, -C_6H_5), 6.91 (s, 8H, -$C_6H_2CH_3$), 6.89–6.80 (m, 32H, -C_6H_5), 6.74 (t, 8H, $^3J_{HH}$ = 7.5 Hz, *p*-C_6H_5), 5.58 (s, 8H, -C*H*Ph$_2$), 5.57 (s, 4H, -NC*H*=C*H*N-), 2.13 (s, 12H, -$C_6H_2CH_3$). $^{13}C\{^1H\}$ NMR (24 °C, 126 MHz, THF-d_8): δ 184.8 (*C*Cu), 144.4, 144.2, 142.6, 139.5, 136.5, 131.2, 130.8, 130.5, 129.1, 128.9, 126.9, 126.6, 122.9, 52.04 (-*C*HPh$_2$), 21.82 (-$C_6H_2CH_3$). Anal. Calcd. for $C_{138}H_{112}Cu_2N_4S$: C, 83.48%; H, 5.69%; N, 2.82%. Found: C, 83.26%; H, 5.72%; N, 2.81% (Note: the sample for elemental analysis was prepared from route (1)).

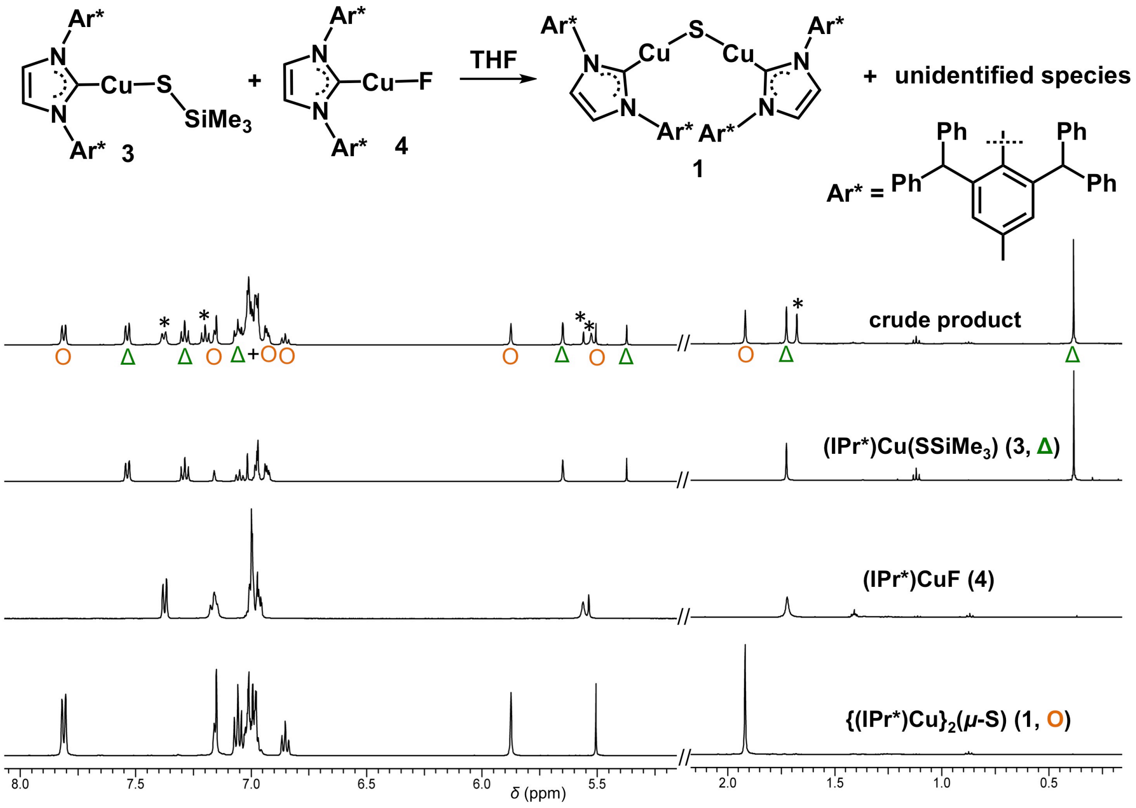

Figure S1 ^{1}H-NMR spectrum of the crude product isolated from the reaction between equimolar quantities of **3** and **4** in THF. The ^{1}H-NMR spectra of **1**, **3** and **4** are shown for reference. All spectra are recorded in C_6D_6 at ambient temperature. Resonances in the crude product assigned to **1** and **3** are labeled with O and Δ respectively; those marked with asterisks are assigned to the unidentified IPr*-containing species.

Attempted Synthesis of {(IPr)Cu}$_2$(*μ*-S) (2). ***Route (1)***: To a THF-d_8 (~0.5 mL) solution of (IPr)CuCl[4] (0.026 g, 0.053 mmol) in a J-Young NMR tube, anhydrous Na_2S powder (0.042 g,

0.54 mmol) was added. The tube was then sealed, vigorously shaken, taken out of glovebox, and studied by ^{1}H-NMR spectroscopy (Figure S2). At t ≈ 5 min, the ^{1}H-NMR spectrum of the reaction mixture exhibited the formation of a new set of IPr resonances that are different from those of (IPr)CuCl and free IPr[10], and attributed to a product denoted **X**. Besides **X**, the reaction mixture contains a small amount of unreacted (IPr)CuCl. At t = 30 min, (IPr)CuCl was almost completely consumed. The decomposition of **X** to free IPr and some unidentified species was observed at longer reaction times. At t ≈ 18.7 h, almost complete decomposition of **X** was reached and free IPr was nearly the exclusive decomposition product observed in the ^{1}H-NMR spectrum. The instability of **X** in solution hindered the isolation and further characterization of it (*e.g.* by X-ray crystallography). ***Route (2)***: To a THF-d_8 (~0.5 mL) solution of (IPr)Cu(SSiMe$_3$) (0.022 g, 0.039 mmol) in a J-Young NMR tube, a THF-d_8 (~0.5 mL) solution of (IPr)CuF (0.019 g, 0.040 mmol) was added. The tube was then sealed, vigorously shaken, taken out of glovebox, and studied by ^{1}H-NMR spectroscopy (Figure S3). At t ≈ 5 min, the ^{1}H-NMR spectrum of the reaction mixture exhibited the resonances of **X**, two new doublet resonances centered at 1.31 ppm and 1.22 ppm and multiplet resonances in the 7.3–7.5 ppm range that are not attributable to starting materials or free IPr, and a new singlet resonance at 0.19 ppm, which is attributed to a new Me$_3$Si-containing species that differs from **7** and Me$_3$SiF. Slow decomposition of **X** was observed at longer reaction times that afforded multiple unidentified species. In contrast with what was observed in route (1), free IPr was not formed in the decomposition process. Given the complexity of the reaction, it was not further investigated. ***Route (3)***: To a THF-d_8 (~0.5 mL) solution of **7** (0.011 g, 0.023 mmol) in a J-Young NMR tube, a THF-d_8 (~0.5 mL) solution of (IPr)Cu(O^{t}Bu) (0.012 g, 0.023 mmol) was added. The tube was then sealed, vigorously shaken, taken out of glovebox, and studied by ^{1}H-NMR spectroscopy (Figure S4). At t ≈ 5 min, **X** and

^{t}BuOH (δ 1.12, s, CH_3) were formed almost exclusively with the integration of the IPr and ^{t}Bu resonances being a 2:1 ratio which suggests that **X** contains two IPr ligands, and that **X** is compound **2**. Decomposition of **X** to free IPr and multiple unidentified species was observed at t = 20 h. In all three routes, grey precipitates were formed during the reaction. The identity of these precipitates is unclear. For **X**, ^{1}H NMR (22 ºC, 500 MHz, THF-d_8): δ 7.35 (t, 2H, $^{3}J_{HH}$ = 7.5 Hz, *p*-$C_6H_3{}^{i}Pr_2$), 7.16 (d, 2H, $^{3}J_{HH}$ = 7.5 Hz, *m*-$C_6H_3{}^{i}Pr_2$), 7.11 (s, 2H, -NC*H*=C*H*N-), 2.62 (sept., 4H, $^{3}J_{HH}$ = 7.0 Hz, -C*H*(CH$_3$)$_2$), 1.15 (d, 12H, $^{3}J_{HH}$ = 7.0 Hz, -CH(CH_3)$_2$), 1.10 (d, 12H, $^{3}J_{HH}$ = 7.0 Hz, -CH(CH_3)$_2$).

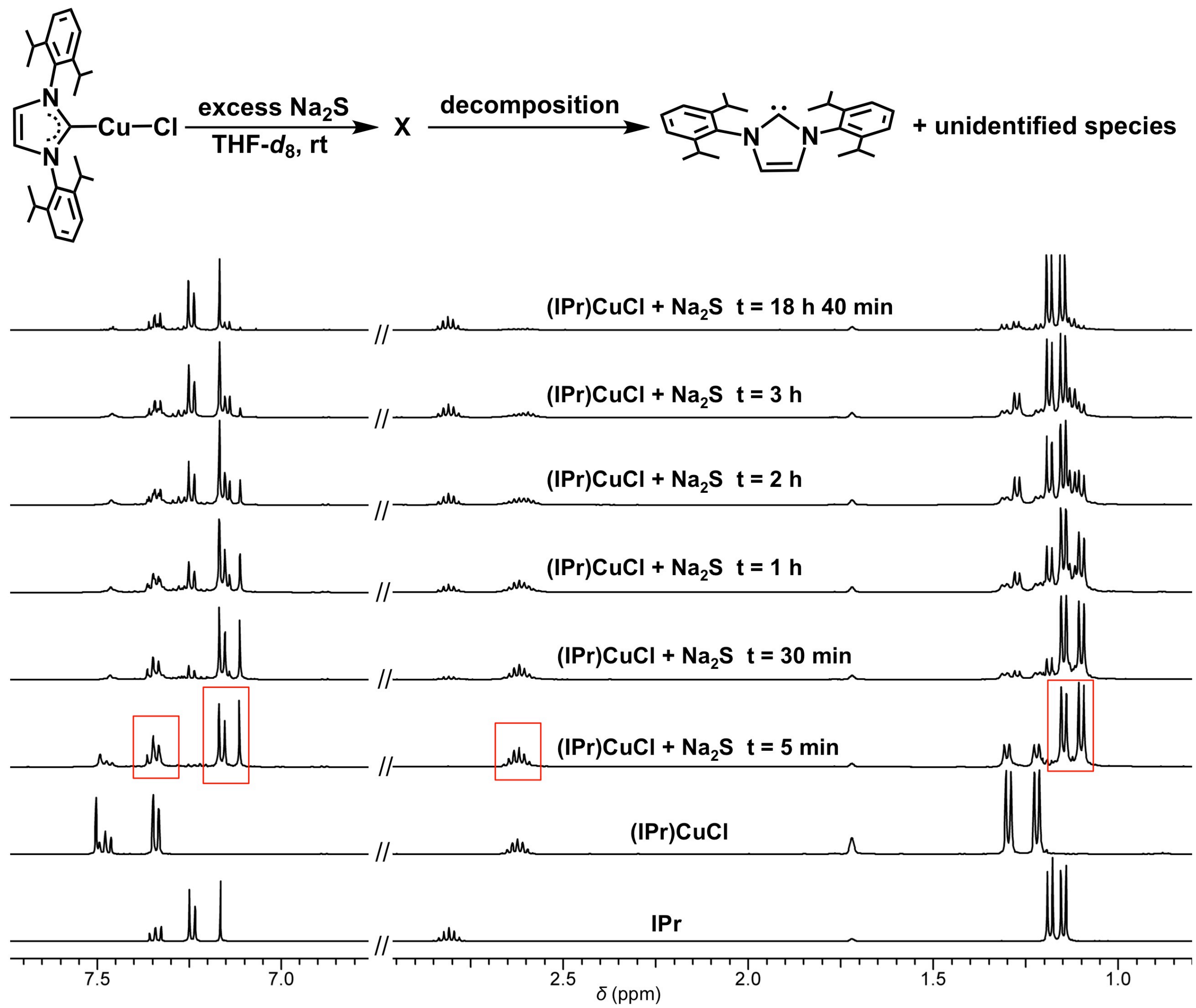

Figure S2. ^{1}H-NMR spectra of the reaction of (IPr)CuCl with excess Na_2S in THF-d_8 at t ≈ 5 min, 30 min, 1 h, 2 h, 3 h and 18 h 40 min. The ^{1}H-NMR spectra of (IPr)CuCl and IPr in THF-d_8 are given at bottom as the references. The resonances in red rectangles are assigned to **X**.

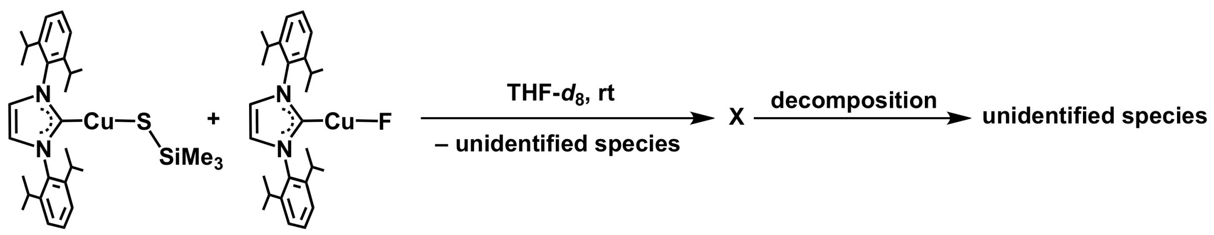

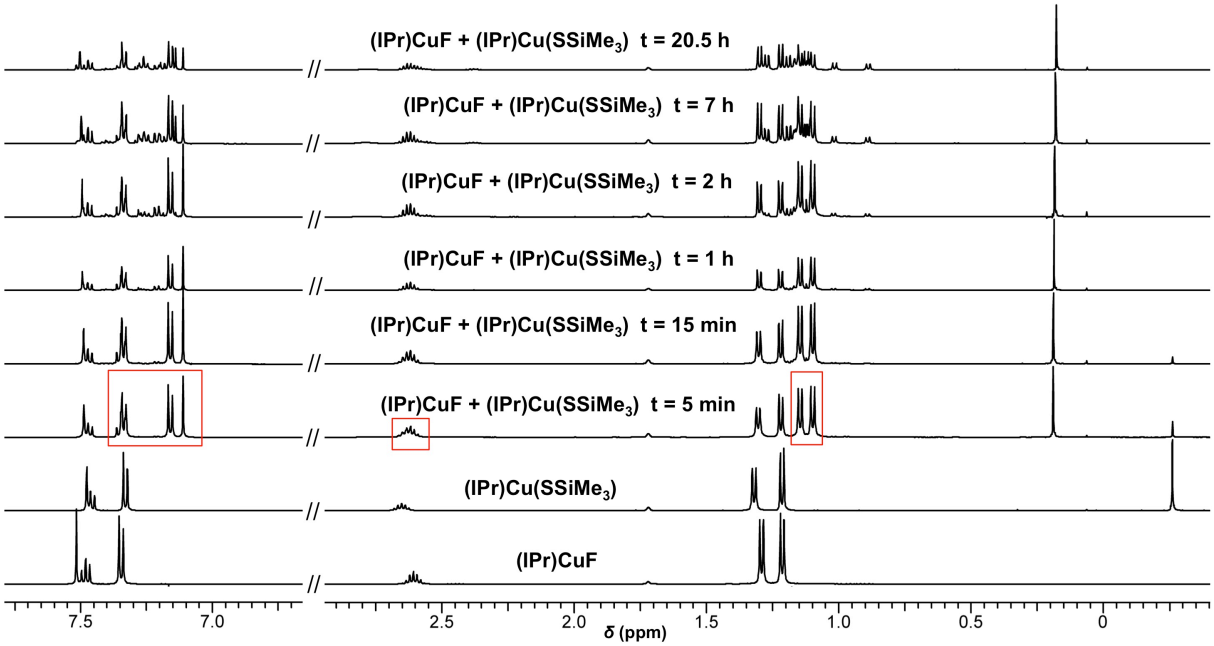

Figure S3. ^{1}H-NMR spectra of the reaction between (IPr)CuF and (IPr)Cu(SSiMe$_3$) in THF-d_8 at t ≈ 5 min, 15 min, 1 h, 2 h, 7 h and 20.5 h. The ^{1}H-NMR spectra of (IPr)Cu(SSiMe$_3$) and (IPr)CuF in THF-d_8 are given at bottom as the references. The resonances in red rectangles are assigned to **X**.

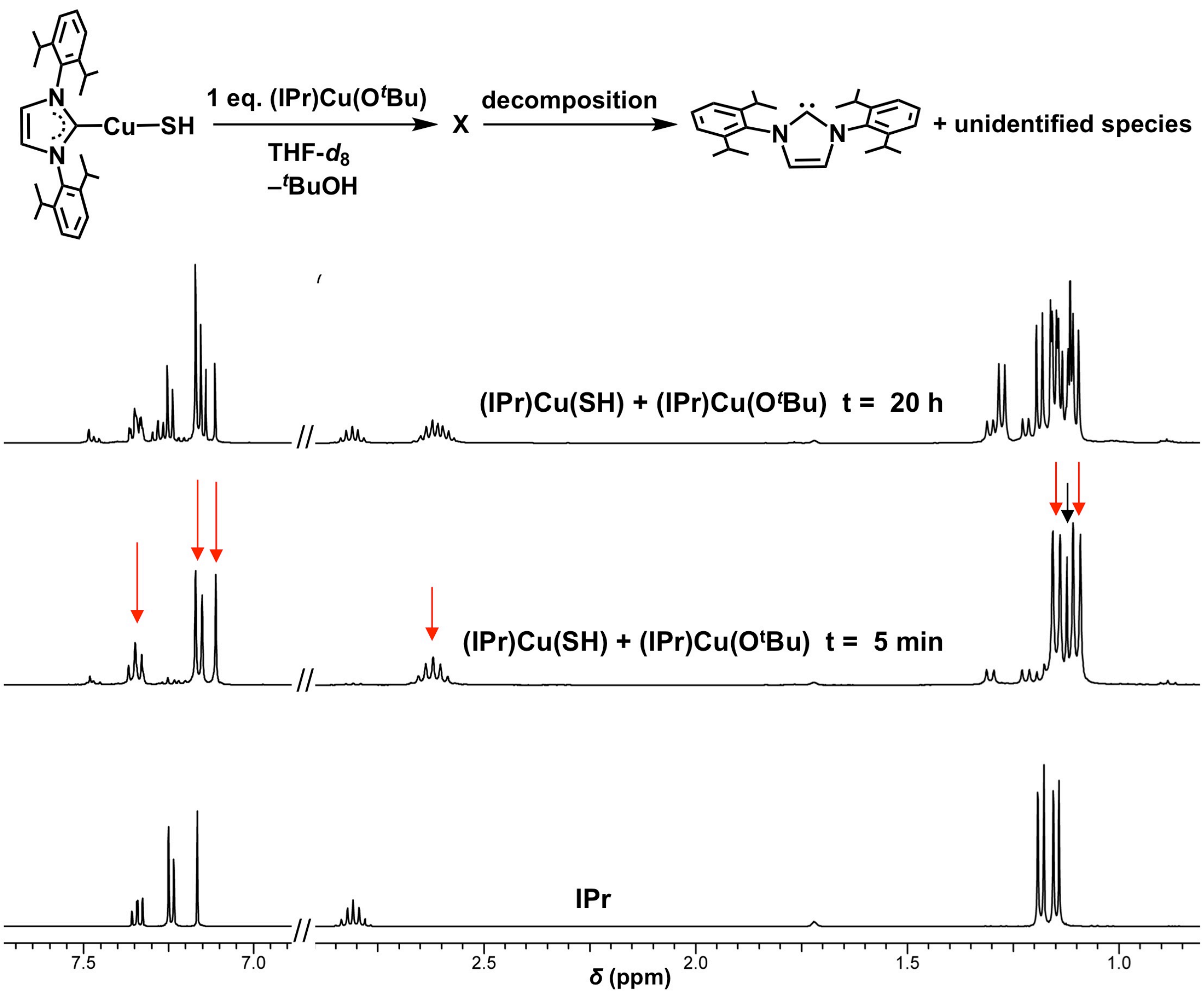

Figure S4. ^{1}H-NMR spectra of the reaction between (IPr)Cu(SH) and (IPr)Cu(O^{t}Bu) in THF-d_8 at t ≈ 5 min and 20 h. The ^{1}H-NMR spectrum of IPr in THF-d_8 is given at bottom as the reference. The resonances identified by red arrows are assigned to **X**. The resonance identified by the black arrow is assigned to ^{t}BuOH (δ 1.12, CH_3).

Synthesis of (IPr*)Cu(SBn) (8). Two 20 mL vials, one each containing a THF (5 mL) solution of **5** (0.254 g, 0.242 mmol) and a THF (1 mL) solution of $PhCH_2SH$ (BnSH) (0.030 g, 0.242 mmol), were cooled to –45 ºC in the glovebox refrigerator. After removing the vials from the refrigerator, the solution of BnSH was added via pipette to the solution of **5** with stirring. No obvious color change was observed. The light yellow solution was stirred at room temperature for 30 min and then evaporated to dryness under reduced pressure. The resulting white powder

was triturated with Et_2O (3 mL). The mixture was filtered through a glass frit. The white powder collected on the frit was washed with Et_2O (3mL) and pentane (3 mL) and dried under vacuum for 1 h to give 0.201 g of **8** (76% yield). ^{1}H NMR (22 ºC, 500 MHz, C_6D_6): δ 7.48 (d, 8H, $^3J_{HH}$ = 7.5 Hz, *o*-CH(C_6H_5)$_2$, 7.39 (d, 2H, $^3J_{HH}$ = 6.5 Hz, *o*-$SCH_2C_6H_5$), 7.20 (t, 8H, $^3J_{HH}$ = 7.5 Hz, *m*-CH(C_6H_5)$_2$), 7.06–6.93 (m, 31H, -$C_6H_2CH_3$, -CH(C_6H_5)$_2$ and *p*-, *m*-$SCH_2C_6H_5$), 5.64 (s, 4H, -C*H*Ph_2), 5.46 (s, 2H, -NC*H*=C*H*N-), 3.98 (s, 2H, -SC$H_2$$C_6H_5$), 1.72 (s, 6H, -$C_6H_2CH_3$). ^{13}C{^{1}H} NMR (23 ºC, 126 MHz, CD_2Cl_2): δ 182.1 (*C*Cu), 147.2, 143.3, 143.2, 141.5, 140.4, 134.8, 130.4, 130.1, 129.7, 128.9, 128.8, 128.5, 128.1, 127.0, 126.9, 125.2, 123.4, 51.73 (-*C*HPh_2), 29.45 (-S*C*$H_2C_6H_5$), 21.88 (-C_6H_2*C*H_3). Anal. Calcd. for ($C_{76}H_{63}CuN_2S$+0.5THF): C, 82.47%; H, 5.94%; N, 2.47%. Found: C, 82.12%; H, 6.35%; N, 2.26%.

Reaction between {(IPr*)Cu}$_2$(*μ*-S) and BnBr. To a C_6D_6 (~1 mL) solution of **1** (0.016 g, 0.008 mmol), a C_6D_6 (~1 mL) solution of $PhCH_2Br$ (BnBr) (0.001 g, 0.006 mmol) was added dropwise via pipette at room temperature. Hexamethylbenzene (0.001 g) was added as internal standard, and the solution was transferred to an NMR tube. The reaction was then monitored by ^{1}H-NMR spectroscopy (Figure S5). After ~1 h, the light yellow color of the solution faded and the spectrum showed that BnBr was completely consumed and that (IPr*)CuBr and **8** had formed in quantitative yield with a 1:1 molar ratio. A small quantity of unreacted **1** was also detected in a ~0.2:1 molar ratio to compound **8**. The identities of (IPr*)CuBr and **8** were confirmed by comparison to the ^{1}H-NMR spectra of authentic samples.

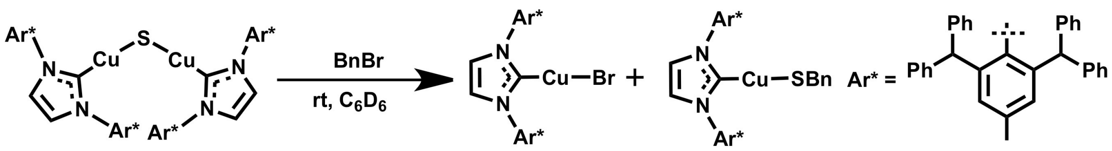

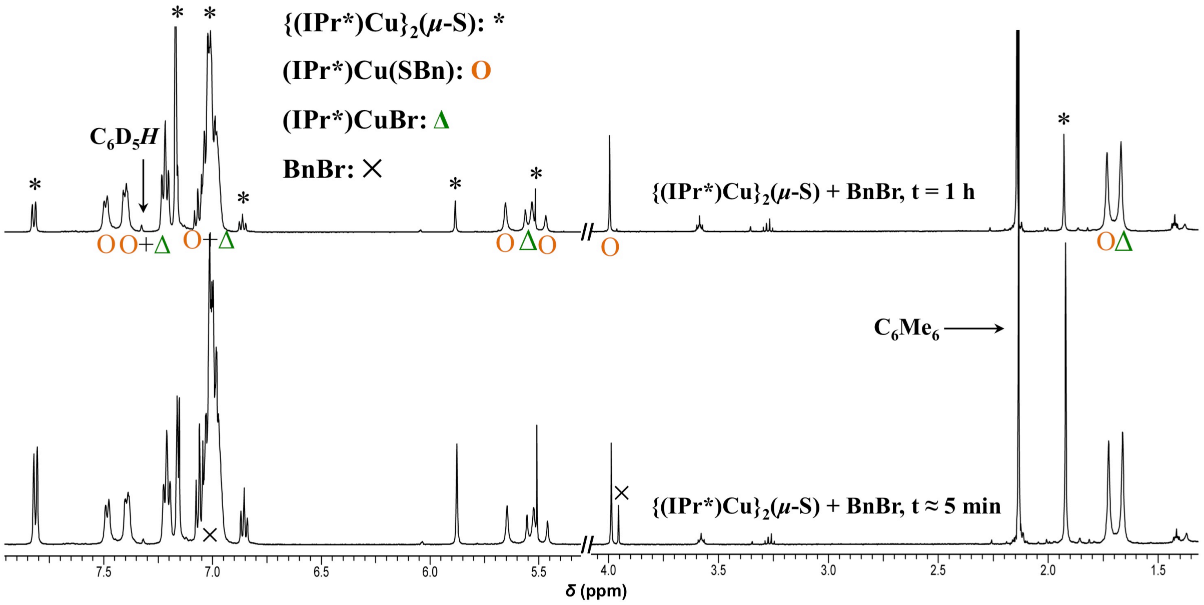

Figure S5 ^{1}H-NMR spectra of the reaction between {(IPr*)Cu}$_2$(μ-S) and BnBr in C_6D_6 at t ≈ 5 min and 1 h. Assignments of resonances to specific compounds are denoted as follows: {(IPr*)Cu}$_2$(μ-S), *; (IPr*)Cu(SBn), O; (IPr*)CuBr Δ; and BnBr, ×.

Reaction between {(IPr*)Cu}$_2$(μ-S) and BnCl. A Tol-d_8 (~1 mL) stock solution of $PhCH_2Cl$ (BnCl, 0.03 mL, 0.26 mmol) was prepared in a 20 mL vial. To a light yellow Tol-d_8 (~0.8 mL) solution containing 0.021 g of **1** (0.011mmol) and 0.0004 g of hexamethylbenzene (internal standard), 0.05 mL of BnCl/Tol-d_8 stock solution was added via syringe. The solution was transferred to a J. Young NMR tube and monitored by ^{1}H-NMR spectroscopy. At room temperature the reaction proceeded very slowly, taking about three days to reach completion. However, the reaction went to completion when heated at 50 ºC for ~12 h. The room temperature ^{1}H-NMR spectrum of the resulting colorless solution showed overlapping resonances that hindered the identification of the products. At low temperature (260 K), well separated proton

resonances of (IPr*)CuCl and **8** were identified by comparison to the spectra of independently synthesized samples (Figure S6). Based on the integration of IPr* p-$C_6H_2CH_3$ resonances relative to the internal standard hexamethylbenzene, the yields of (IPr*)CuCl and **8** were estimated to be approximately 96% and 78%, respectively.

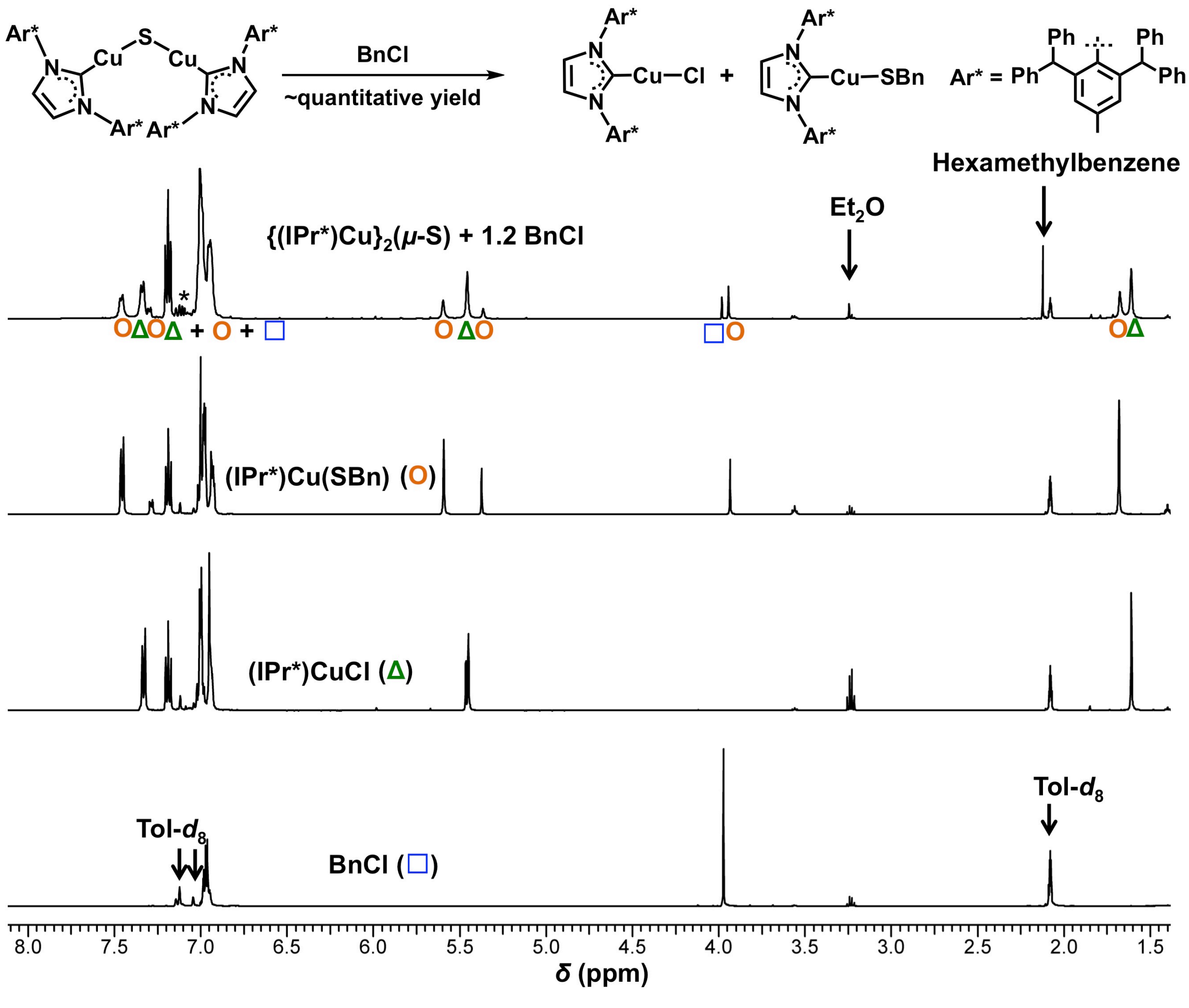

Figure S6 Top: ^{1}H-NMR spectrum of the product mixture from the reaction between **1** and BnCl. Middle and bottom: ^{1}H-NMR spectra of independently synthesized (IPr*)Cu(SBn) (**8**) and (IPr*)CuCl and commercial BnCl (Aldrich), respectively. All spectra were recorded at 260 K in Tol-d_8. Assignments of resonances to specific compounds are denoted as follows: (IPr*)CuCl, Δ; **8**, O; and BnCl, □. The unidentified species is labeled *.

Reaction between $\{(IPr^*)Cu\}_2(\mu\text{-}S)$ and 1,3-dibromopropane. To a C_6D_6 (~1 mL) solution of $Br(CH_2)_3Br$ (0.005 g, 0.025 mmol) containing 0.003 g of hexamethylbenzene as internal standard, 0.035 g of **1** (0.018 mmol) was added. The mixture was vigorously shaken to dissolve **1** and the resulting light yellow solution was quickly transferred to a NMR tube. Monitoring of the reaction by ^1H-NMR spectroscopy showed that **1** was consumed within 5 min, with generation of a reaction intermediate proposed as $(IPr^*)Cu\{S(CH_2)_3Br\}$ (Figure S7). This formulation is based on the ^1H-NMR spectrum of the reaction mixture, which exhibits triplets at 3.37 and 2.85 ppm and a quintet at 1.82 ppm with integration ratio ~1:1:1, consistent with the –$S(CH_2)_3Br$ group. In the course of 7 h the color of the reaction mixture faded from light yellow to colorless and $(IPr^*)Cu\{S(CH_2)_3Br\}$ was gradually consumed, with the concomitant formation of thietane and (IPr*)CuBr (Figure S7). The final yields of thietane and (IPr*)CuBr were estimated to be 84% and 77% respectively, as referred to hexamethylbenzene. The reaction mixture was then filtered through silica (contained in a pipette) and analyzed by GC-MS, which further confirmed the formation of thietane.

For $(IPr^*)Cu\{S(CH_2)_3Br\}$: ^1H NMR (22 ºC, 500 MHz, C_6D_6): δ 3.37 (t, 2H, $^3J_{HH}$ = 7.0 Hz, -SCH_2-), 2.85 (t, 2H, $^3J_{HH}$ = 7.0 Hz, -CH_2Br), 1.82 (quin, 2H, $^3J_{HH}$ = 6.5 Hz, -$SCH_2CH_2CH_2Br$). The IPr* resonances of the compound could not be identified at room temperature due to their overlap with (IPr*)CuBr resonances.

For thietane: ^1H NMR (22 ºC, 500 MHz, C_6D_6): δ 2.82 (t, 4H, $^3J_{HH}$ = 8.0 Hz, -SCH_2-), 2.37 (m, 2H, -$CH_2CH_2CH_2$-). GC-MS (m/z): 73.9 (M^+; calculated, 74.0), 46.0.

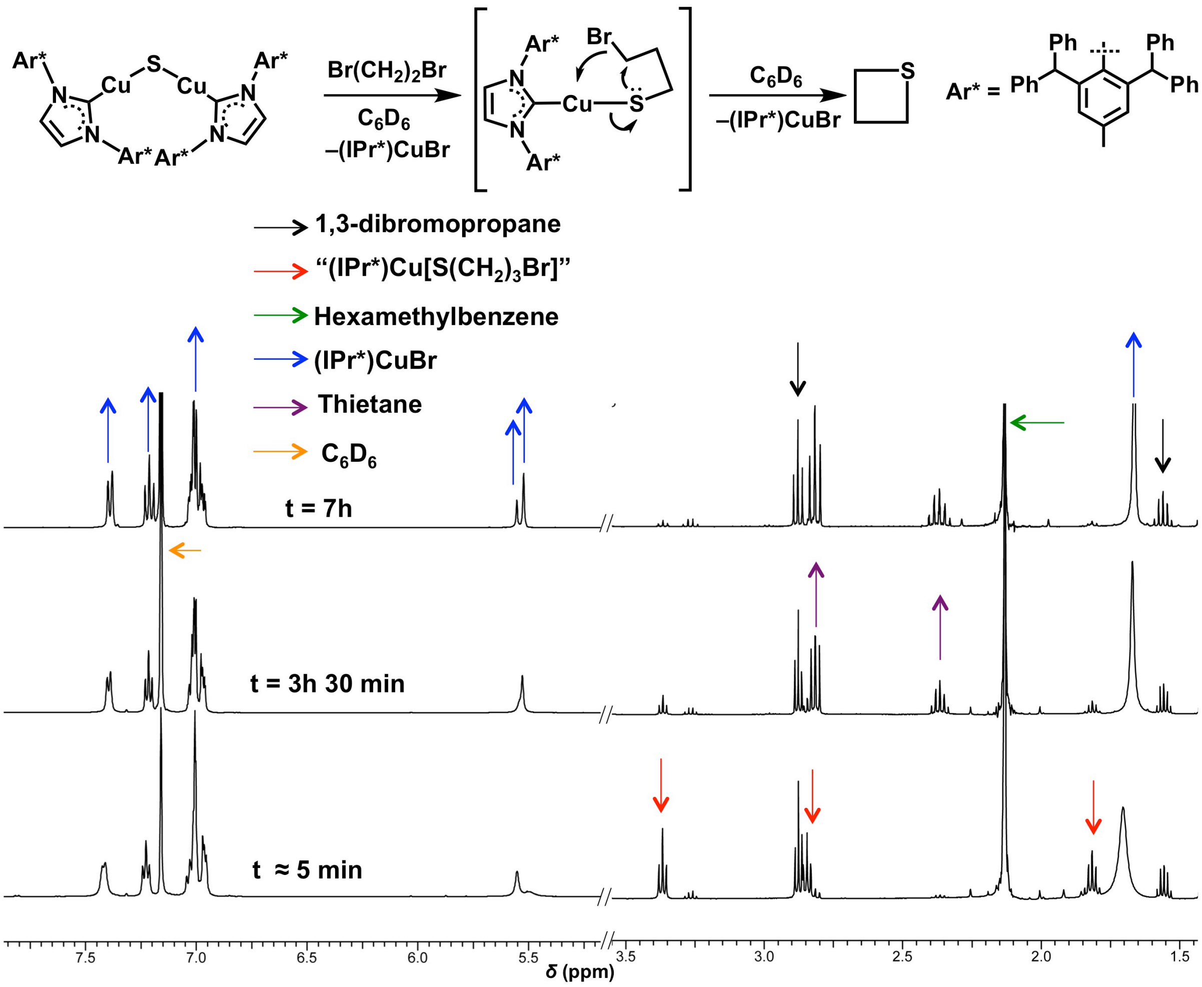

Figure S7. ^{1}H-NMR spectra of the reaction of **1** with 1,3-dibromopropane in C_6D_6. Bottom: t ≈ 5 min; Middle: t = 3.5 h; Top: t = 7 h

Reaction between {(IPr*)Cu}$_2$(*μ*-S) and 1,4-dibromobutane. The reaction was carried out following the procedure described above for the *Reaction between {(IPr*)Cu}$_2$(μ-S) and 1,3-dibromopropane*, with $Br(CH_2)_4Br$ (0.005 g, 0.023 mmol), **1** (0.031 g, 0.016 mmol) and hexamethylbenzene (0.007 g, 0.04 mmol) in C_6D_6 (~1 mL). The reaction was complete within 1 h, during which time the solution turned from light yellow to colorless. Tetrahydrothiophene and (IPr*)CuBr were formed quantitatively (Figure S8). No reaction intermediates were observed in the ^{1}H-NMR spectrum recorded ~5 min after the mixing of **1** and $Br(CH_2)_4Br$ in C_6D_6.

For tetrahydrothiophene: ^{1}H NMR (22 ºC, 500 MHz, C_6D_6): δ 2.54 (m, 4H, -SCH_2-), 1.46 (m, 4H, -CH_2C$H_2$$CH_2$-). GC-MS (m/z): 88.0 ($M^+$; calculated, 88.0), 59.0, 45.0.

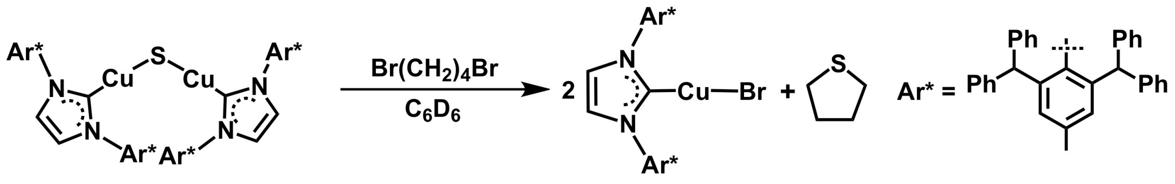

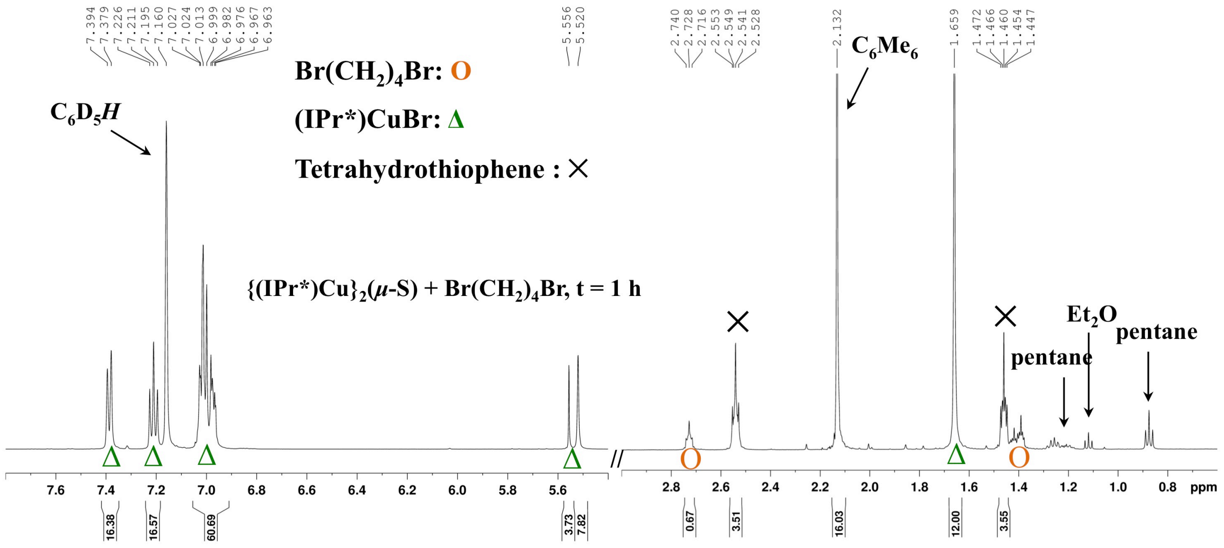

Figure S8. ^{1}H-NMR spectrum of the reaction of **1** with 1,4-dibromobutane in C_6D_6 at t = 1 h. Assignments of resonances to specific compounds are denoted as follows: Br$(CH_2)_4$Br, O; (IPr*)CuBr, Δ; tetrahydrothiophene, ×.

Reaction between {(IPr*)Cu}$_2$(*μ*-S) and 1,5-dibromopentane. The reaction was carried out following the procedure described above for the *Reaction between {(IPr*)Cu}$_2$(μ-S) and 1,3-dibromopropane*, with Br$(CH_2)_5$Br (0.005 g, 0.022 mmol), **1** (0.041 g, 0.021 mmol) and hexamethylbenzene (0.007 g, 0.04 mmol) in C_6D_6 (~1 mL). The reaction was complete within 1 h, during which time the light yellow color of the solution faded to colorless. Tetrahydrothiopyran and (IPr*)CuBr were formed quantitatively (Figure S9). No reaction intermediates were observed in the ^{1}H-NMR spectrum recorded ~5 min after the mixing of **1** and Br$(CH_2)_5$Br in C_6D_6.

For tetrahydrothiopyran: ^{1}H NMR (22 ºC, 500 MHz, C_6D_6): δ 2.33 (m, 4H, -SCH_2-), 1.54 (m, 4H, -SCH_2C$H_2$$CH_2$-), 1.22 (m, 2H, -$SCH_2CH_2CH_2$-). GC-MS (m/z): 101.8 ($M^+$; calculated, 102.1), 86.9, 67.0, 45.0.

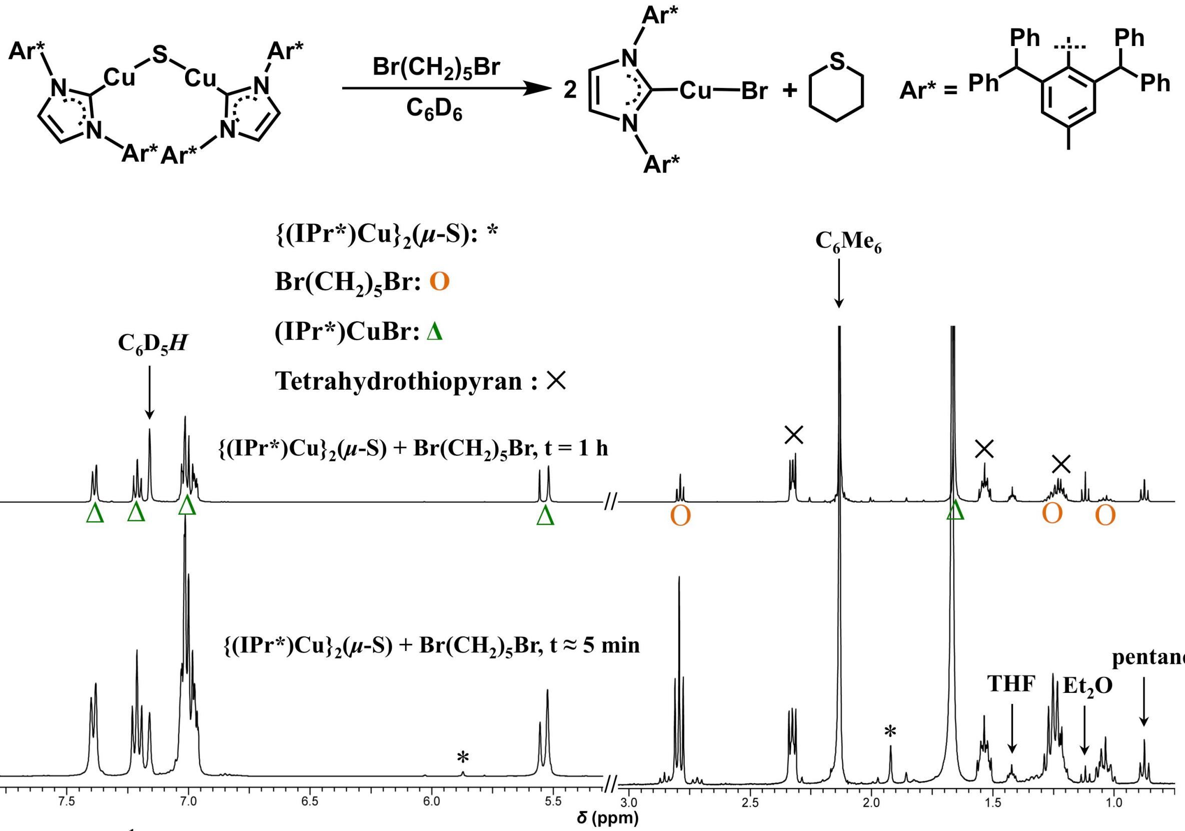

Figure S9. ^{1}H-NMR spectrum of the reaction of **1** with 1,5-dibromopentane in C_6D_6 at t ≈ 5 min and 1 h. Assignments of resonances to specific compounds are denoted as follows: **1**, *; Br$(CH_2)_5$Br, O; (IPr*)CuBr, Δ; and tetrahydrothiopyran, ×.

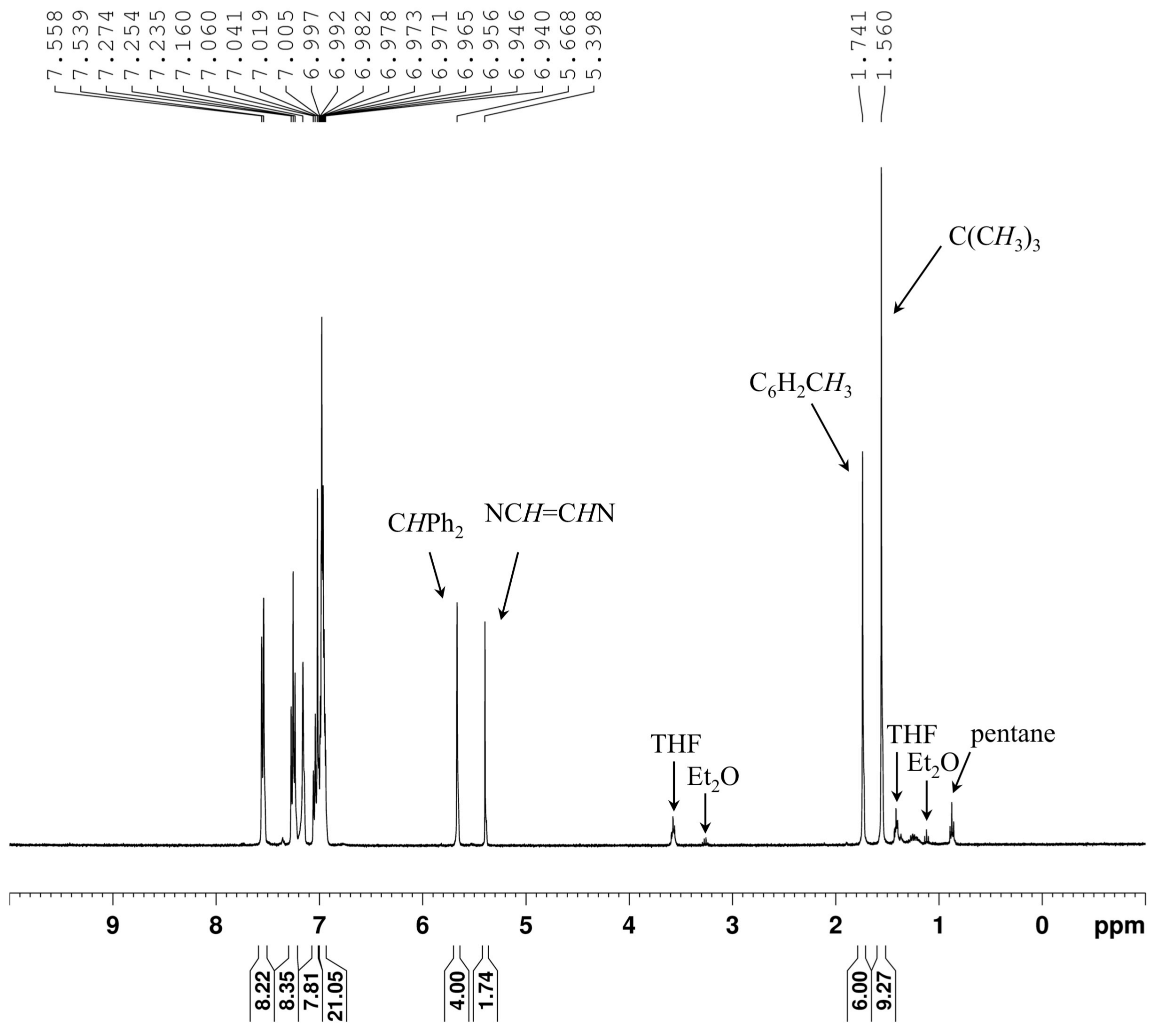

Figure S10. ^{1}H-NMR spectrum of compound **5** in C_6D_6.

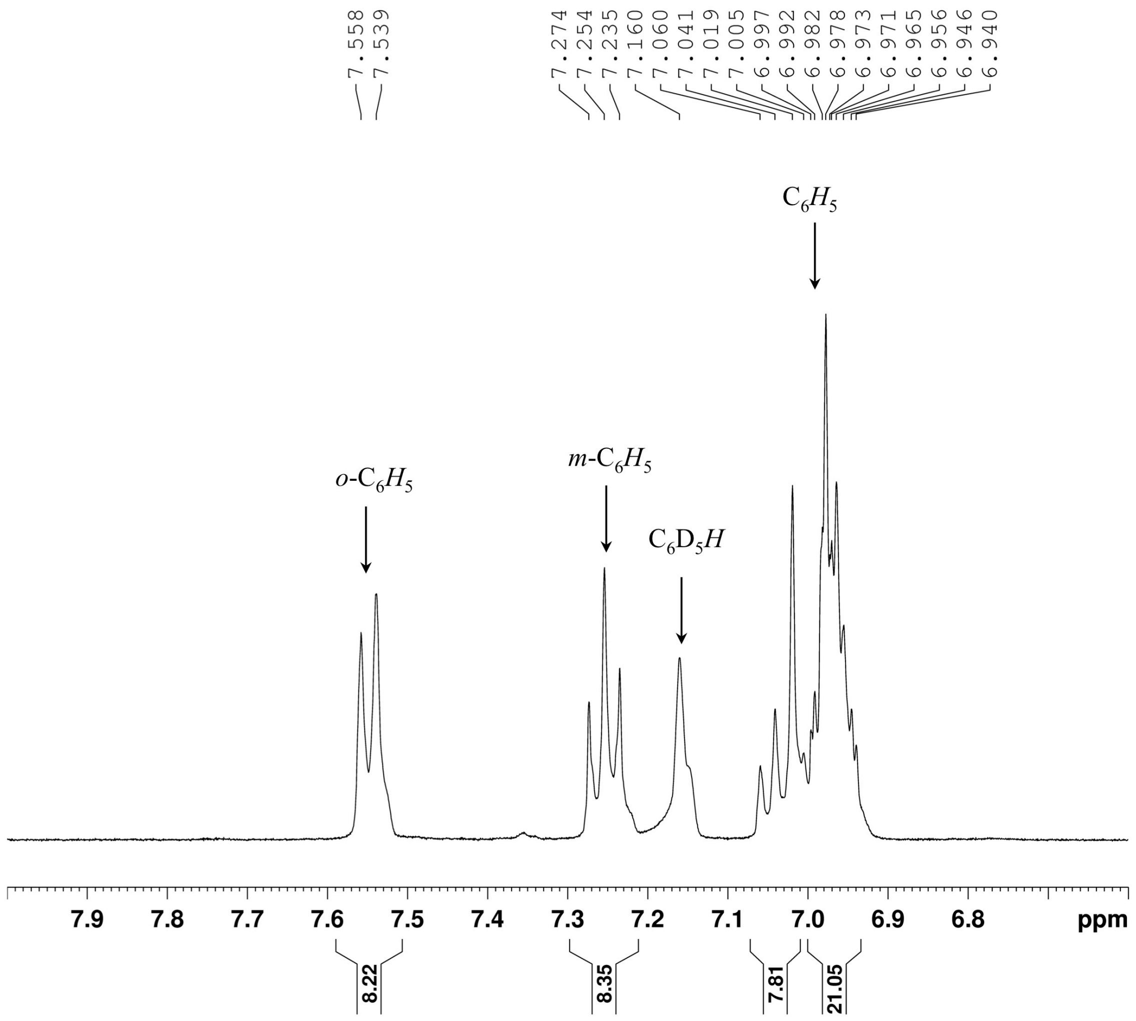

Figure S11. Expanded ^{1}H-NMR spectrum of compound **5** in C_6D_6.

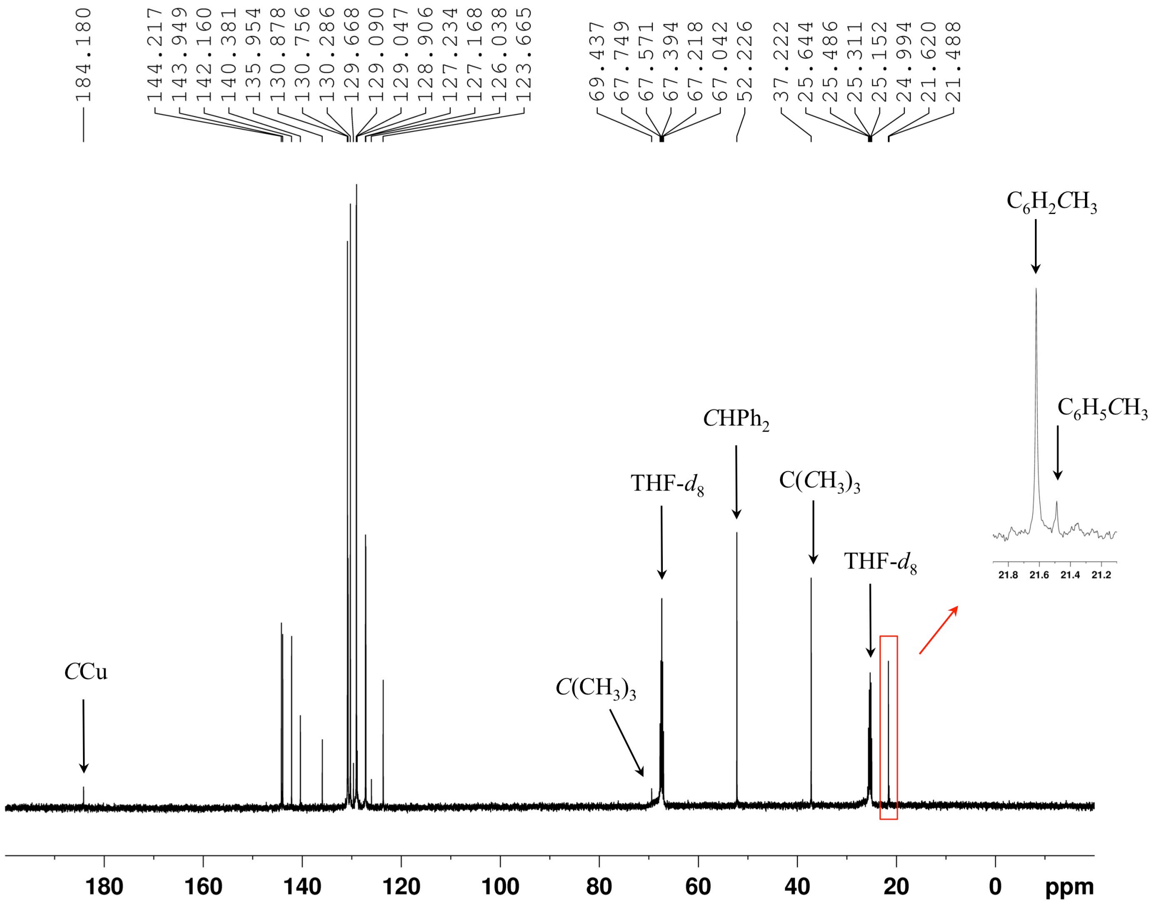

Figure S12. $^{13}C\{^1H\}$-NMR spectrum of compound **5** in THF-d_8.

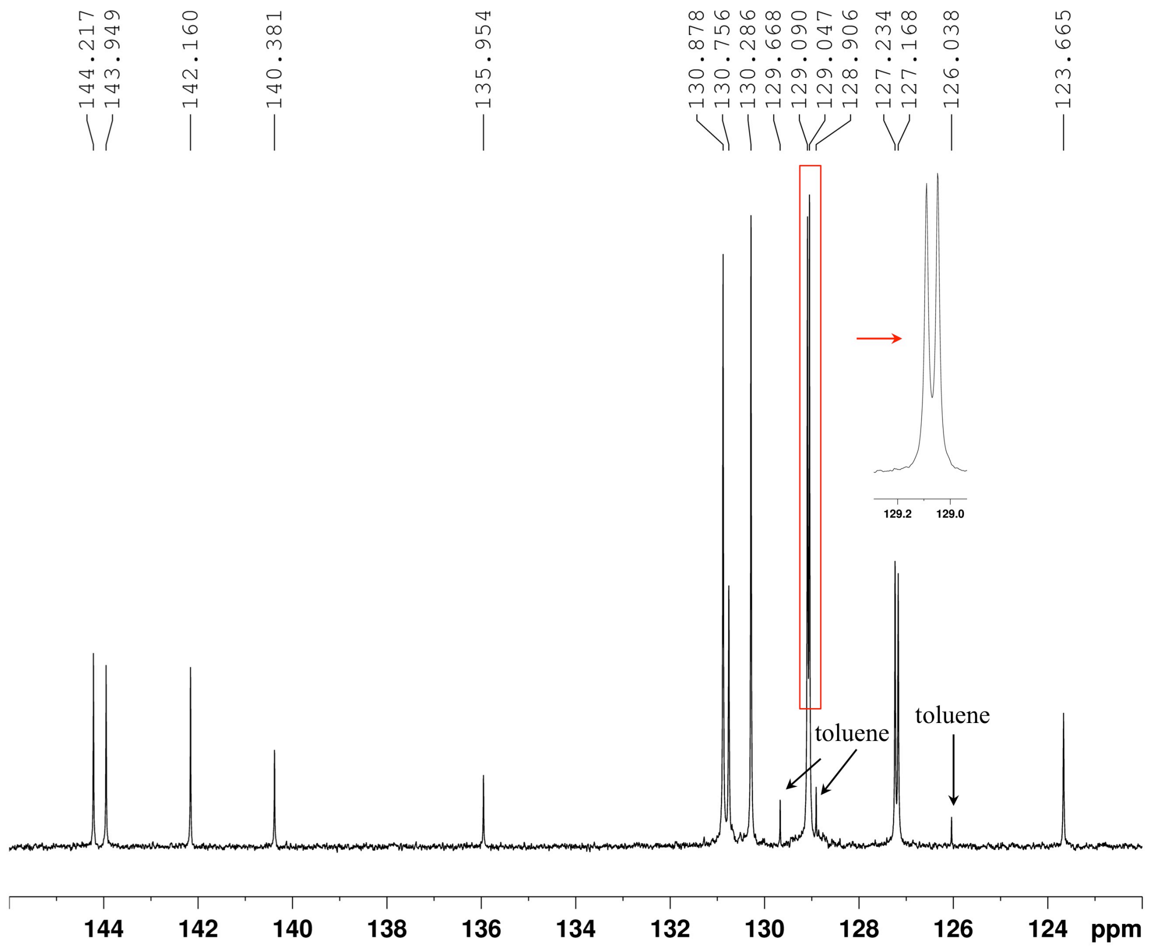

Figure S13. Expanded $^{13}C\{^{1}H\}$-NMR spectrum of compound **5** in THF-d_8.

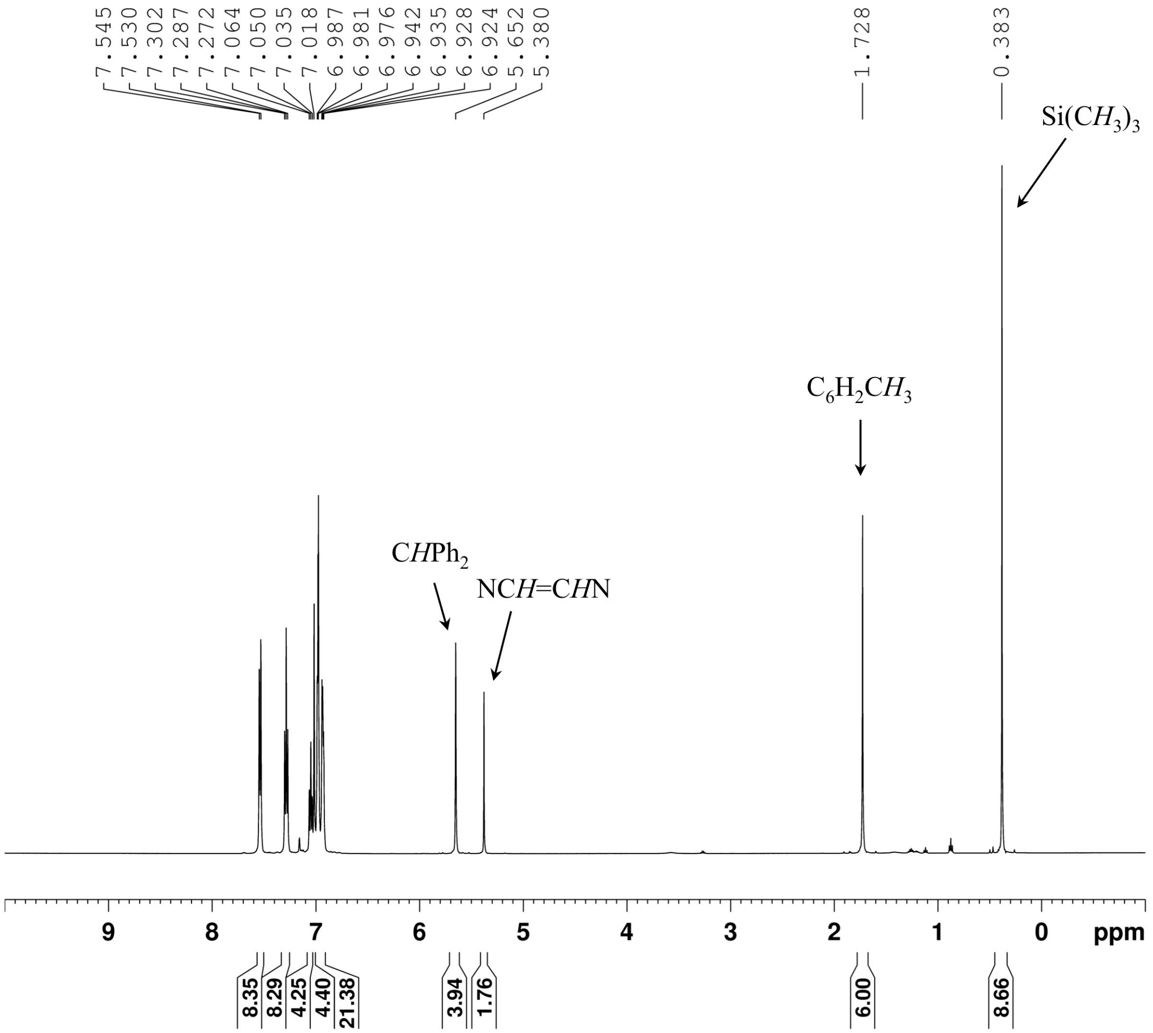

Figure S14. ^{1}H-NMR spectrum of compound **3** in C_6D_6.

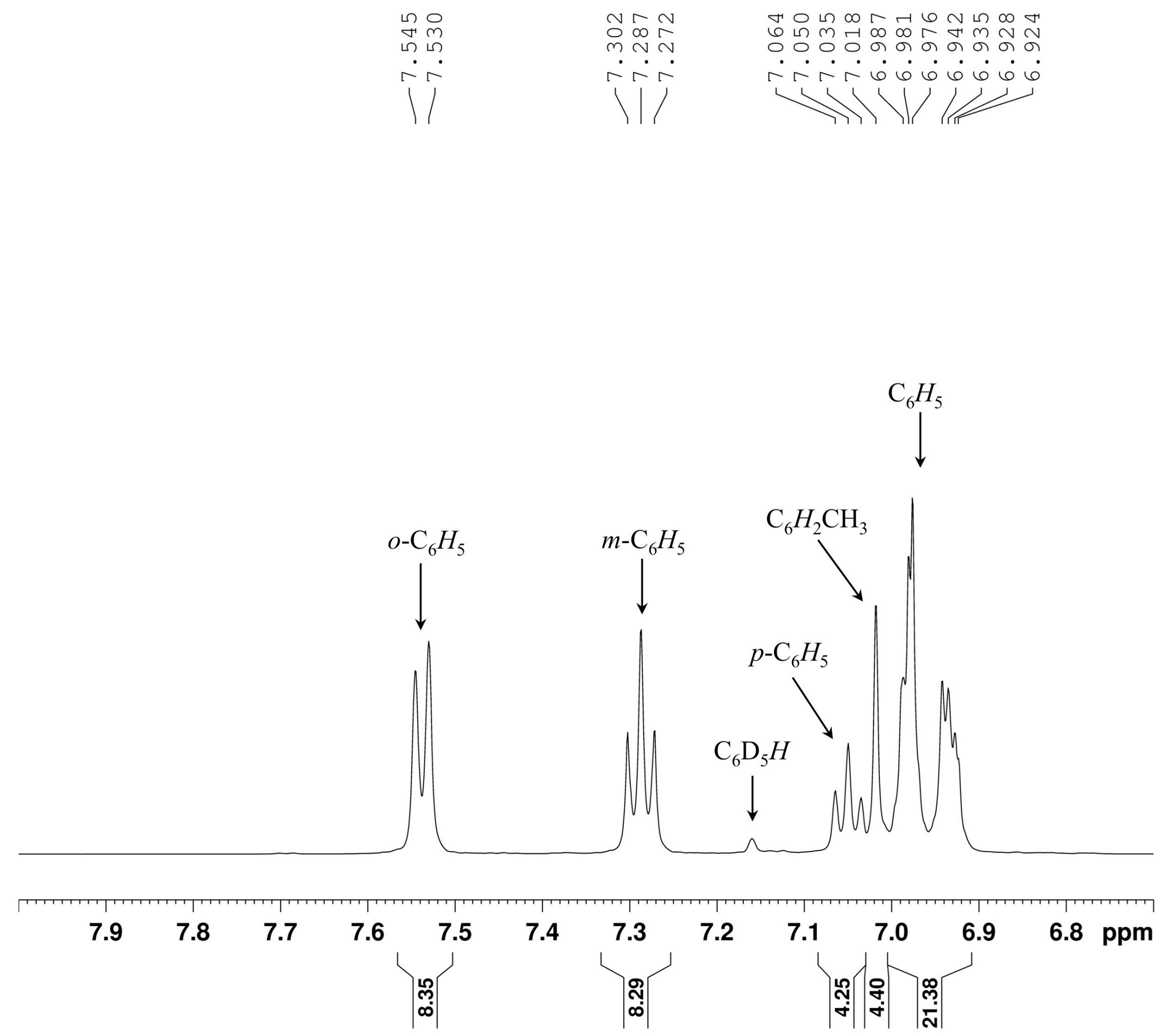

Figure S15. Expanded ^{1}H-NMR spectrum of compound **3** in C_6D_6.

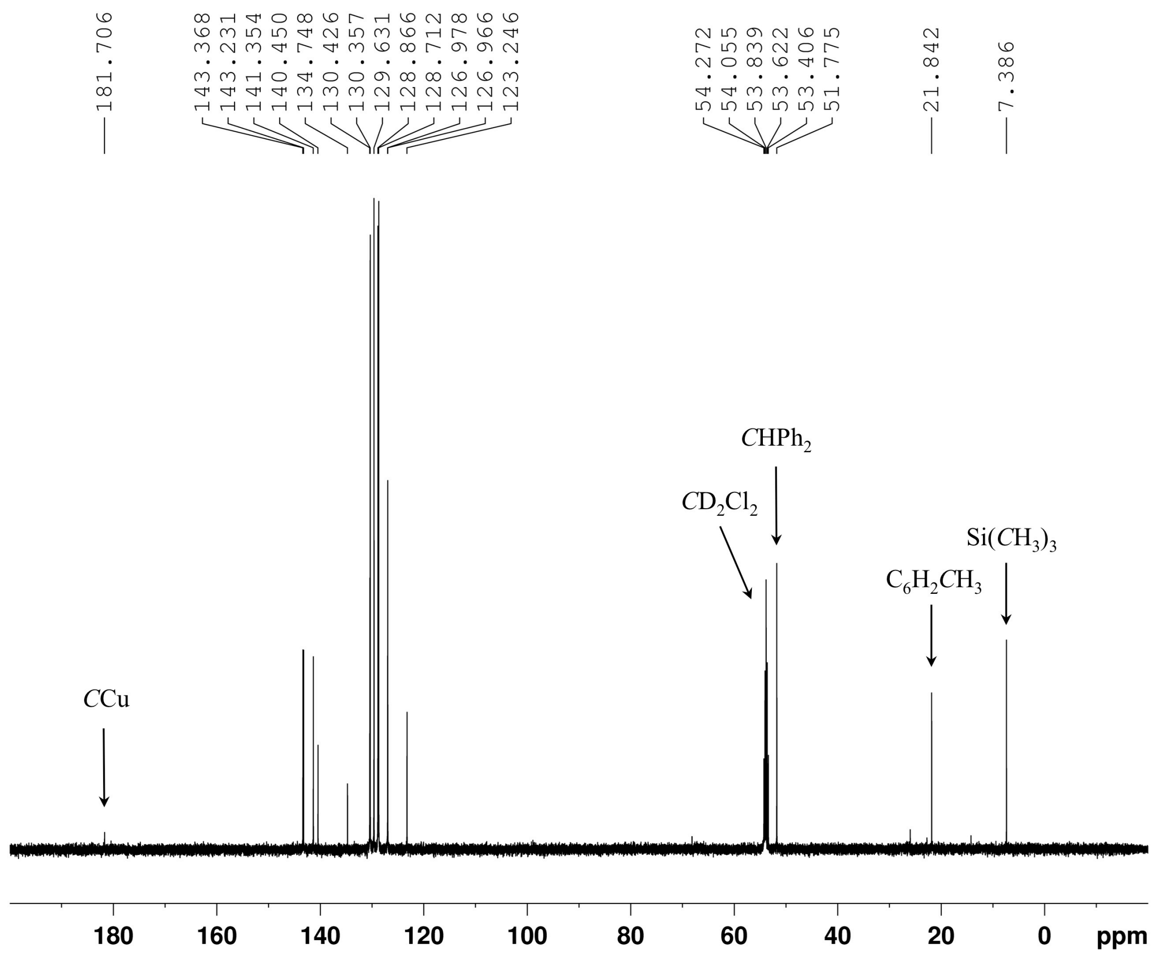

Figure S16. $^{13}C\{^1H\}$-NMR spectrum of compound **3** in CD_2Cl_2.

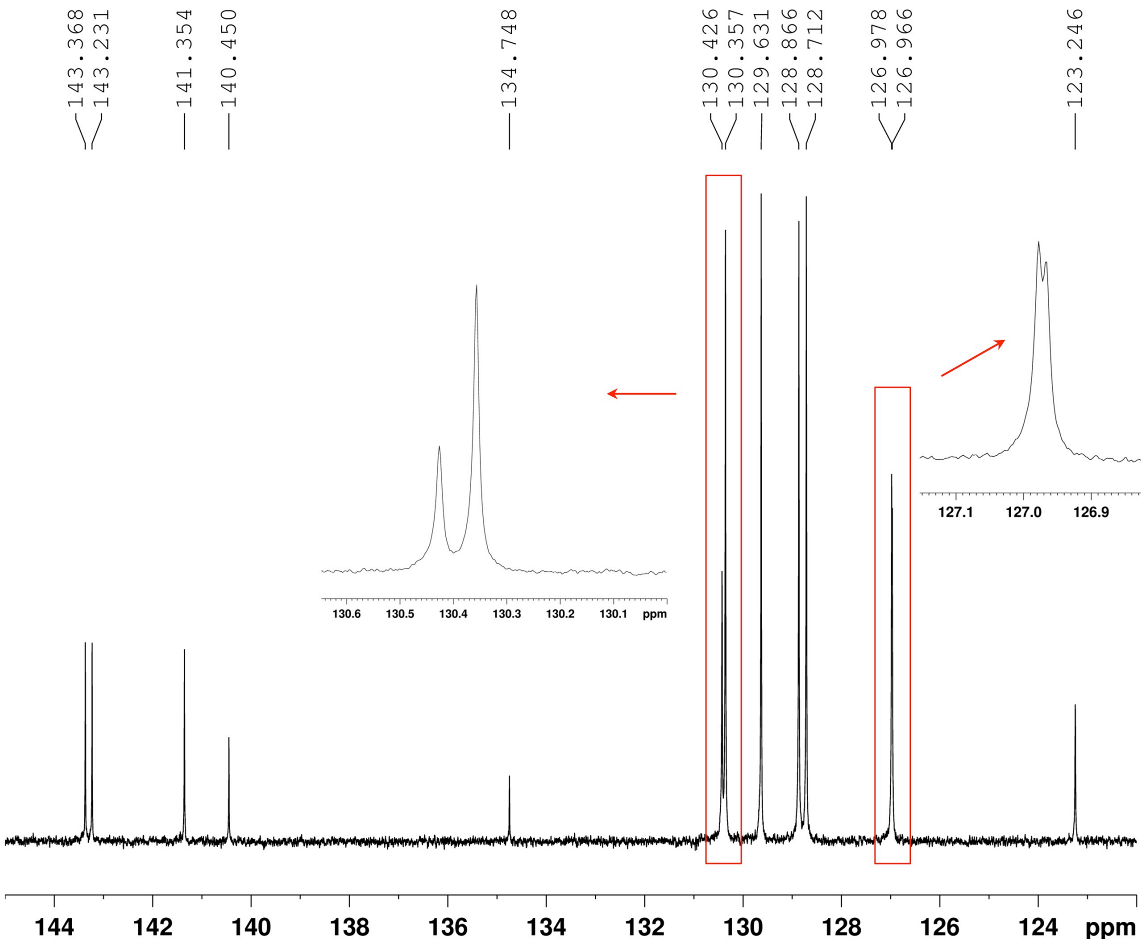

Figure S17. Expanded $^{13}C\{^1H\}$-NMR spectrum of compound **3** in CD_2Cl_2.

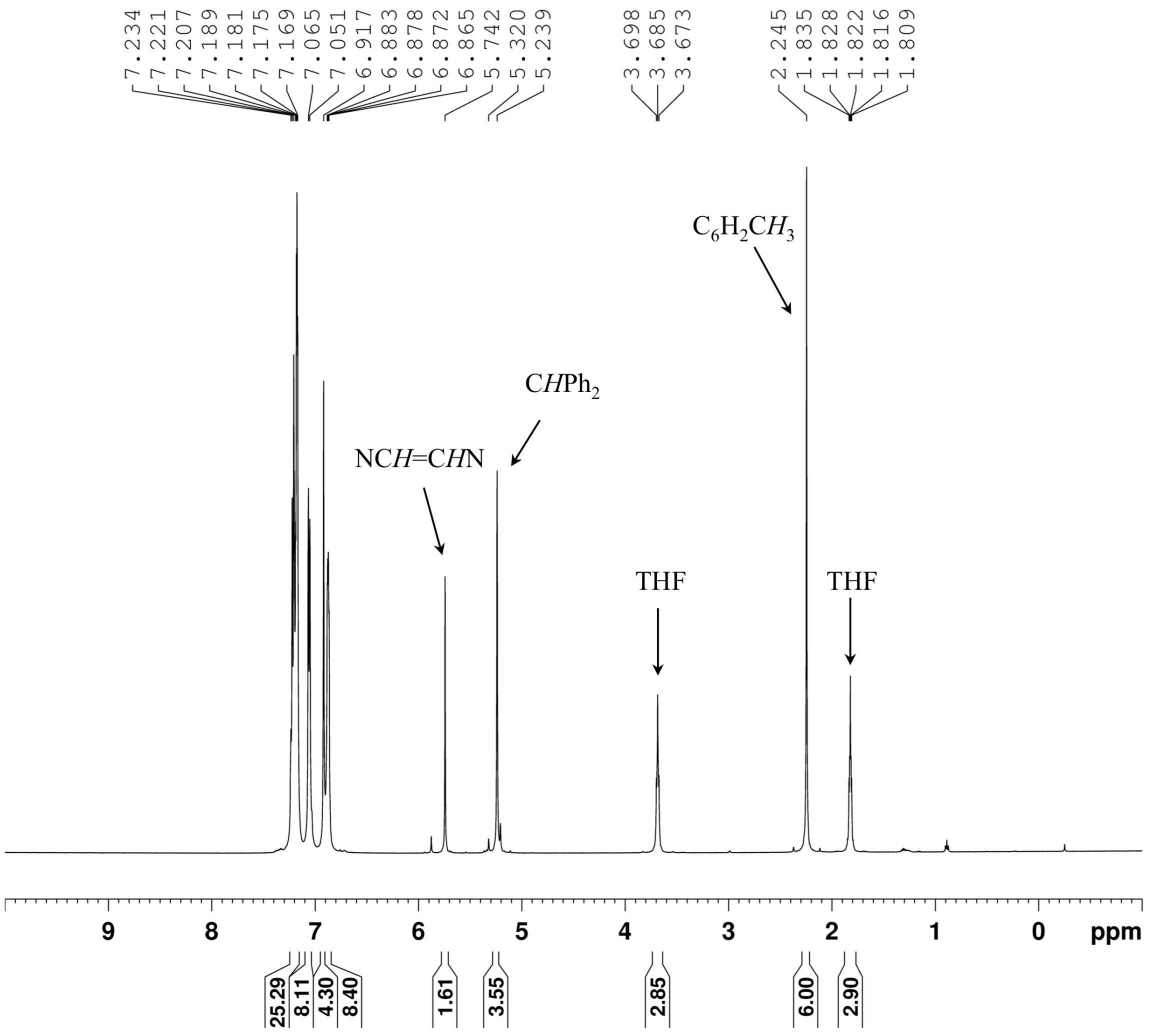

Figure S18. ^{1}H-NMR spectrum of compound **4** in CD_2Cl_2.

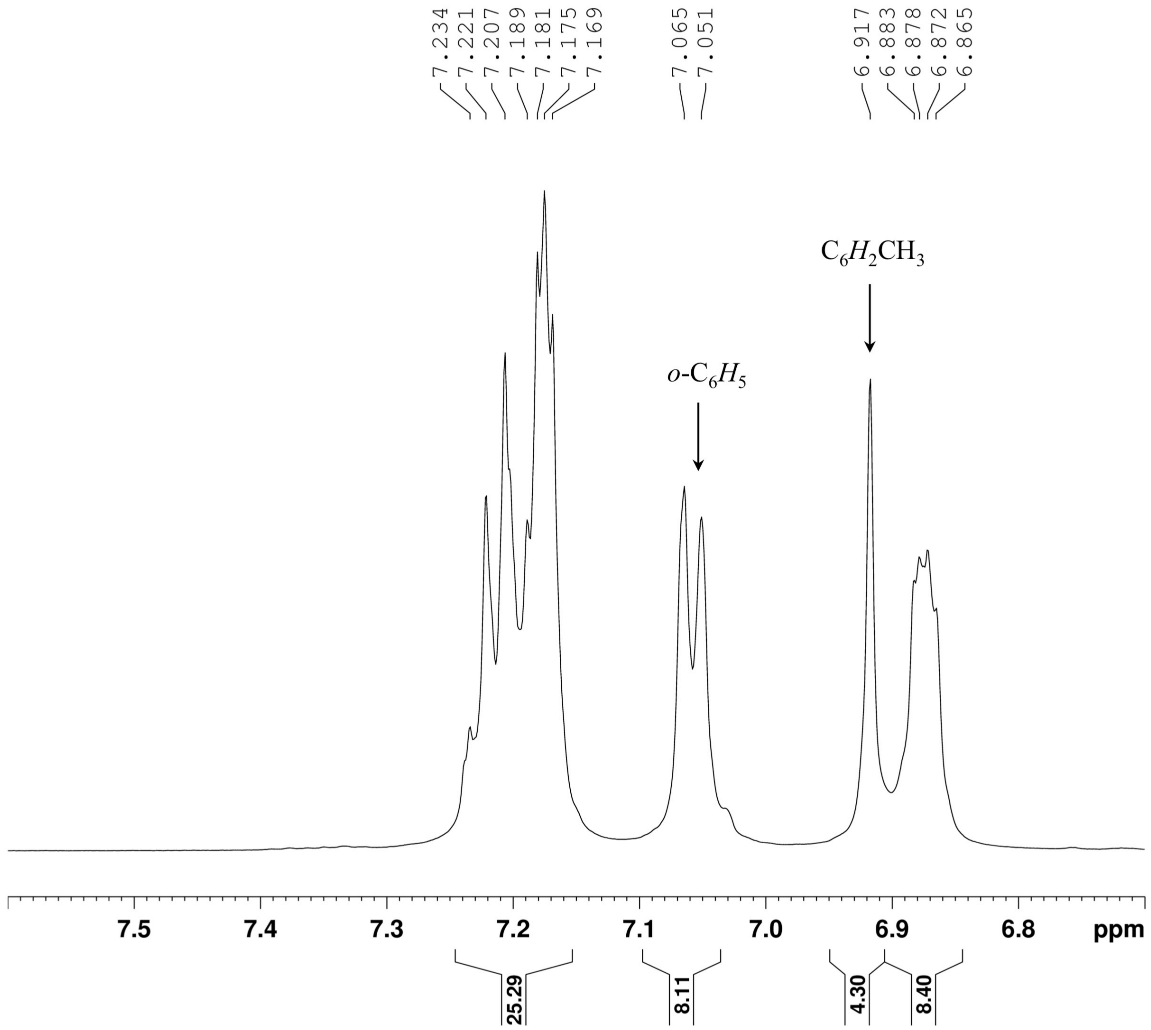

Figure S19. Expanded ^{1}H-NMR spectrum of compound **4** in CD_2Cl_2.

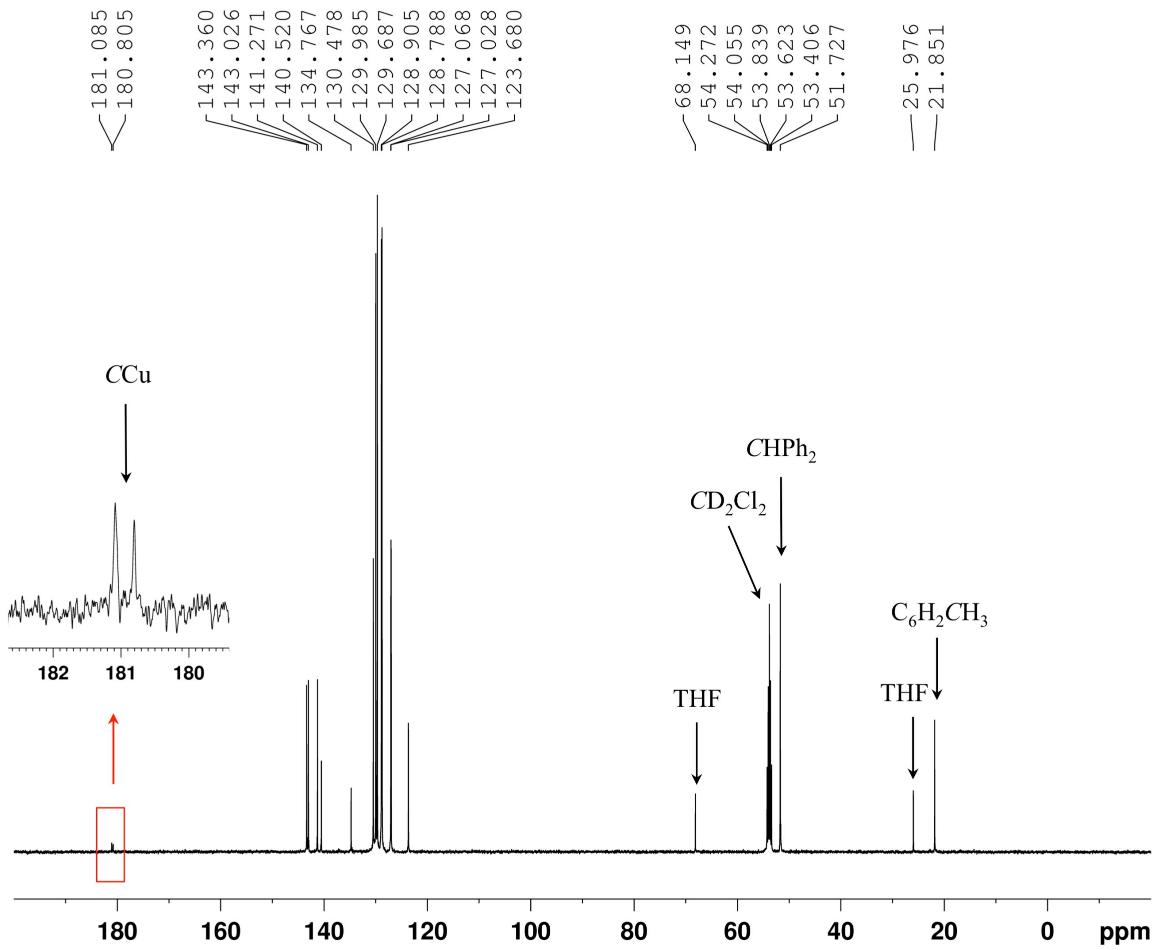

Figure S20. $^{13}C\{^{1}H\}$-NMR spectrum of compound **4** in CD_2Cl_2.

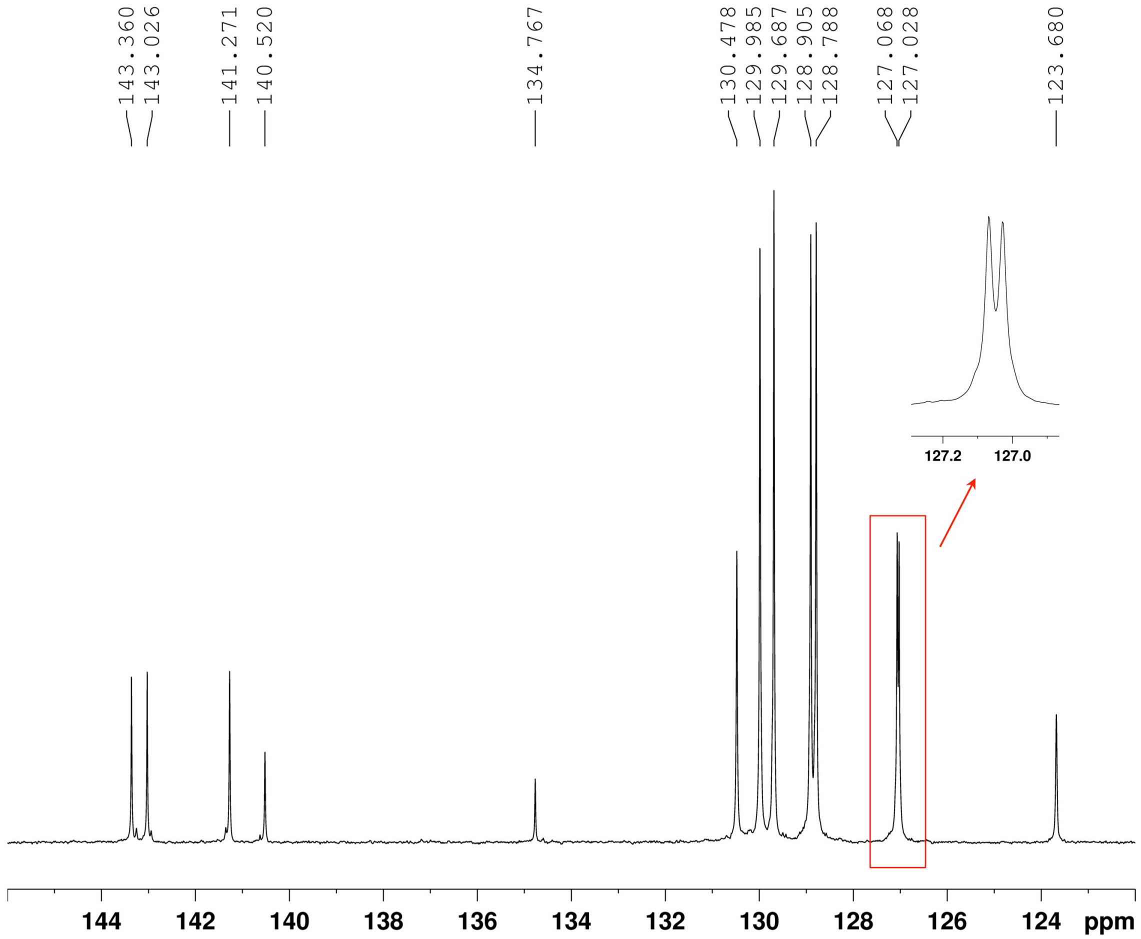

Figure S21. Expanded $^{13}C\{^{1}H\}$-NMR spectrum of compound **4** in CD_2Cl_2.

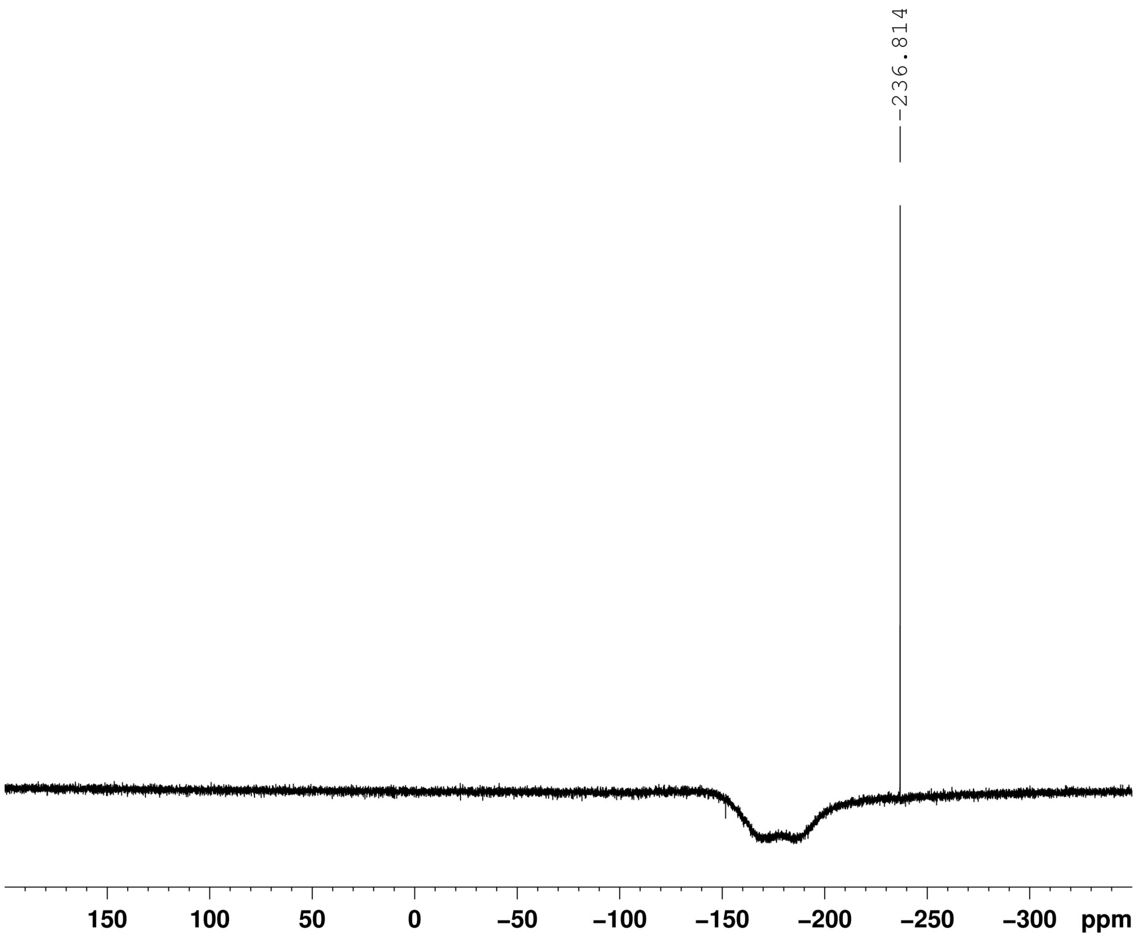

Figure S22. ^{19}F-NMR spectrum of compound **4** in CD_2Cl_2.

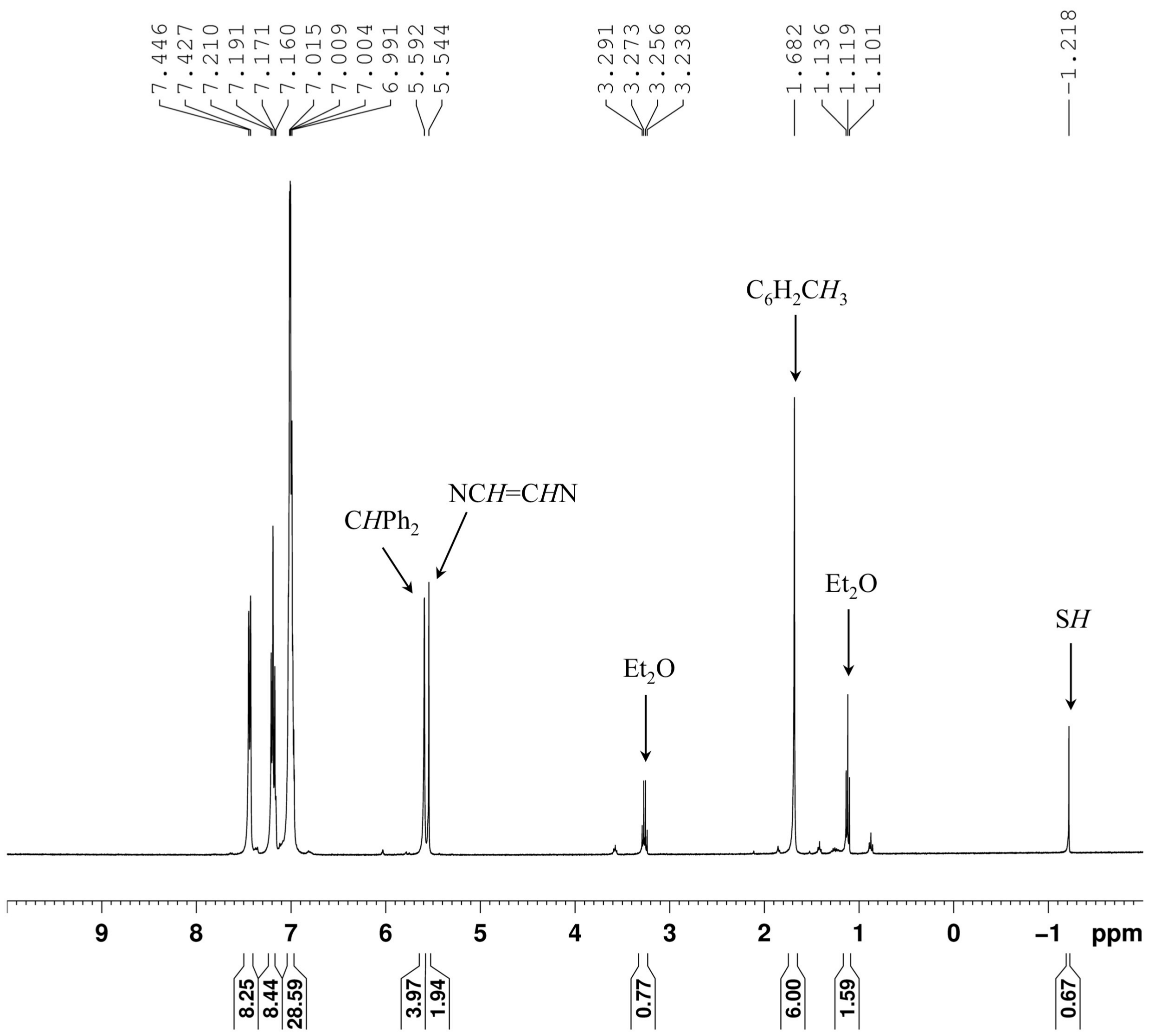

Figure S23. ^{1}H-NMR spectrum of compound **6** in C_6D_6.

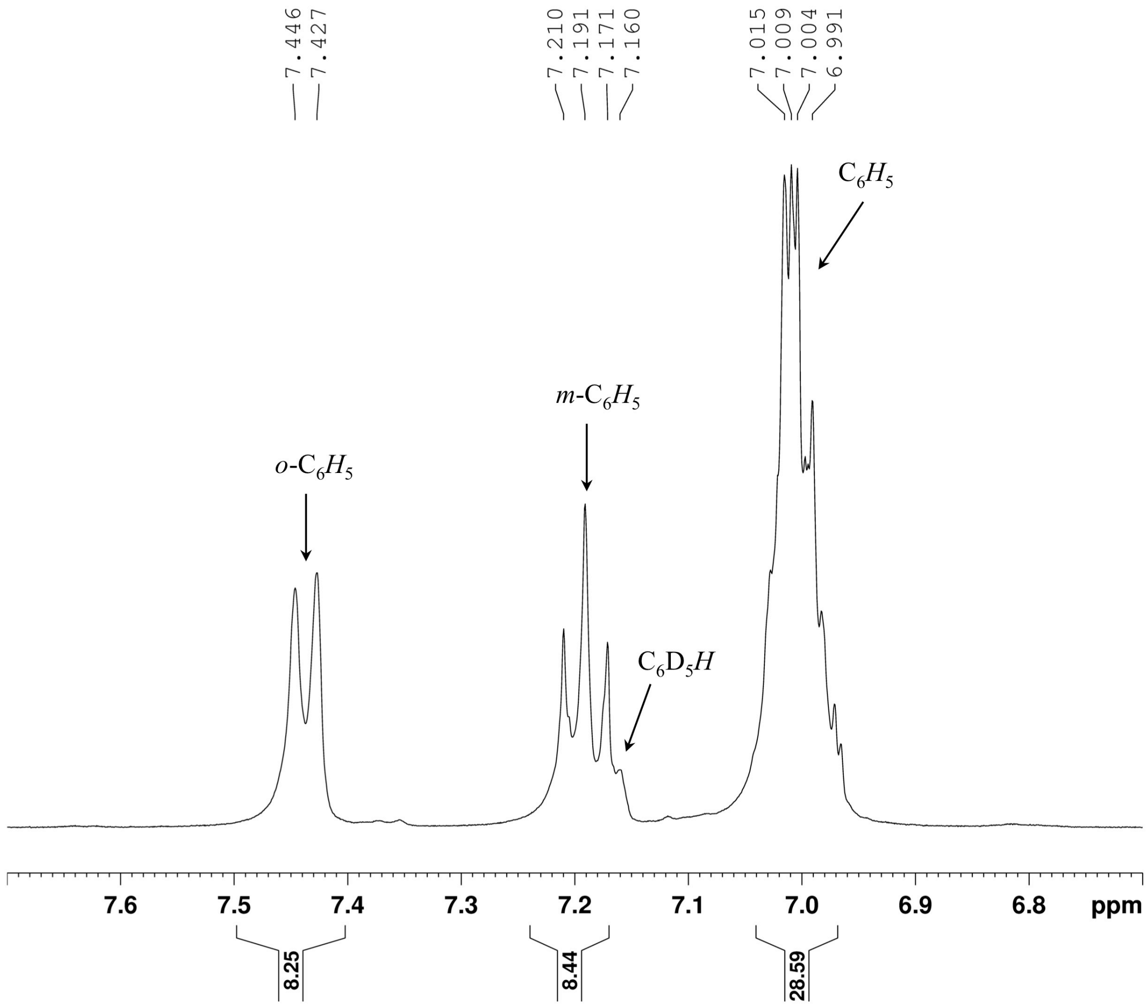

Figure S24. Expanded ^{1}H-NMR spectrum of compound **6** in C_6D_6.

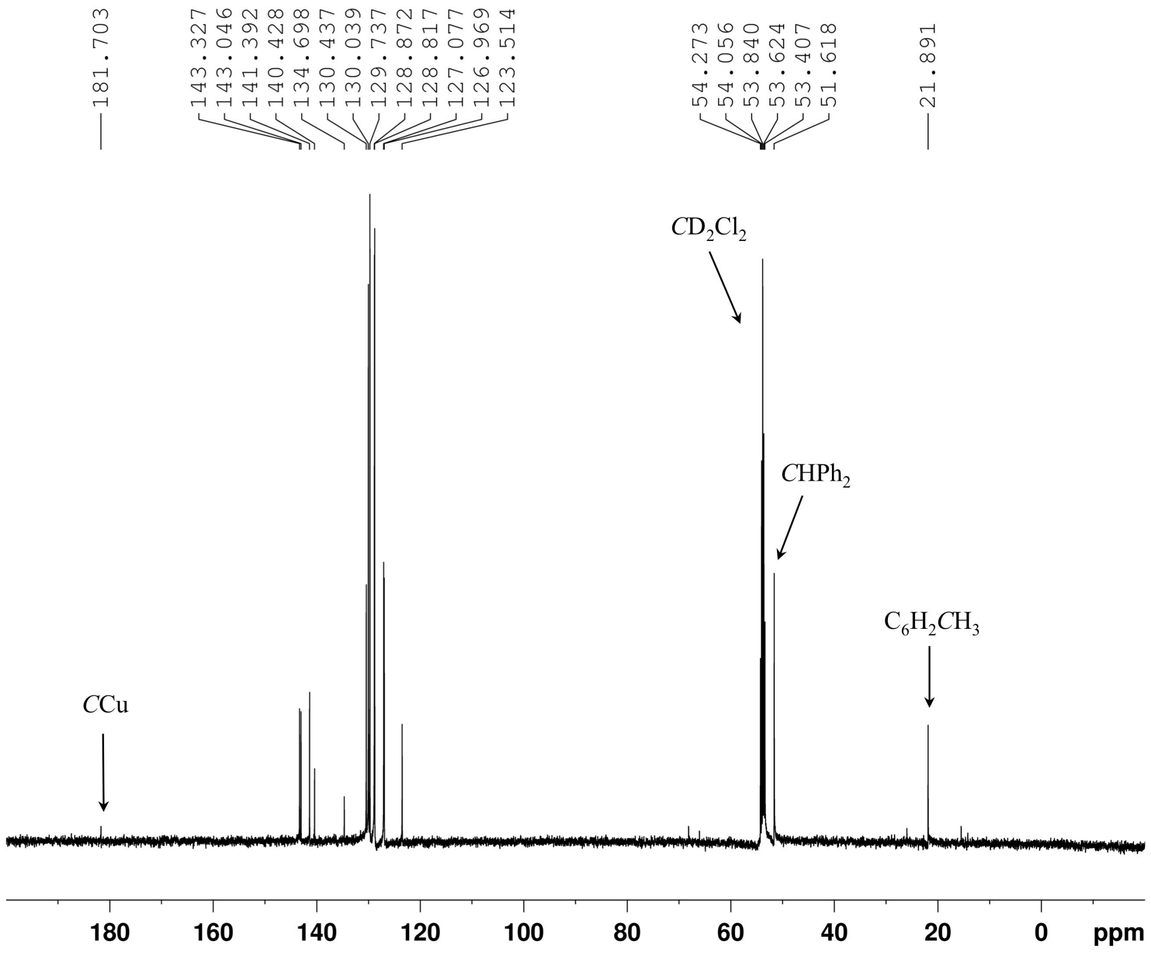

Figure S25. $^{13}C\{^1H\}$-NMR spectrum of compound **6** in CD_2Cl_2.

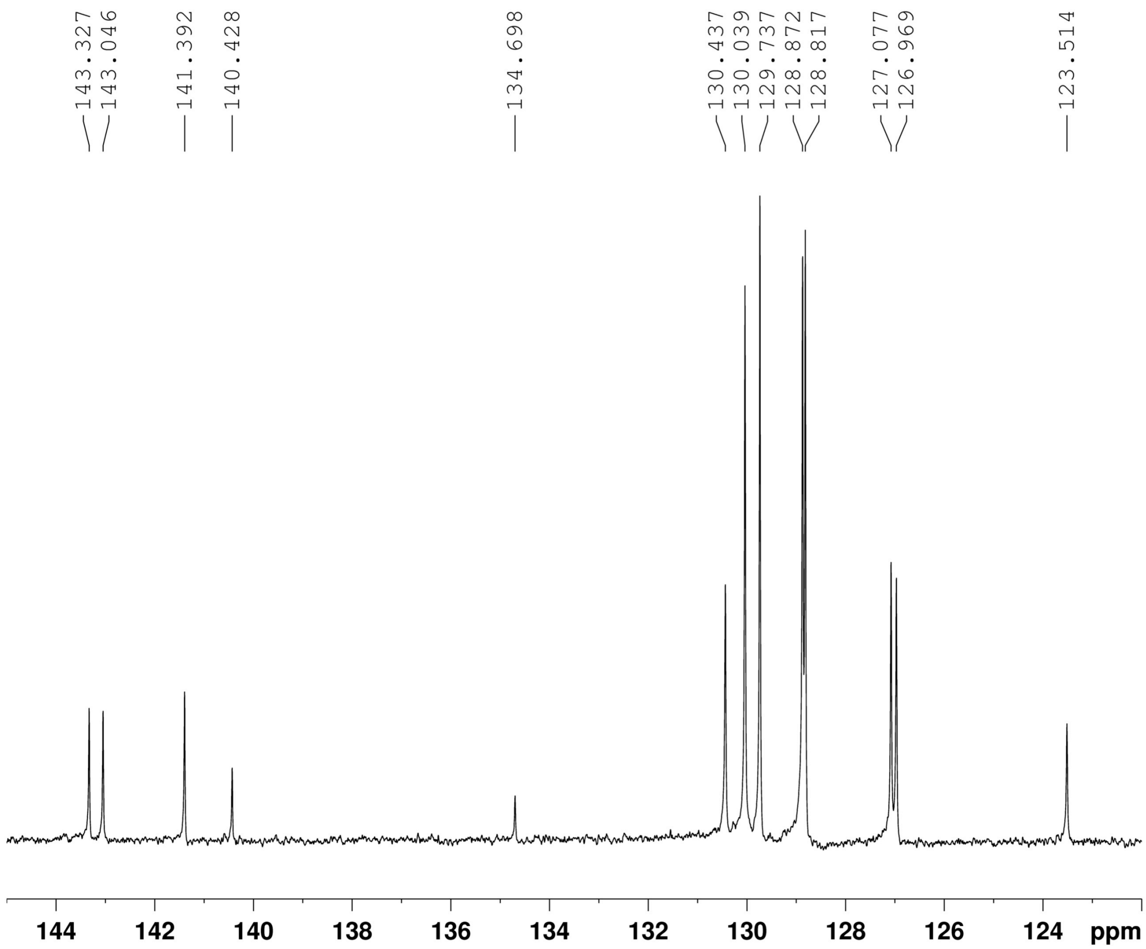

Figure S26. Expanded $^{13}C\{^1H\}$-NMR spectrum of compound **6** in CD_2Cl_2.

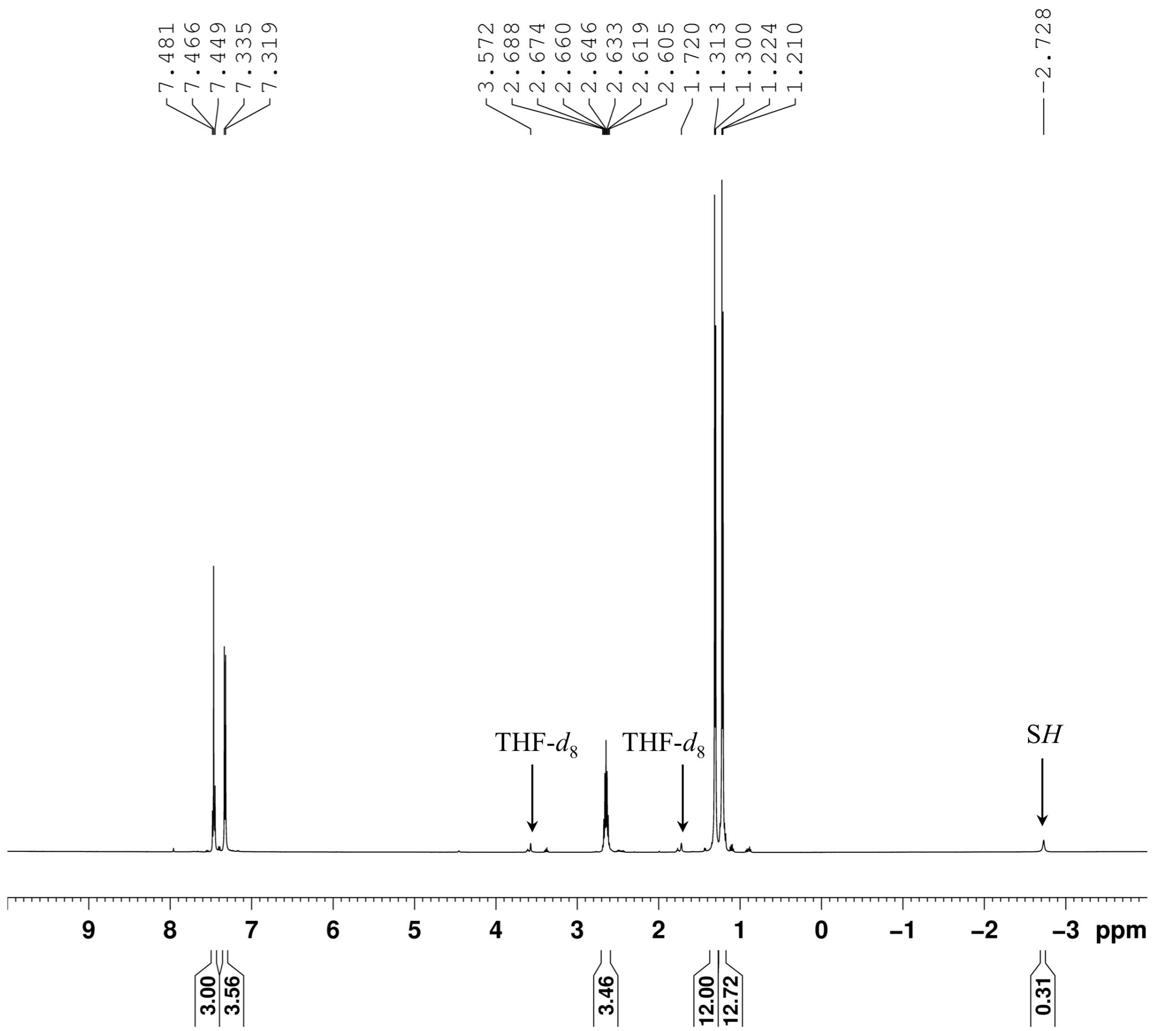

Figure S27. ^{1}H-NMR spectrum of compound **7** in THF-d_8.

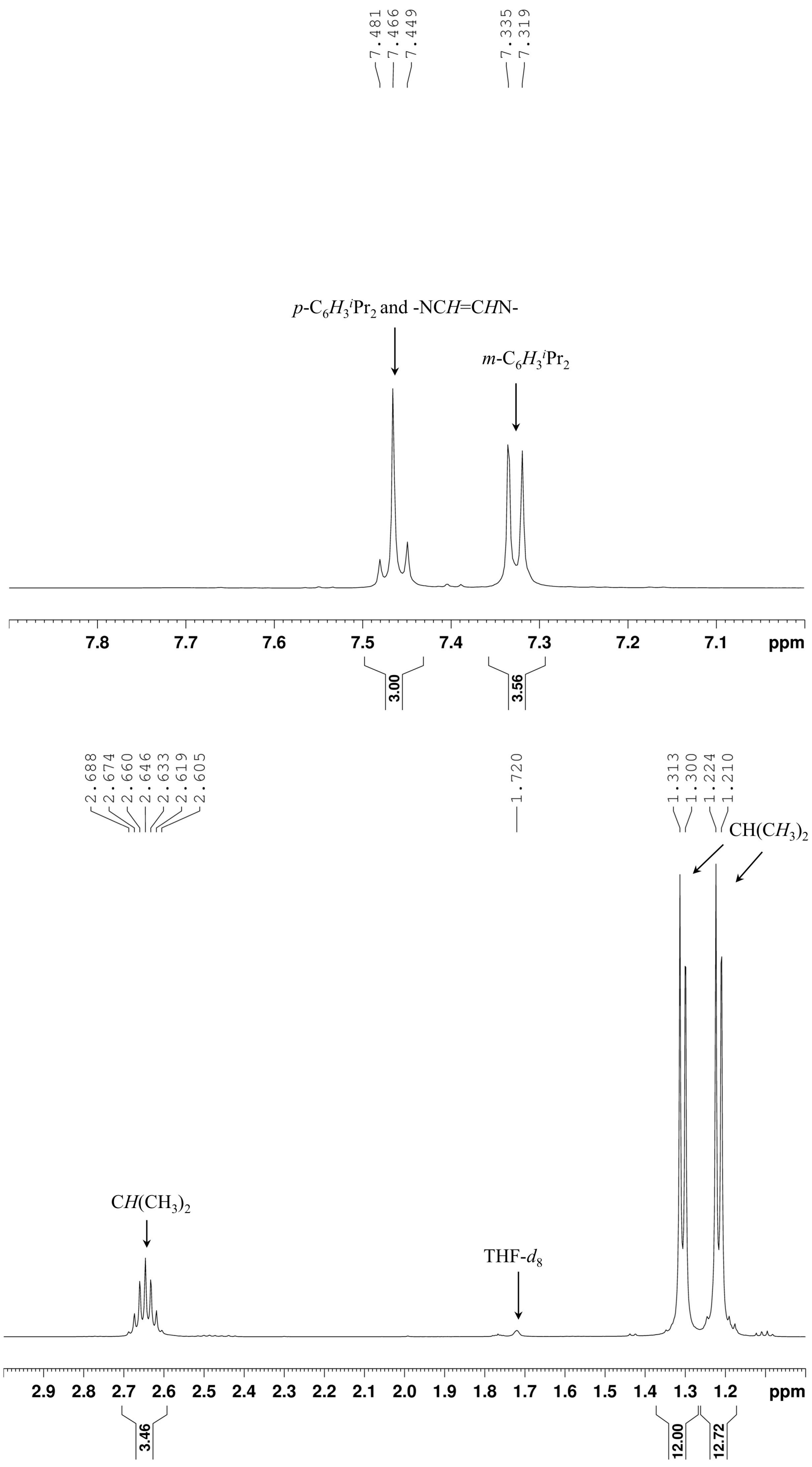

Figure S28. Expanded ^{1}H-NMR spectrum of compound **7** in THF-d_8.

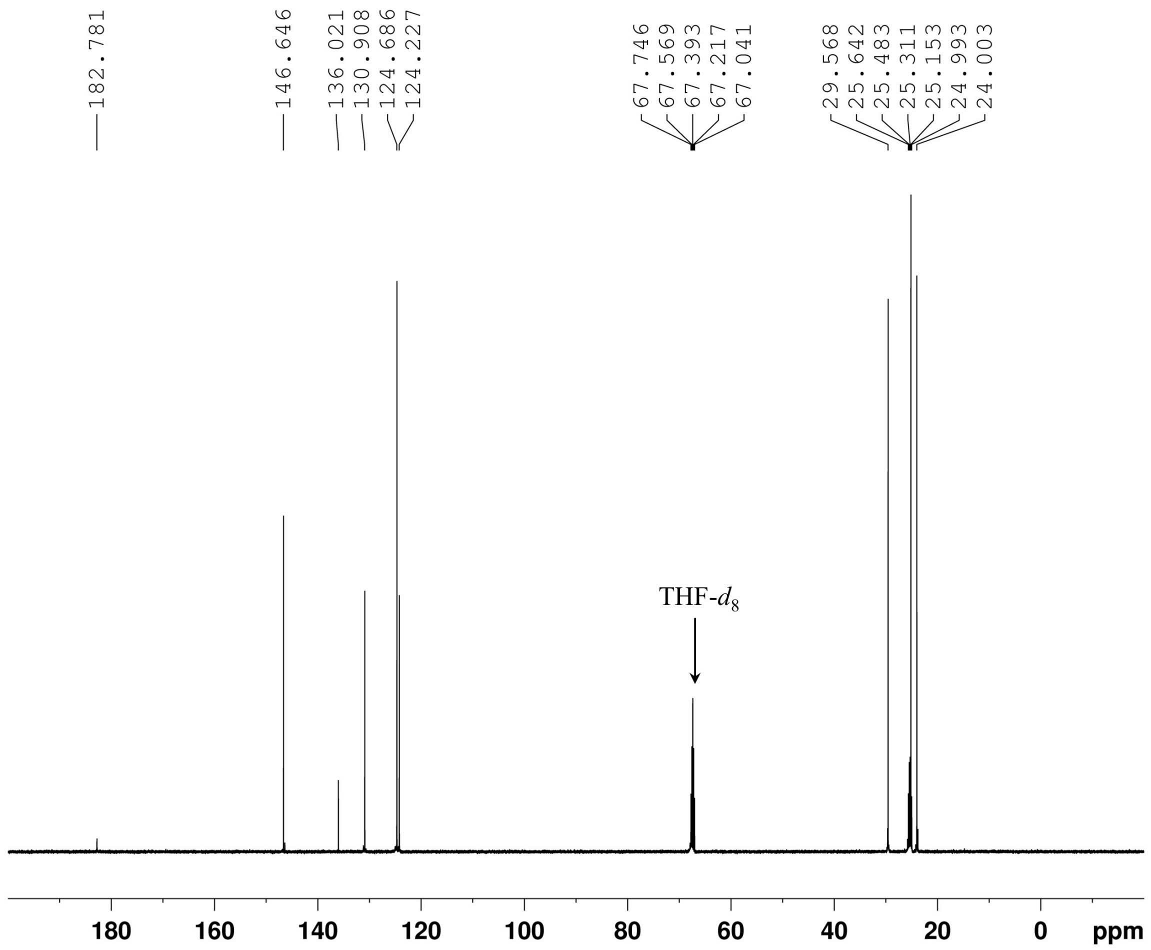

Figure S29. $^{13}C\{^1H\}$-NMR spectrum of compound **7** in THF-d_8.

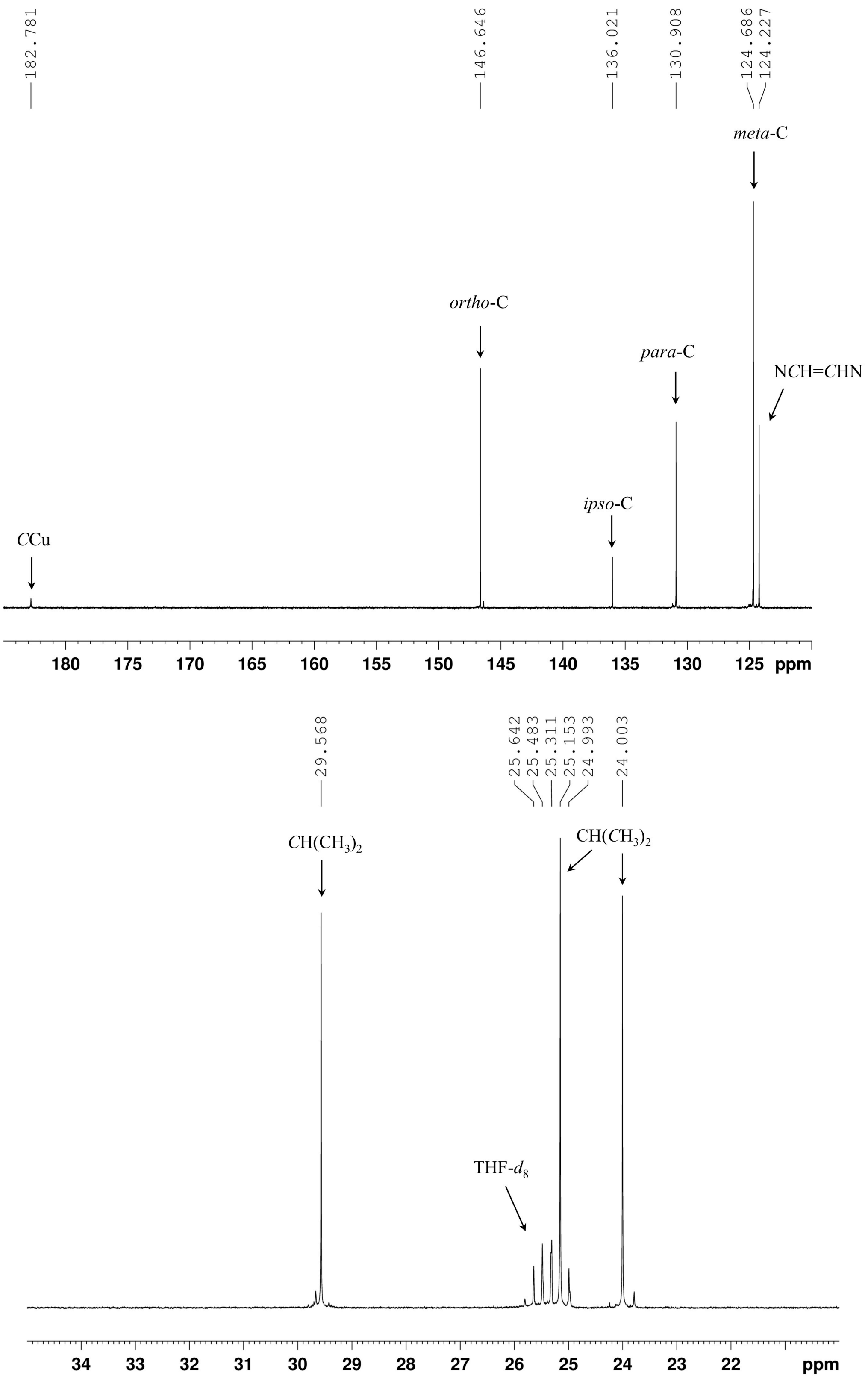

Figure S30. Expanded $^{13}C\{^{1}H\}$-NMR spectrum of compound **7** in THF-d_8.

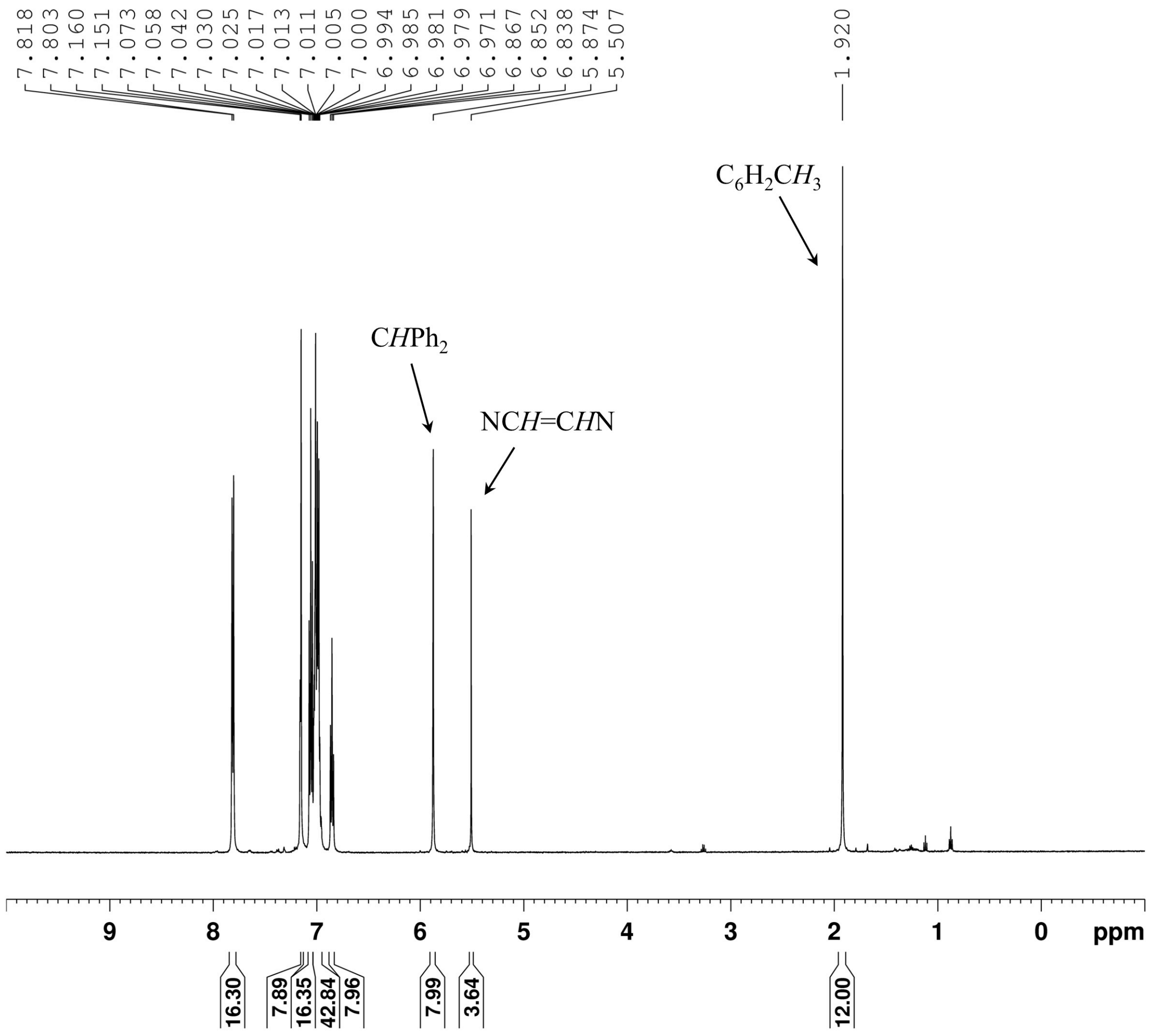

Figure S31. ^{1}H-NMR spectrum of compound **1** in C_6D_6.

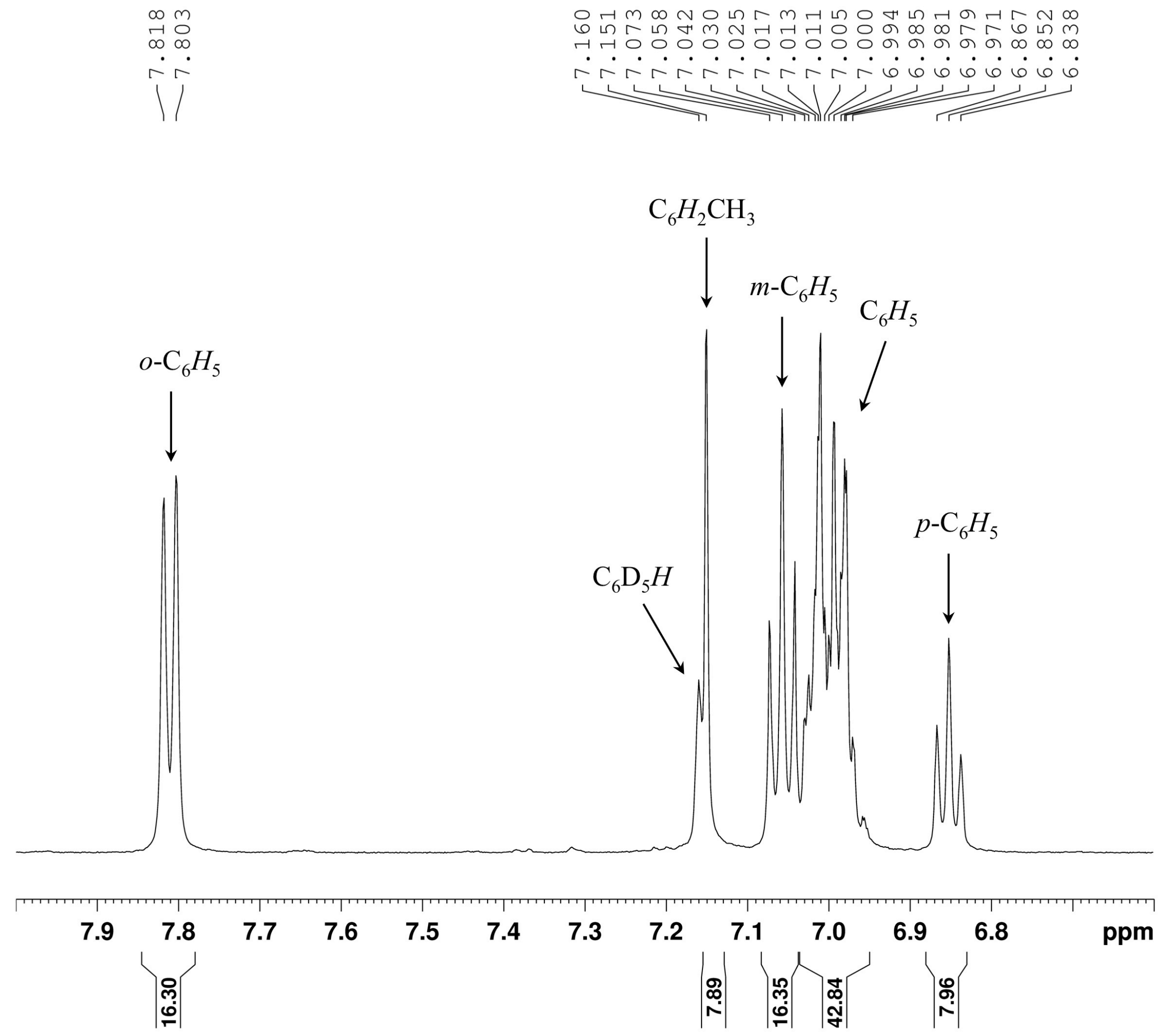

Figure S32. Expanded ^{1}H-NMR spectrum of compound **1** in C_6D_6.

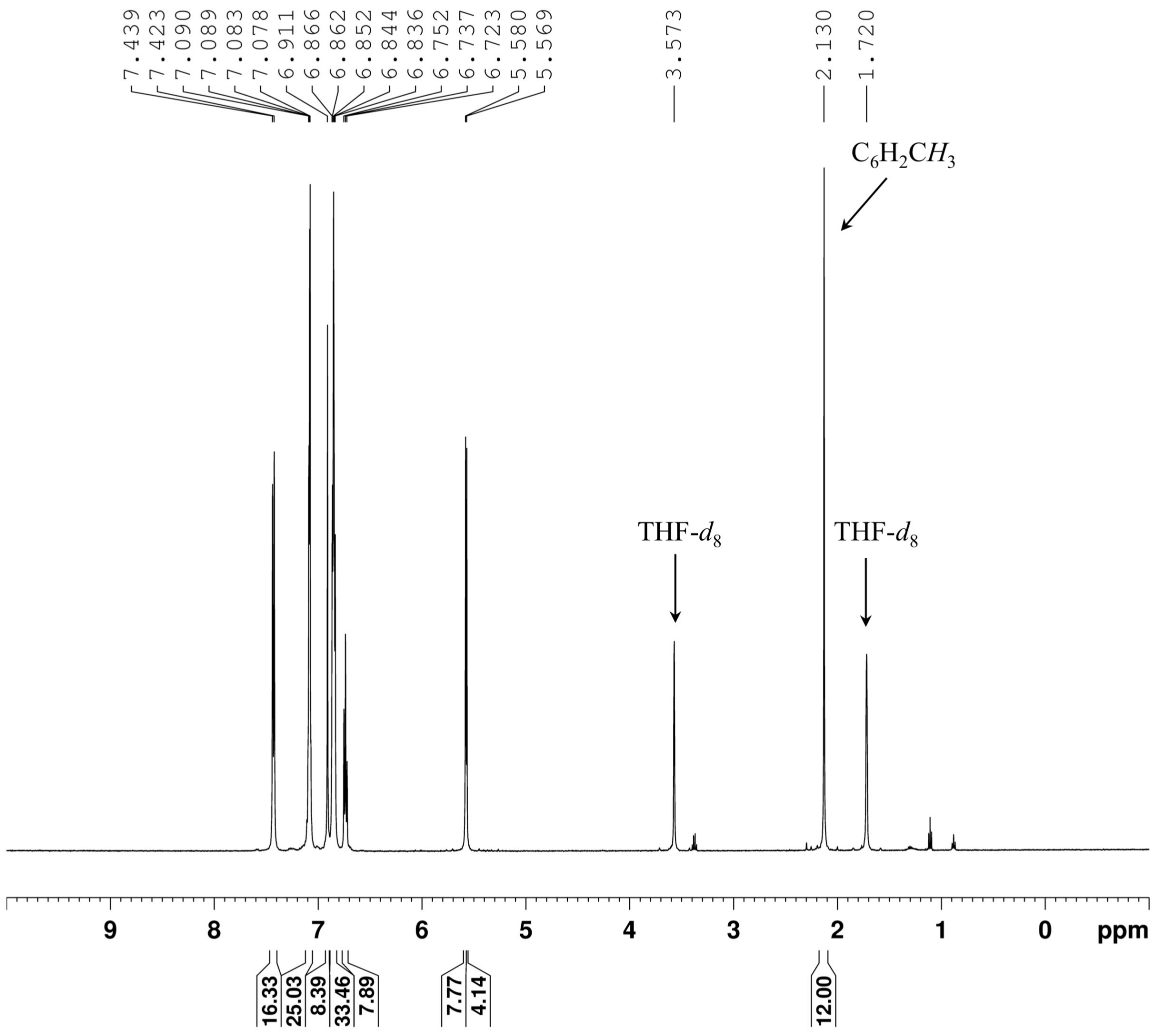

Figure S33. ^{1}H-NMR spectrum of compound **1** in THF-d_8.

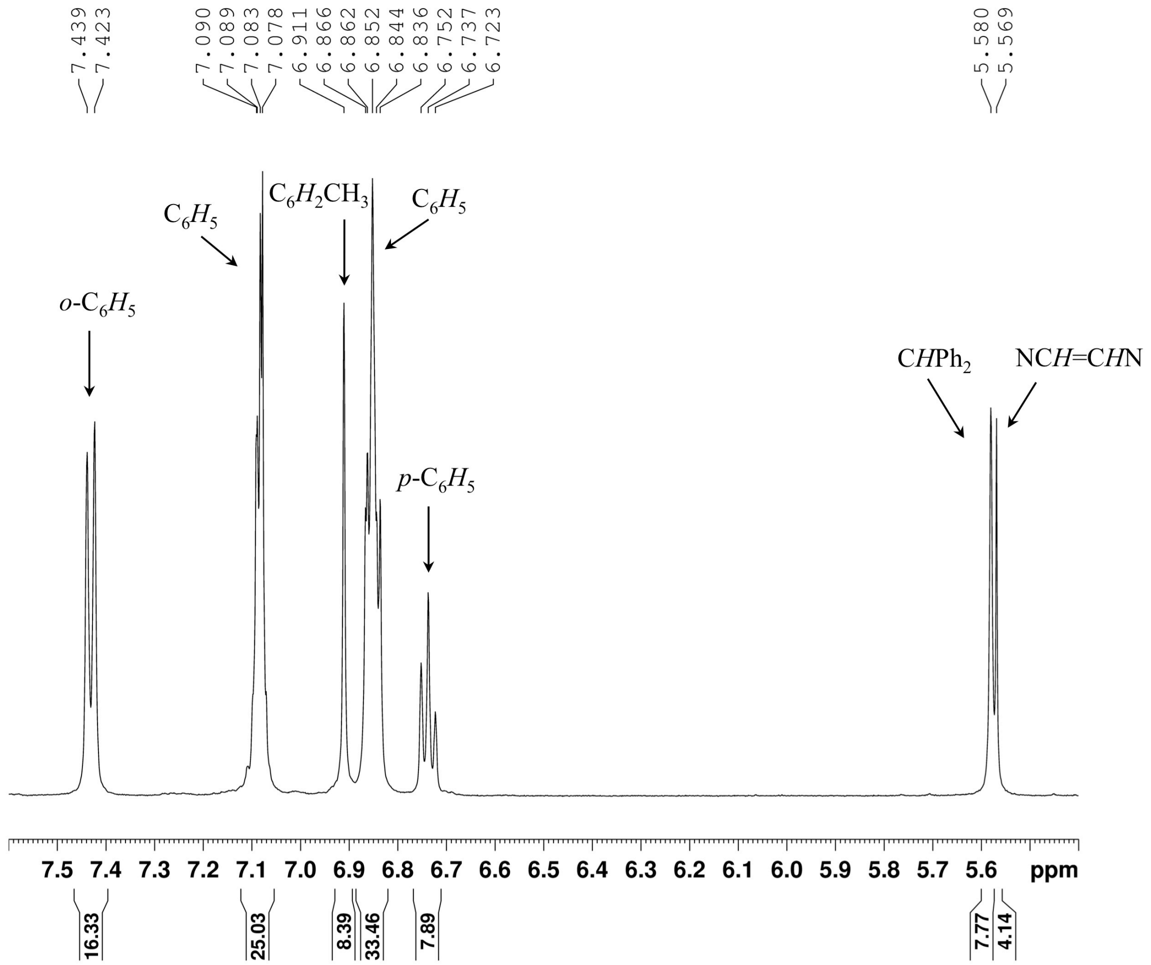

Figure S34. Expanded ^{1}H-NMR spectrum of compound **1** in THF-d_8.

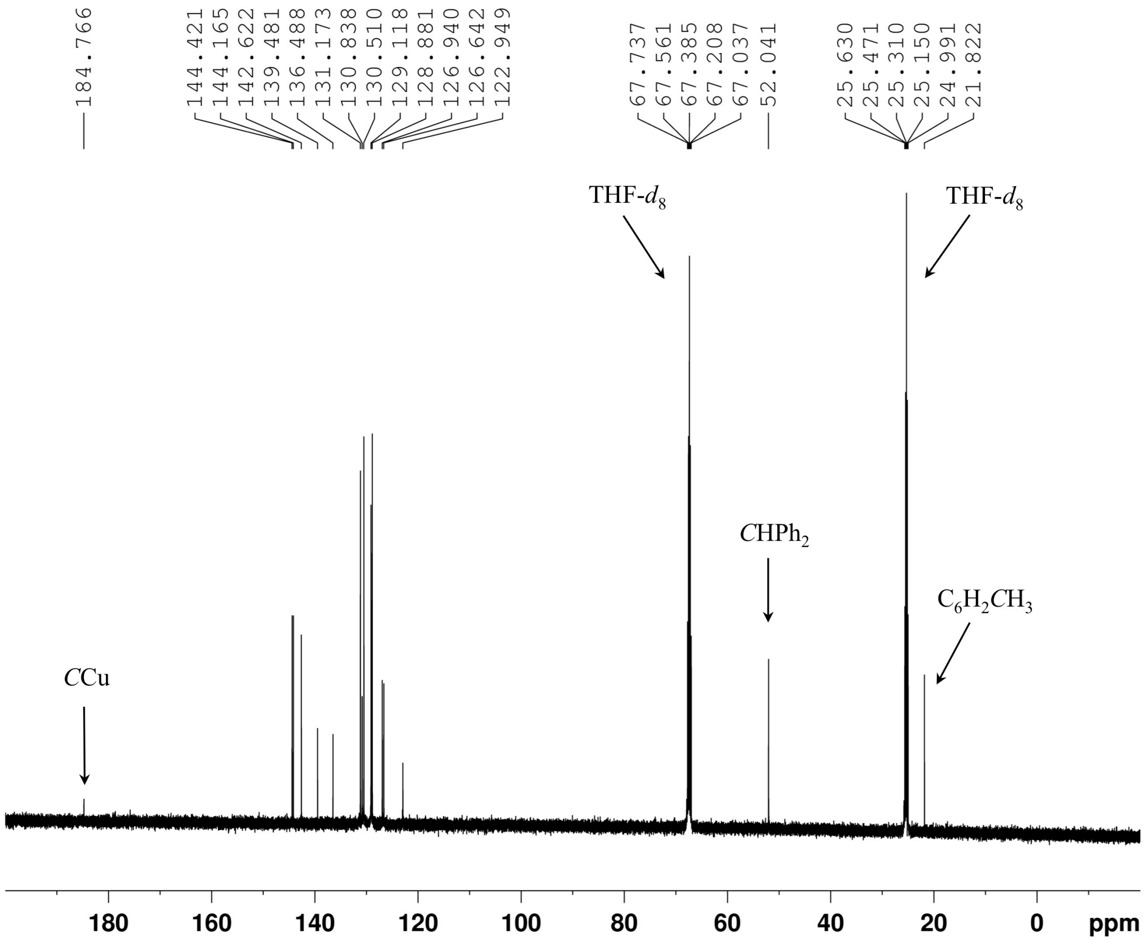

Figure S35. $^{13}C\{^1H\}$-NMR spectrum of compound **1** in THF-d_8.

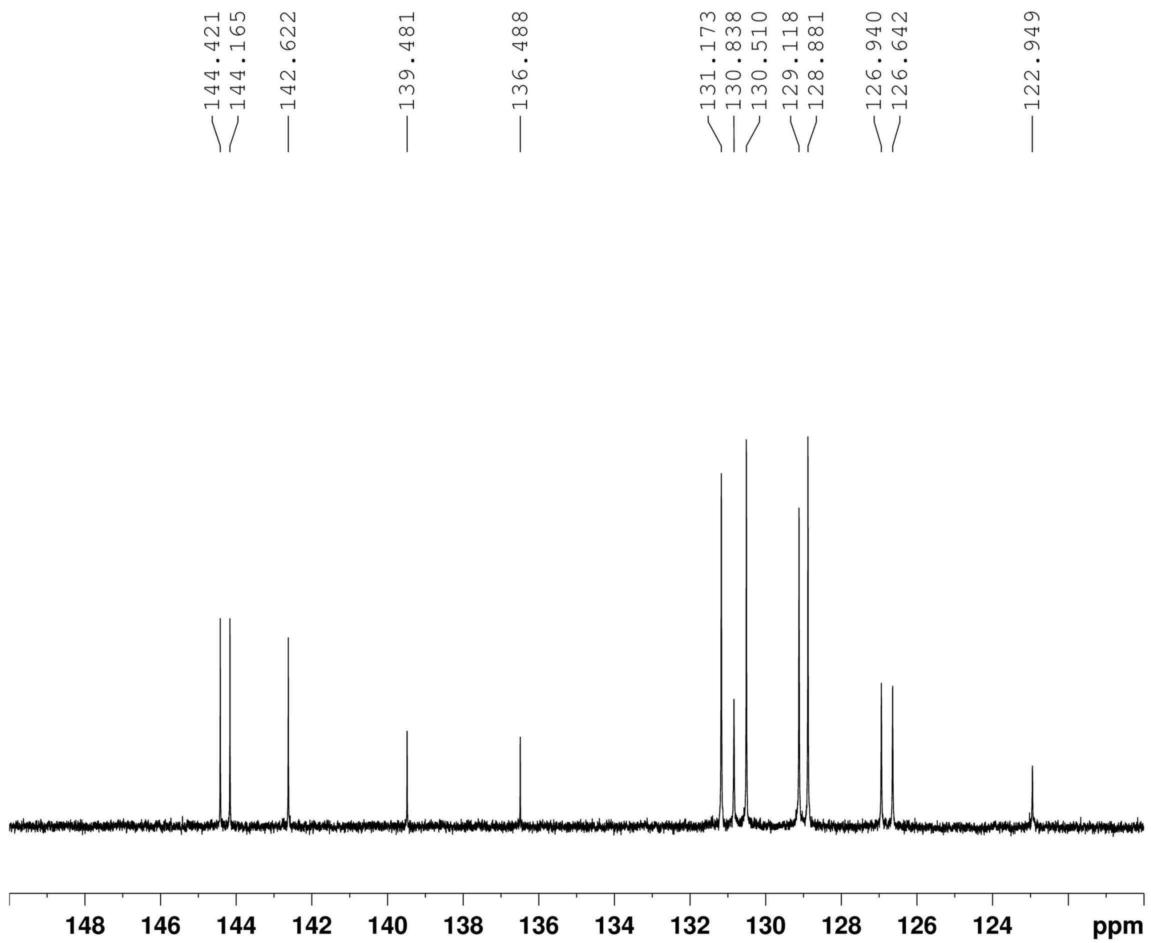

Figure S36. Expanded $^{13}C\{^{1}H\}$-NMR spectrum of compound **1** in THF-d_8.

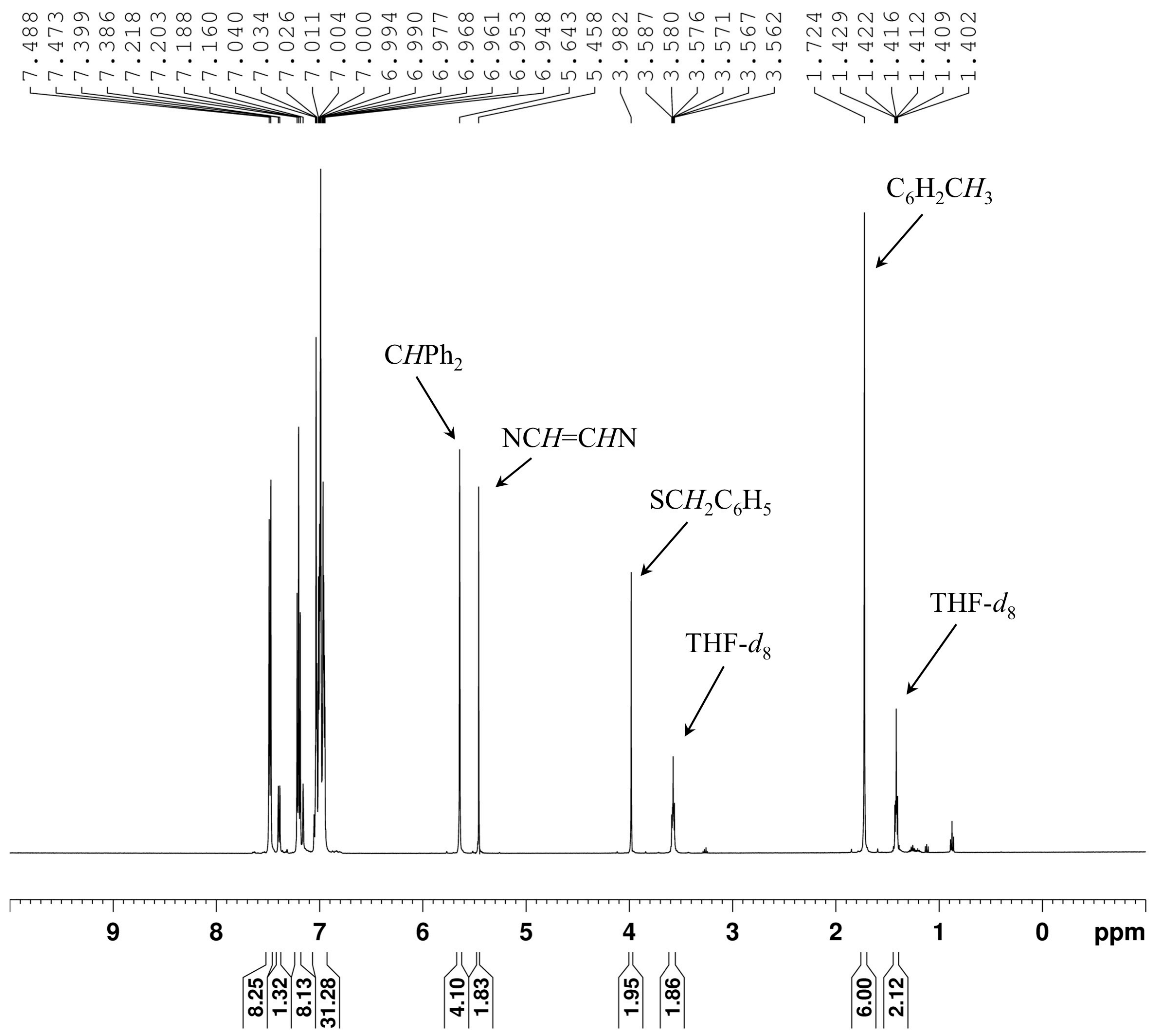

Figure S37. ^{1}H-NMR spectrum of compound **8** in C_6D_6.

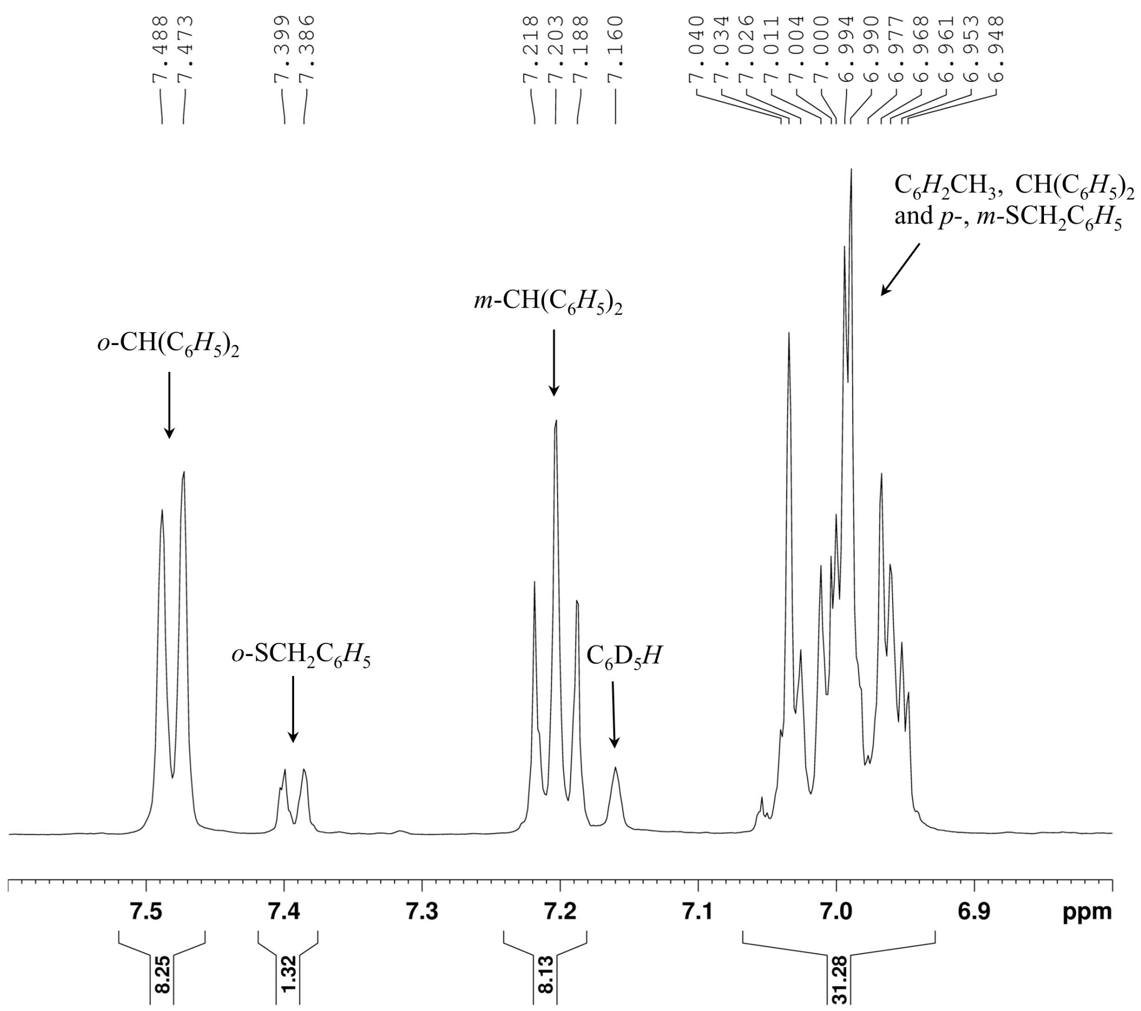

Figure S38. Expanded ^{1}H-NMR spectrum of compound **8** in C_6D_6.

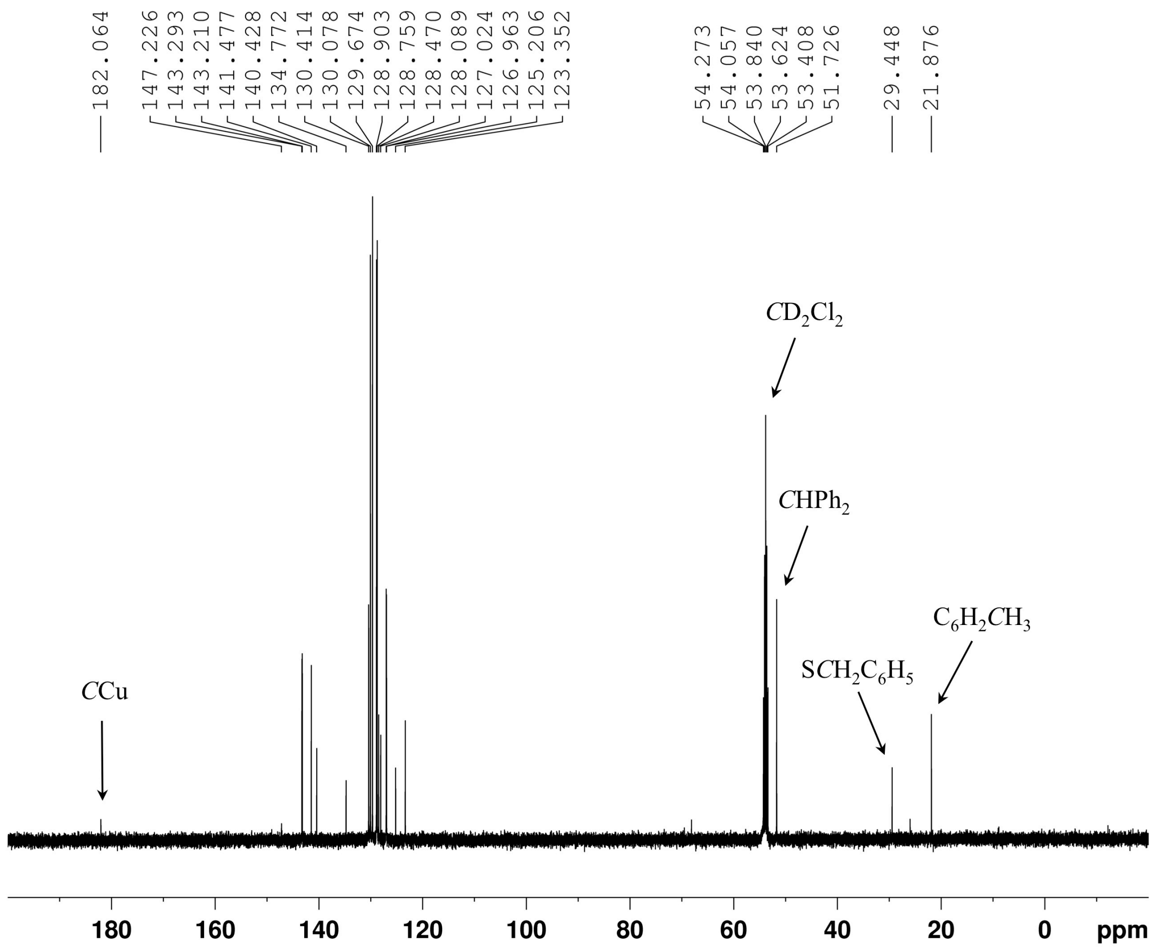

Figure S39. $^{13}C\{^1H\}$-NMR spectrum of compound **8** in CD_2Cl_2.

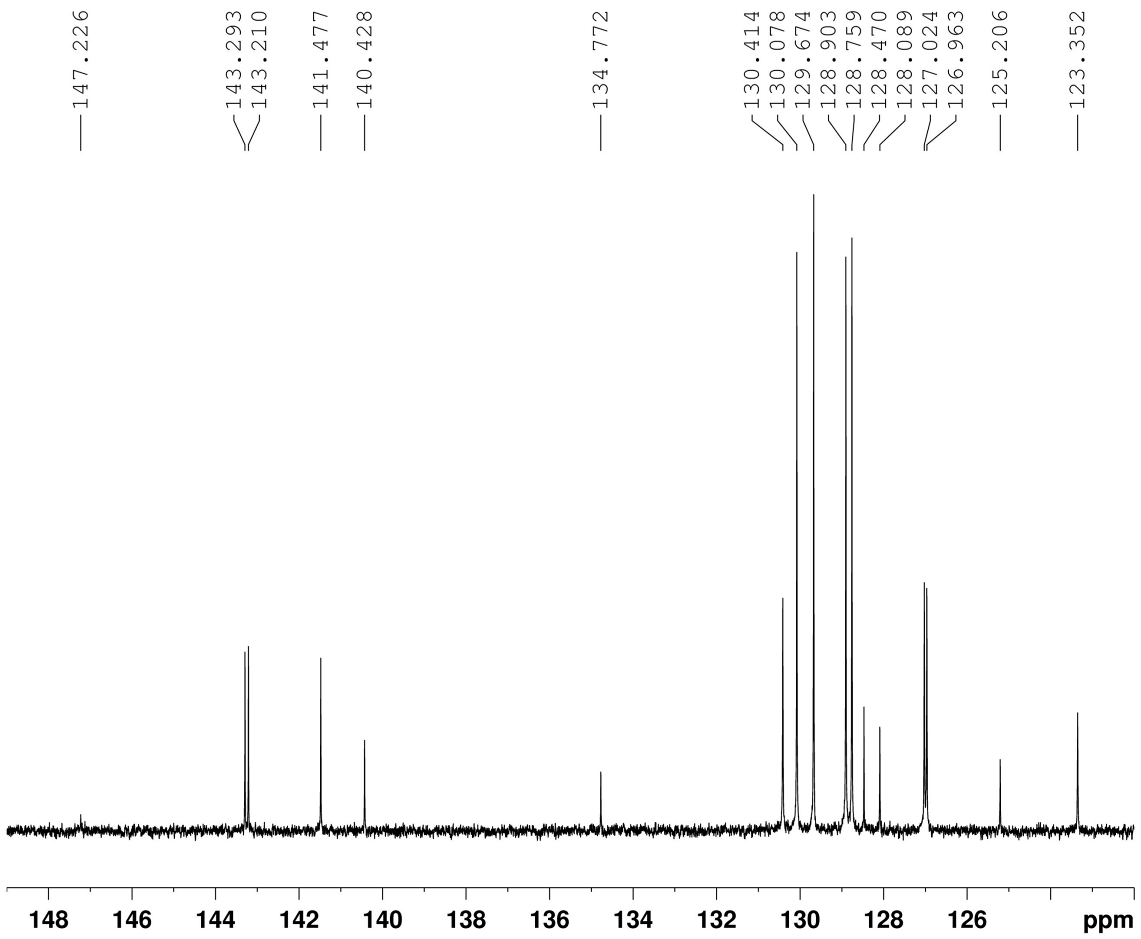

Figure S40. Expanded $^{13}C\{^1H\}$-NMR spectrum of compound **8** in CD_2Cl_2.

3. X-ray Data Collection and Structure Refinement

X-ray diffraction data for **1** and **7** were collected at 100 K on a Bruker D8 VENTURE with PHOTON 100 CMOS detector system equipped with a Mo-target X-ray tube (λ = 0.71073 Å). X-ray diffraction data for **6** were collected at 100 K on a Bruker SMART APEX system with a Charged Coupled Device (CCD) detector and a Mo-target X-ray tube (λ = 0.71073 Å). The data were collected using a routine of ϕ and ω scans to survey an entire sphere of reciprocal space, indexed using the APEX2 (Bruker AXS, version 2014.9-0, 2014) program suites and corrected for absorption effects using the empirical methods as implemented in SADABS (Bruker AXS, version 2014/4, 2014). The structures of **1**, **6** and **7** were solved by SHELXT[11] and refined by full-matrix least-squares procedures using the Bruker SHELXTL (version 6.14) software package (XL refinement program version 2014/7) [12]. All non-hydrogen atoms were refined with anisotropic thermal parameters. All hydrogen atoms were included at calculated positions as a riding model except the one attached to the sulfur atom in **6** and **7**. Finding the exact location of hydrogen atom in the hydrosulfido ligand was problematic as several residual peaks were observed around the sulfur atom. One of the peaks was selected and introduced as a hydrogen atom. It was refined with the S–H bond length restrained at 1.27 Å and the Cu···H distance restrained at 2.68 Å using the default standard deviation values.[13] All structures are drawn with thermal ellipsoids at 50% probability. Crystallographic data and details of the data collection and structure refinement are listed in Table S1–S3.

Compound **1** is crystallized in a non-centrosymmetric *Cc* space group and refined as a 2-component inversion twin with two essentially equal domains. The {Cu_2S} fragment is slightly positionally disordered with one of the orientations being strongly preferred (ca. 91%). The anisotropic displacement parameters for Cu atoms of the minor orientation were constrained to be identical to those of the major orientation. Additional ADPs restraints, such as ISOR and

RIGU,[14] were also utilized for some other parts of the molecule. Furthermore, disorder was observed in the position of one of the phenyl rings. The disordered phenyl ring was modeled with two orientations (0.55:0.45) using geometric restraints such as SAME, SADI and FLAT to make the phenyl ring flat and C–C distances equal to each other within the 0.01 Å standard deviation.

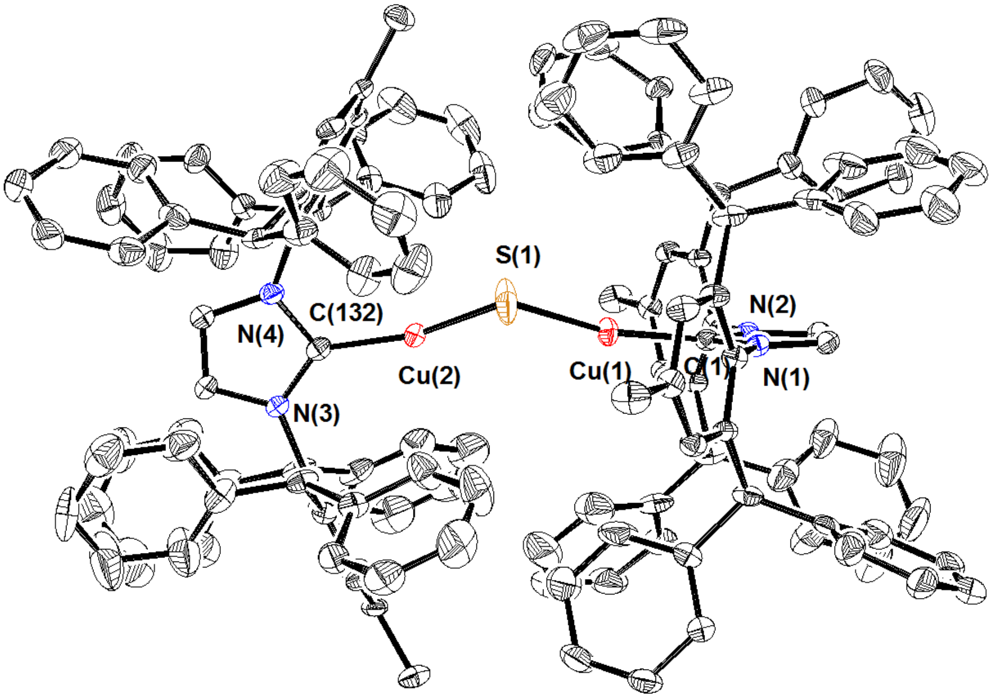

Figure S41. X-ray crystal structure of **1** (50% probability ellipsoids). Hydrogen atoms are omitted for clarity. Selected bond lengths (Å) and bond angles (°): Cu(1)−C(1), 1.873(5); Cu(2)−C(132), 1.869(5); Cu(1)−S(1), 2.0787(17); Cu(2)−S(1), 2.0848(18); Cu(1)···Cu(2), 3.6085(9); C(1)−Cu(1)−S(1), 162.92(16); C(132)−Cu(2)−S(1), 158.81(16); Cu(1)−S(1)−Cu(2), 120.15(9). The dihedral angle between the IPr* imidazole rings is 89.9(3)°.

Table S1. Crystal and Refinement Data for **1**.

Empirical formula	$C_{138}H_{112}Cu_2N_4S$
Formula weight	1985.45
Temperature (K)	100(2)
Wavelength (Å)	0.71073
Crystal system	Monoclinic
Space group	*Cc*
Unit cell dimensions	a = 19.830(4) Å
	b = 18.358(4) Å
	c = 28.487(6) Å
	α = 90°
	β = 95.68(3)°
	γ = 90°
Volume ($Å^3$)	10320(4)
Z	4
Density (calculated) (Mg/m^3)	1.278
Absorption coefficient (mm^{-1})	0.489
F(000)	4168
Crystal size (mm^3)	0.46 x 0.42 x 0.28
Theta range for data collection	1.515 to 25.260°
Index ranges	-23<=h<=23, -21<=k<=21, -33<=l<=33
Reflections collected	47829
Independent reflections	17887 [R(int) = 0.1020]
Completeness to theta = 25.000°	99.60%
Absorption correction	Semi-empirical from equivalents
Max. and min. transmission	0.7452 and 0.6549
Refinement method	Full-matrix least-squares on F^2
Data / restraints / parameters	17887 / 6 / 1100
Goodness-of-fit on F^2	1.077
Final R indices [I>2sigma(I)]	R1 = 0.0897, wR2 = 0.1982
R indices (all data)	R1 = 0.1514, wR2 = 0.2267
Absolute structure parameter	0.5
Extinction coefficient	noref
Largest diff. peak and hole (e.$Å^{-3}$)	1.132 and -0.633

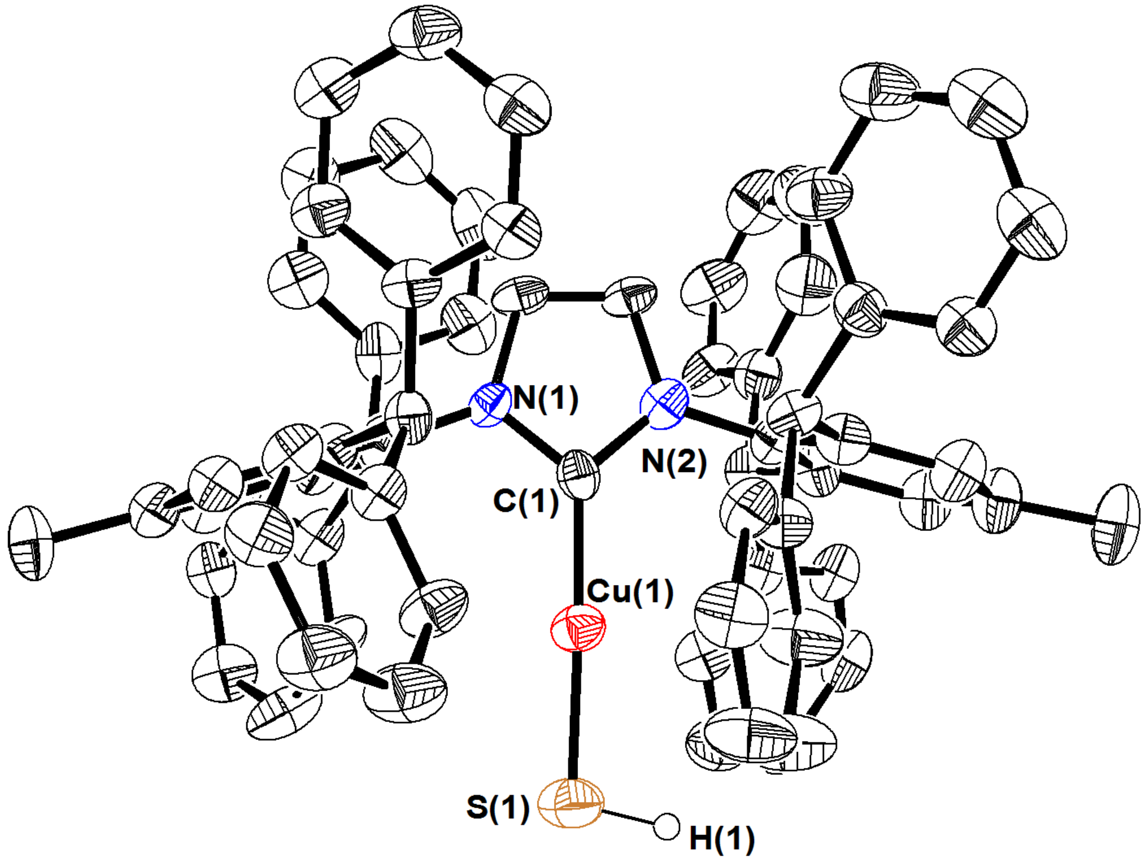

Figure S42 X-ray crystal structure of **6** (50% probability ellipsoids). Hydrogen atoms, except on S(1), and interstitial CH_2Cl_2 molecules are omitted for clarity. Selected bond lengths (Å) and bond angles (°): Cu(1)−C(1), 1.843(5); Cu(1)−S(1), 2.0800(18); C(1)−Cu(1)−S(1), 175.62(18).

Table S2. Crystal and Refinement Data for **6**·CH_2Cl_2.

Empirical formula	$C_{70}H_{59}Cl_2CuN_2S$
Formula weight	1094.69
Temperature (K)	100(2)
Wavelength (Å)	0.71073
Crystal system	Monoclinic
Space group	$P2_1/c$
Unit cell dimensions	a = 12.650(3) Å
	b = 18.177(5) Å
	c = 23.842(6) Å
	α = 90°
	β = 91.587(3)°
	γ = 90°
Volume ($Å^3$)	5480(2)
Z	4
Density (calculated) (Mg/m^3)	1.327
Absorption coefficient (mm^{-1})	0.580
F(000)	2288
Crystal size (mm^3)	0.44 x 0.40 x 0.18
Theta range for data collection	1.409 to 25.461°
Index ranges	$-15 \leq h \leq 15$, $-21 \leq k \leq 21$, $-28 \leq l \leq 28$
Reflections collected	51706
Independent reflections	9942 [R(int) = 0.1381]
Completeness to theta = 25.242°	99.7%
Absorption correction	Semi-empirical from equivalents
Max. and min. transmission	0.7452 and 0.5176
Refinement method	Full-matrix least-squares on F^2
Data / restraints / parameters	9942 / 2 / 690
Goodness-of-fit on F^2	1.056
Final R indices [I>2sigma(I)]	R1 = 0.0833, wR2 = 0.1864
R indices (all data)	R1 = 0.1443, wR2 = 0.2134
Extinction coefficient	noref
Largest diff. peak and hole (e.$Å^{-3}$)	1.350 and –0.622

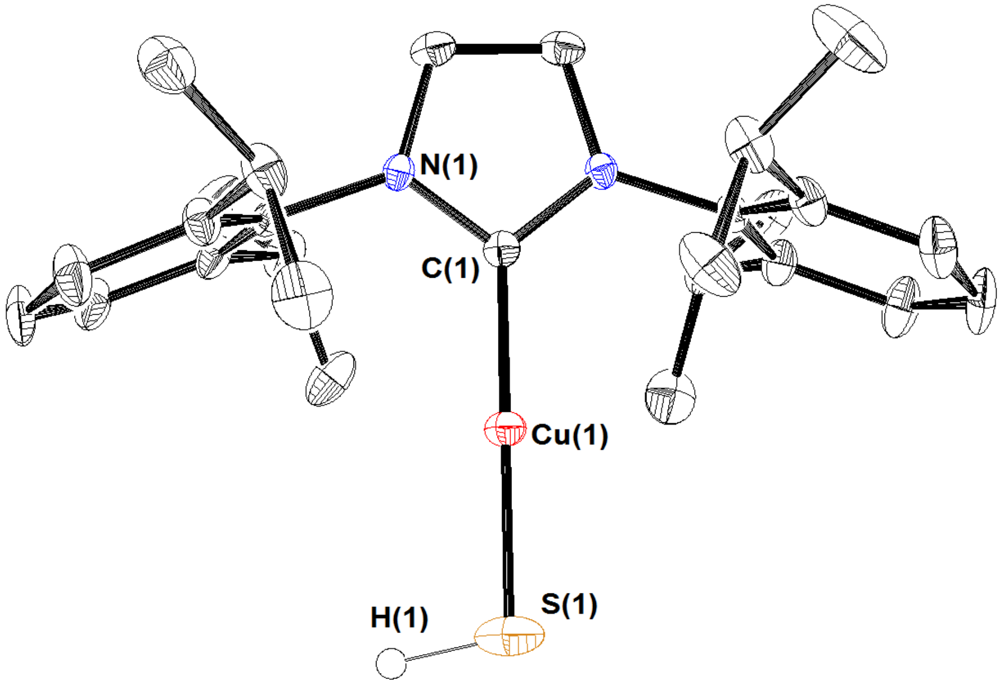

Figure S43. X-ray crystal structure of **7** (50% probability ellipsoids). Hydrogen atoms are omitted for clarity. Selected bond lengths (Å) and bond angles (°): Cu(1)−C(1), 1.890(4); Cu(1)−S(1), 2.1270(12); C(1)−Cu(1)−S(1), 180.0.

Table S3. Crystal and Refinement Data for **7**.

Empirical formula	$C_{27}H_{37}CuN_2S$
Formula weight	485.18
Temperature (K)	100(2)
Wavelength (Å)	0.71073
Crystal system	Orthorhombic
Space group	*Pccn*
Unit cell dimensions	a = 12.6538(10) Å
	b = 10.6947(9) Å
	c = 19.3375(15) Å
	α = 90°
	β = 90°
	γ = 90°
Volume (Å^3)	2616.9(4)
Z	4
Density (calculated) (Mg/m^3)	1.231
Absorption coefficient (mm^{-1})	0.930
F(000)	1032
Crystal size (mm^3)	0.418 x 0.275 x 0.258
Theta range for data collection	2.494 to 27.909°
Index ranges	–16≤ h ≤ 16, –14 ≤ k ≤ 14, –24 ≤ l ≤ 24
Reflections collected	22356
Independent reflections	3102 [R(int) = 0.0284]
Completeness to theta = 25.242°	99.7%
Absorption correction	Semi-empirical from equivalents
Max. and min. transmission	0.746 and 0.689
Refinement method	Full-matrix least-squares on F^2
Data / restraints / parameters	3102 / 2 / 149
Goodness-of-fit on F^2	1.040
Final R indices [I>2sigma(I)]	R1 = 0.0561, wR2 = 0.1260
R indices (all data)	R1 = 0.0606, wR2 = 0.1284
Extinction coefficient	noref
Largest diff. peak and hole (e.Å^{-3})	1.428 and –0.505

4. DFT calculations

Density functional theory calculations employed the Gaussian 09 suite of programs.[15] The experimental X–ray crystallographic data for **1** were used as the input geometry. The optimized structure of **1** (vide infra) was modified by Avogadro[16] to generate the input geometry of **2**. Both geometries were optimized at the B3LYP[17] level of theory without symmetry constraints. All atoms were described with the 6–31G* basis set,[18] which has been shown to provide accurate molecular geometries for other (NHC)Cu^{I} compounds.[19] No imaginary frequencies were obtained in subsequent vibrational calculations, confirming that the optimized structures reside at potential-surface minima.

The optimized geometry of **1** (Figure S44) is in reasonable agreement with the X-ray crystal structure (Table S4). The calculated HOMO and HOMO−1 are sulfur p orbital in character, with small π* contributions from Cu d orbitals (Figure S45).

The optimized structure of **2** (Figure S46) exhibits Cu–C and Cu–S bond distances of 1.84 Å and 2.12 Å, respectively, and C–Cu–S bond angles of ~166.5°. These geometric parameters are similar to those in the X-ray structure and DFT optimized structure of **1**. The Cu−S−Cu angle in the optimized structure of **2** is about 9° smaller than that in the X-ray structure of **1**, reflecting the decreased steric demands of the IPr ligand.

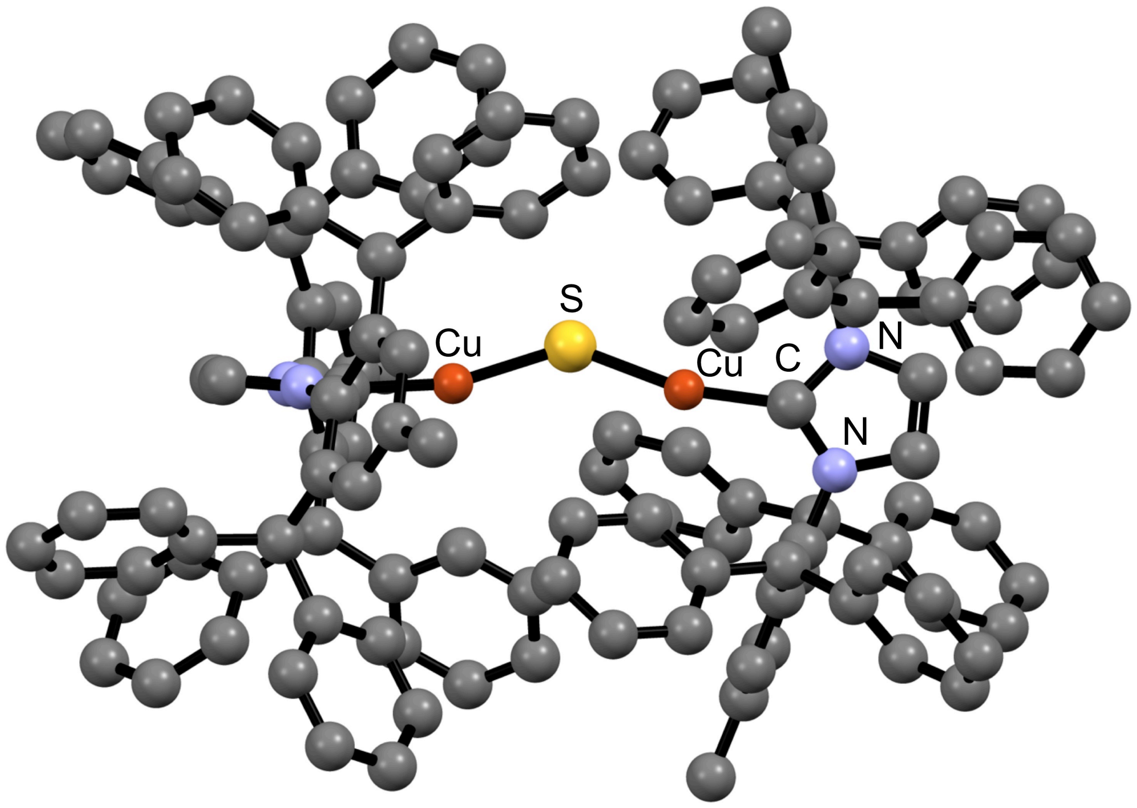

Figure S44. DFT (B3LYP/6–31G*) optimized structure of **1**. Hydrogen atoms are omitted for clarity. For selected geometric parameters, see Table S4.

Table S4. Comparison of Selected Bond Distances and Bond Angles in the X-ray Structure of **1** and Optimized Structure of **1** and **2**.

	Cu–C, Å	Cu–S, Å	C–Cu–S, °	Cu–S–Cu, °
**X-ray Structure of 1**	1.873(5), 1.869(5)	2.0787(17), 2.0848(18)	162.92(16), 158.81(16)	120.15(9)
**Optimized Structure of 1**	1.851, 1.855	2.126, 2.129	164.43, 165.12	137.03
**Optimized Structure of 2**	1.837, 1.838	2.117, 2.118	166.79, 166.33	111.36

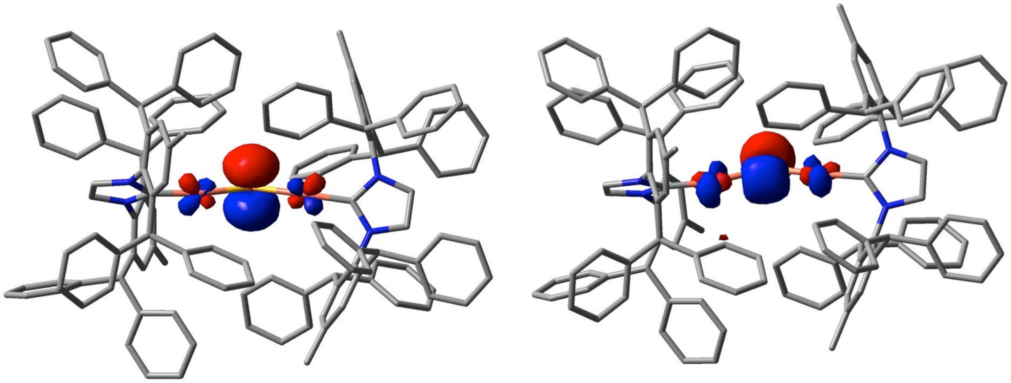

HOMO HOMO-1

Figure S45. DFT calculated HOMO (left) and HOMO−1 (right) of **1** (B3LYP/6-31G*). Isovalue = 0.05.

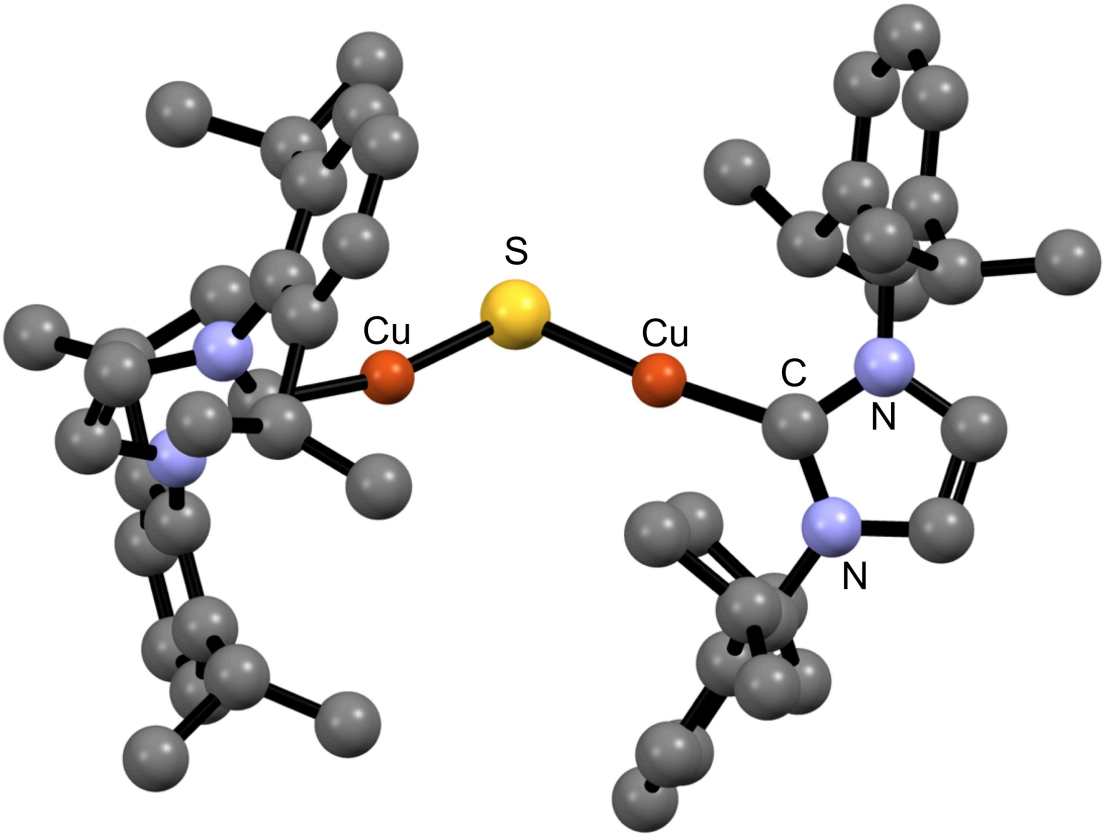

Figure S46. DFT (B3LYP/6–31G*) optimized structure of **2**. Hydrogen atoms are omitted for clarity. For selected geometric parameters, see Table S4.

Table S5. Calculated (DFT) Cartesian Coordinates of **1**.

Cu	-1.9864	0.2093	0.0406	C	-9.2599	-1.6961	-3.4502
Cu	1.9713	0.3023	-0.0238	H	-10.3314	-1.8517	-3.5442
S	-0.0308	0.9797	-0.2789	C	-8.654	-0.5913	-4.0471
N	-4.7721	0.49	-0.6605	H	-9.2518	0.1204	-4.611
N	-4.5586	-0.5384	1.2161	C	-7.2764	-0.387	-3.9218
N	4.5168	-0.8231	-0.9115	H	-6.8223	0.4844	-4.3837
N	4.7806	0.8309	0.4369	C	-4.2345	3.3436	-0.6913
C	-3.8197	0.0084	0.202	H	-3.7817	2.7427	0.105
C	-6.0599	0.25	-0.1899	C	-3.3991	4.6273	-0.7626
H	-6.9347	0.5672	-0.7332	C	-2.0243	4.5563	-0.4929
C	-5.9266	-0.4022	0.989	H	-1.5649	3.5984	-0.2552
H	-6.6608	-0.7706	1.6873	C	-1.2334	5.7047	-0.5358
C	-4.4699	1.1161	-1.9251	H	-0.1714	5.6275	-0.3248
C	-4.513	0.3356	-3.0951	C	-1.8007	6.9403	-0.8556
C	-4.1824	0.95	-4.3074	H	-1.1831	7.8345	-0.8864
H	-4.1835	0.3503	-5.2134	C	-3.1657	7.0191	-1.1315
C	-3.836	2.302	-4.379	H	-3.62	7.9758	-1.378
C	-3.8469	3.0561	-3.2014	C	-3.9596	5.8707	-1.0821
H	-3.5908	4.1099	-3.2427	H	-5.0247	5.948	-1.279
C	-4.1643	2.4911	-1.9638	C	-5.6833	3.6138	-0.2687
C	-3.4278	2.925	-5.6932	C	-6.7283	3.7388	-1.1936
H	-3.584	4.0085	-5.6895	H	-6.5248	3.62	-2.2541
H	-3.99	2.5002	-6.532	C	-8.0326	4.0062	-0.7681
H	-2.3625	2.7474	-5.8916	H	-8.8285	4.0993	-1.503
C	-4.9604	-1.129	-3.0522	C	-8.3141	4.1482	0.5906
H	-4.7165	-1.4982	-2.0515	H	-9.3297	4.3475	0.9223
C	-4.1745	-2.0201	-4.0216	C	-7.28	4.0282	1.5219
C	-4.6669	-2.3928	-5.2783	H	-7.4857	4.122	2.5845
H	-5.6558	-2.0671	-5.5874	C	-5.9793	3.7688	1.0925
C	-3.9063	-3.1968	-6.1313	H	-5.1769	3.6846	1.8216
H	-4.3065	-3.4768	-7.1026	C	-4.0031	-1.1943	2.3708
C	-2.6456	-3.6458	-5.7377	C	-3.8512	-0.469	3.5652
H	-2.0566	-4.2739	-6.4008	C	-3.3434	-1.1416	4.6826
C	-2.1483	-3.2852	-4.4836	H	-3.2169	-0.5866	5.6086
H	-1.169	-3.627	-4.1606	C	-2.9862	-2.4911	4.6353
C	-2.9071	-2.4802	-3.6353	C	-3.1307	-3.1729	3.4226
H	-2.5069	-2.2033	-2.6628	H	-2.825	-4.2139	3.3567
C	-6.4808	-1.2862	-3.2006	C	-3.6389	-2.5518	2.2795
C	-7.104	-2.3925	-2.6033	C	-2.4174	-3.1918	5.8472
H	-6.5073	-3.0988	-2.0318	H	-1.3205	-3.2053	5.8126
C	-8.4769	-2.5978	-2.7251	H	-2.7537	-4.2329	5.9017
H	-8.9346	-3.4597	-2.2464	H	-2.7109	-2.6903	6.7752

C	-3.7551	-3.3263	0.9663
H	-3.9608	-2.5855	0.1886
C	-2.3957	-3.9329	0.5945
C	-1.3978	-3.07	0.1181
H	-1.5983	-2.0002	0.0267
C	-0.1361	-3.5597	-0.2081
H	0.6229	-2.8723	-0.5715
C	0.1513	-4.9193	-0.0562
H	1.1412	-5.2956	-0.2943
C	-0.8342	-5.7822	0.4216
H	-0.6189	-6.8406	0.5467
C	-2.1024	-5.2923	0.7447
H	-2.8678	-5.9747	1.1029
C	-4.9228	-4.3178	0.906
C	-5.7637	-4.5958	1.9886
H	-5.5883	-4.1146	2.9453
C	-6.8339	-5.4873	1.8569
H	-7.4723	-5.6863	2.7141
C	-7.0826	-6.1164	0.6393
H	-7.913	-6.81	0.5382
C	-6.2514	-5.8464	-0.4521
H	-6.4309	-6.33	-1.409
C	-5.187	-4.9574	-0.3183
H	-4.5402	-4.7617	-1.1705
C	-4.1891	1.0204	3.6515
H	-4.4851	1.3316	2.6444
C	-2.9146	1.8198	3.9668
C	-2.6409	2.355	5.2315
H	-3.36	2.2396	6.0371
C	-1.4562	3.0587	5.4634
H	-1.2634	3.4715	6.4508
C	-0.5272	3.2293	4.4376
H	0.3959	3.7728	4.6214
C	-0.7815	2.6787	3.18
H	-0.0618	2.7626	2.3703
C	-1.9639	1.9801	2.9477
H	-2.1016	1.5198	1.9684
C	-5.4008	1.3374	4.5402
C	-6.242	0.3442	5.0572
H	-6.0075	-0.7016	4.8887
C	-7.3839	0.6742	5.7936
H	-8.016	-0.1197	6.1836
C	-7.71	2.0077	6.0293
H	-8.5947	2.2651	6.6055
C	-6.8834	3.0111	5.5172
H	-7.12	4.0572	5.6951
C	-5.7478	2.6786	4.7803
H	-5.1113	3.4707	4.3968
C	4.49	1.9315	1.3177
C	4.2188	1.6765	2.6725
C	3.8387	2.7564	3.4769
H	3.5971	2.5687	4.5196
C	3.7166	4.0511	2.9663
C	4.017	4.267	1.6171
H	3.8942	5.2622	1.1977
C	4.4362	3.2307	0.7791
C	3.2388	5.1891	3.8384
H	3.8074	6.1068	3.6505
H	3.3311	4.9445	4.9014
H	2.1835	5.4182	3.6415
C	4.8142	3.4893	-0.6789
H	4.9218	2.5095	-1.1489
C	6.1849	4.1586	-0.8502
C	6.7	5.1316	0.0164
H	6.1345	5.4361	0.8901
C	7.9511	5.7098	-0.2136
H	8.3274	6.4616	0.4757
C	8.7168	5.3234	-1.3132
H	9.6913	5.7714	-1.4888
C	8.2201	4.349	-2.1807
H	8.8072	4.0289	-3.0379
C	6.9711	3.7744	-1.9481
H	6.5969	3.0144	-2.6285
C	3.6671	4.1563	-1.448
C	2.4841	3.4168	-1.6132
H	2.3798	2.4264	-1.1692
C	1.4177	3.9446	-2.3357
H	0.5111	3.355	-2.4348
C	1.5142	5.2206	-2.9017
H	0.6792	5.6329	-3.4616
C	2.682	5.9626	-2.7324
H	2.766	6.9562	-3.1663
C	3.7559	5.4327	-2.0089
H	4.6677	6.0115	-1.8952
C	4.3092	0.2646	3.2526
H	4.3212	-0.4165	2.3968

C	5.6066	-0.0243	4.0158
C	5.9085	-1.3626	4.3237
H	5.2082	-2.1449	4.0387
C	7.0793	-1.6999	5
H	7.2876	-2.742	5.2286
C	7.9802	-0.7018	5.3837
H	8.8945	-0.9615	5.9108
C	7.6932	0.628	5.0838
H	8.3852	1.4143	5.375
C	6.5166	0.9638	4.4055
H	6.3141	2.0048	4.1755
C	3.0321	-0.0858	4.0261
C	2.9843	-0.2101	5.4178
H	3.8894	-0.0859	6.005
C	1.7765	-0.5043	6.0577
H	1.7515	-0.5935	7.1412
C	0.6102	-0.6757	5.3134
H	-0.3318	-0.8839	5.8124
C	0.6529	-0.5538	3.9213
H	-0.2504	-0.66	3.3286
C	1.8545	-0.2591	3.2849
H	1.8774	-0.1278	2.202
C	5.8858	-0.5733	-0.8455
H	6.6001	-1.1573	-1.4014
C	6.0524	0.4646	0.008
H	6.9383	0.9828	0.3388
C	3.9429	-1.9569	-1.5944
C	3.2644	-1.7767	-2.817
C	2.7266	-2.9103	-3.4338
H	2.1884	-2.7878	-4.3678
C	2.8503	-4.1891	-2.8828
C	3.5448	-4.3302	-1.6794
H	3.645	-5.316	-1.2334
C	4.101	-3.2323	-1.0153
C	2.2129	-5.3847	-3.5508
H	1.2303	-5.5965	-3.1088
H	2.8233	-6.2865	-3.4309
H	2.063	-5.213	-4.6215
C	3.1456	-0.3886	-3.4618
H	2.9017	0.3042	-2.6472
C	4.4582	0.0901	-4.0934
C	4.6139	1.4598	-4.3586
H	3.8248	2.1505	-4.0716
C	5.7556	1.9393	-4.9996
H	5.8497	3.0031	-5.2019
C	6.7724	1.0586	-5.3811
H	7.6621	1.4318	-5.8816
C	6.631	-0.303	-5.1184
H	7.412	-0.9999	-5.4128
C	5.4815	-0.7827	-4.4825
H	5.3809	-1.8477	-4.2941
C	1.9866	-0.2845	-4.4612
C	2.1565	-0.5654	-5.8245
H	3.1387	-0.8382	-6.2005
C	1.0797	-0.483	-6.7091
H	1.2314	-0.7033	-7.7632
C	-0.1832	-0.1143	-6.243
H	-1.0238	-0.0549	-6.9297
C	-0.358	0.1757	-4.8886
H	-1.3348	0.4628	-4.51
C	0.7178	0.0942	-4.0029
H	0.5608	0.3266	-2.9505
C	4.8848	-3.4251	0.2884
H	4.8384	-2.4706	0.821
C	6.3763	-3.7107	0.0606
C	7.2896	-3.3518	1.0645
H	6.9289	-2.8605	1.965
C	8.6517	-3.6078	0.9217
H	9.3389	-3.3145	1.7111
C	9.1326	-4.2301	-0.2336
H	10.195	-4.4279	-0.3484
C	8.2369	-4.5892	-1.2394
H	8.5975	-5.0706	-2.145
C	6.8702	-4.3302	-1.094
H	6.1877	-4.6076	-1.8913
C	3.8065	0.0515	-0.1293
C	4.2313	-4.4475	1.2272
C	4.6488	-5.7826	1.2988
H	5.4823	-6.1198	0.6894
C	4.019	-6.681	2.1636
H	4.3596	-7.7126	2.2064
C	2.9675	-6.2566	2.9763
H	2.4824	-6.9547	3.6534
C	2.5472	-4.9262	2.9175
H	1.7309	-4.5787	3.5444
C	3.174	-4.0318	2.0502

H	2.8325	-2.9998	2.0113

Table S6. Calculated (DFT) Cartesian Coordinates of **2**.

Cu	-1.7881	0.1445	-0.5222	C	4.4504	1.6563	-0.6624
Cu	1.6851	-0.2415	-0.679	C	4.2555	2.6994	0.2615
S	-0.1251	-0.2267	-1.7789	C	4.2538	4.0099	-0.2331
N	-4.6256	-0.02	-0.0601	C	4.4336	4.2653	-1.5882
N	-3.7528	1.6692	0.9496	C	4.6191	3.2127	-2.4818
N	3.9111	-1.6888	0.4161	H	4.7509	3.428	-3.5371
N	4.4878	0.2983	-0.1771	C	4.6346	1.8846	-2.0425
C	-3.4195	0.5721	0.2049	C	4.786	0.7302	-3.0298
C	-5.6788	0.7019	0.4989	C	5.7394	1.0426	-4.1955
H	-6.7057	0.39	0.3904	C	3.4029	0.2867	-3.5476
C	-5.1297	1.7627	1.1383	C	4.0486	2.4473	1.7513
H	-5.5771	2.5683	1.6991	C	5.227	2.9921	2.5818
C	-4.8089	-1.2471	-0.8004	C	2.7063	3.0211	2.2422
C	-5.2276	-2.3924	-0.0958	C	5.2943	-1.6111	0.5652
C	-5.4975	-3.5509	-0.8365	H	5.8783	-2.4471	0.9163
C	-5.334	-3.5733	-2.2159	C	5.6569	-0.359	0.1961
C	-4.8745	-2.4395	-2.8831	H	6.6217	0.122	0.157
H	-4.7204	-2.4828	-3.9556	C	3.1485	-2.8764	0.7268
C	-4.5986	-1.2514	-2.199	C	2.5738	-3.6264	-0.3262
C	-5.3514	-2.4324	1.4255	C	1.9229	-4.8151	0.0189
C	-4.3775	-3.4614	2.0332	C	1.842	-5.2436	1.3422
C	-6.7984	-2.703	1.8796	C	2.3876	-4.4704	2.359
C	-4.0791	-0.0167	-2.9341	H	2.2939	-4.7976	3.3908
C	-3.33	-0.3397	-4.2343	C	3.0435	-3.2645	2.0767
C	-5.2112	0.9961	-3.1948	C	2.6424	-3.1557	-1.7787
C	-2.8045	2.6391	1.4405	C	4.0216	-3.4498	-2.4002
C	-2.5657	3.7955	0.6711	C	1.5205	-3.7155	-2.6626
C	-1.6774	4.7425	1.1938	C	3.5708	-2.414	3.2305
H	-1.4676	5.6466	0.6318	C	4.6804	-3.1359	4.0189
C	-1.051	4.5401	2.4209	C	3.3913	-0.5108	-0.0522
C	-1.2859	3.3756	3.1468	C	2.4263	-1.9745	4.1648
H	-0.775	3.2246	4.0925	H	-0.3676	5.2908	2.8094
C	-2.1667	2.3962	2.6723	H	-5.826	-4.4487	-0.32
C	-2.3529	1.0777	3.4156	H	-5.5461	-4.4814	-2.7743
C	-1.2715	0.0742	2.9595	H	-4.6159	-4.4824	1.7133
C	-2.3694	1.2251	4.9457	H	-4.4314	-3.4347	3.1283
C	-3.1794	3.9695	-0.7157	H	-3.3458	-3.2482	1.7347
C	-2.2635	3.3211	-1.7757	H	-5.0622	-1.454	1.8195
C	-3.4915	5.4321	-1.073	H	-7.1489	-3.6851	1.541

H	-7.4915	-1.9513	1.4846
H	-6.8671	-2.6851	2.9739
H	-4.132	3.4281	-0.7319
H	-1.2824	3.81	-1.7976
H	-2.7112	3.4056	-2.7737
H	-2.0878	2.2604	-1.5647
H	-2.5807	6.0267	-1.2083
H	-4.1027	5.9189	-0.304
H	-4.0422	5.4711	-2.0195
H	-3.1106	1.9627	5.2745
H	-1.3932	1.5277	5.3418
H	-2.6187	0.2633	5.4083
H	-3.3239	0.6635	3.1219
H	-0.271	0.4285	3.2369
H	-1.2936	-0.0507	1.8706
H	-1.4311	-0.9064	3.4247
H	-5.9654	0.57	-3.8687
H	-4.8107	1.9001	-3.6684
H	-5.7187	1.2959	-2.2719
H	-3.3338	0.4403	-2.2732
H	-3.9931	-0.7359	-5.0138
H	-2.5186	-1.0519	-4.056
H	-2.8772	0.5777	-4.6252
H	5.3254	1.7955	-4.8759
H	5.9072	0.1355	-4.7868
H	6.7125	1.4035	-3.8426
H	5.2167	-0.118	-2.4855
H	2.9023	1.0999	-4.0855
H	2.7452	-0.0064	-2.7228
H	3.5018	-0.5668	-4.2295
H	5.3179	4.0802	2.4822
H	6.1782	2.549	2.2653
H	5.0841	2.7652	3.6451
H	4.0069	1.3655	1.9072
H	2.6745	4.1134	2.1516
H	2.5536	2.7719	3.2993
H	1.8685	2.6114	1.6691
H	1.9514	-2.8325	4.6543
H	1.6525	-1.4341	3.6098
H	2.811	-1.314	4.9514
H	4.0062	-1.5014	2.8138
H	4.3075	-4.0548	4.4862
H	5.0663	-2.4902	4.8167
H	5.5204	-3.4127	3.3714
H	4.2031	-4.5311	-2.4478
H	4.8404	-2.9985	-1.83
H	4.0677	-3.0573	-3.4228
H	2.4786	-2.0714	-1.7527
H	1.612	-4.7975	-2.823
H	1.5648	-3.2341	-3.6453
H	0.5381	-3.492	-2.2364
H	1.4637	-5.4134	-0.7599
H	1.335	-6.1753	1.5796
H	4.1052	4.8384	0.4538
H	4.4258	5.2892	-1.9529

5. References:

1. For a general description of air-sensitive techniques and equipment, see: Wayda, A. L.; Darensbourg, M. Y. *Experimental organometallic chemistry: a practicum in synthesis and characterization*; *ACS Symp. Ser.*, **357**, American Chemical Society: Washington, D.C., 1987.

2. A. Gómez-Suárez, R. S. Ramón, O. Songis, A. M. Z. Slawin, C. S. J. Cazin and S. P. Nolan, *Organometallics*, 2011, **30**, 5463–5470.

3. C. Dash, A. Das, M. Yousufuddin and H. V. R. Dias, *Inorg. Chem.*, 2013, **52**, 1584–1590.

4. V. Jurkauskas, J. P. Sadighi, S. L. Buchwald, *Org. Lett.*, 2003, **5**, 2417–2420.

5. (a) J. R. Herron, Z. T. Ball, *J. Am. Chem. Soc.*, 2008, **130**, 16486–16487. (b) Source cited in (a) as the first report of (IPr)CuF: D. S. Laitar, Ph.D. Thesis., Massachusetts Institute of Technology, Cambridge, MA, USA, 2006.

6. N. P. Mankad, D. S. Laitar and J. P. Sadighi, *Organometallics*, 2004, **23**, 3369–3371.

7. J. Zhai, M. D. Hopkins and G. L. Hillhouse, *Organometallics*, 2015, ASAP, DOI: 10.1021/acs.organomet.5b00421.

8. G. R. Fulmer, A. J. M. Miller, N. H. Sherden, H. E. Gottlieb, A. Nudelman, B. M. Stoltz, J. E. Bercaw, K. I. Goldberg, *Organometallics*, 2010, **29**, 2176–2179.

9. Assignments of ^{13}C-NMR resonances are based on previously reported assignments for (IPr)Cu(OAc). ^{13}C-NMR (C_6D_6): δ 182.6 (*C*Cu), 146.2 (*ortho*-C), 135.3 (*ipso*-C), 131.1 (*para*-C), 128.9 (*C*OOCH$_3$), 124.7 (*meta*-C), 123.1 (-N*C*H=*C*HN-), 29.4 (*C*H(CH$_3$)$_2$), 25.4 (CH(*C*H$_3$)$_2$), 24.2 (CH(*C*H$_3$)$_2$ and COO*C*H$_3$). See: N. P. Mankad, T. G. Gray, D. S. Laitar, J. P. Sadighi, *Organometallics*, 2004, **23**, 1191–1193.

10. ^{1}H-NMR resonances of IPr in THF-d_8: δ 7.35 (m, 2H, p-$C_6H_3{}^i$Pr$_2$), 7.26 (m, 4H, m-$C_6H_3{}^i$Pr$_2$), 7.17 (s, 2H, -NC*H*=C*H*N-), 2.83 (sept., 4H, -C*H*(CH$_3$)$_2$), 1.20 (d, 12H, -CH(C*H*$_3$)$_2$), 1.16 (d, 12H, -CH(C*H*$_3$)$_2$). See: O. Hollóczki, P. Terleczky, D. Szieberth, G. Mourgas, D. Gudat and L. Nyulászi, *J. Am. Chem. Soc.*, 2011, **133**, 780–789.

11. G. M. Sheldrick, *Acta Cryst.* 2015, **A71**, 3–8.

12. G. M. Sheldrick, *Acta Cryst.* 2008, **A64**, 112–122

13. The S–H bond length and M–S–H bond angle in a metal hydrosulfido complex are typically in the range of 1.0–1.4 Å and 100–113°, respectively. See: (a) A. Bauer, K. B. Capps, B. Wixmerten, K. A. Abboud and C. D. Hoff, *Inorg. Chem.*, 1999, **38**, 2136–

2142. (b) J. I. Pinkas, I. Císařová, M. Horáček, J. i. Kubišta and K. Mach, *Organometallics*, 2011, **30**, 1034–1045. (c) K. Hashizume, Y. Mizobe and M. Hidai, *Organometallics*, 1996, **15**, 3303–3309. (d) S.-i. Kabashima, S. Kuwata and M. Hidai, *J. Am. Chem. Soc.*, 1999, **121**, 7837–7845. (e) H.-C. Liang and P. A. Shapley, *Organometallics*, 1996, **15**, 1331–1333. (f) P. G. Jessop, C. L. Lee, G. Rastar, B. R. James, C. J. L. Lock and R. Faggiani, *Inorg. Chem.*, 1992, **31**, 4601–4605.

14. A. Thorn, B. Dittrich and G. M. Sheldrick, *Acta Cryst.*, 2012, **68**, 448–451.

15. Gaussian 09, Revision A.02, M. J. Frisch, G. W. Trucks, H. B. Schlegel, G. E. Scuseria, M. A. Robb, J. R. Cheeseman, G. Scalmani, V. Barone, B. Mennucci, G. A. Petersson, H. Nakatsuji, M. Caricato, X. Li, H. P. Hratchian, A. F. Izmaylov, J. Bloino, G. Zheng, J. L. Sonnenberg, M. Hada, M. Ehara, K. Toyota, R. Fukuda, J. Hasegawa, M. Ishida, T. Nakajima, Y. Honda, O. Kitao, H. Nakai, T. Vreven, J. A. Montgomery, Jr., J. E. Peralta, F. Ogliaro, M. Bearpark, J. J. Heyd, E. Brothers, K. N. Kudin, V. N. Staroverov, R. Kobayashi, J. Normand, K. Raghavachari, A. Rendell, J. C. Burant, S. S. Iyengar, J. Tomasi, M. Cossi, N. Rega, J. M. Millam, M. Klene, J. E. Knox, J. B. Cross, V. Bakken, C. Adamo, J. Jaramillo, R. Gomperts, R. E. Stratmann, O. Yazyev, A. J. Austin, R. Cammi, C. Pomelli, J. W. Ochterski, R. L. Martin, K. Morokuma, V. G. Zakrzewski, G. A. Voth, P. Salvador, J. J. Dannenberg, S. Dapprich, A. D. Daniels, O. Farkas, J. B. Foresman, J. V. Ortiz, J. Cioslowski, and D. J. Fox, Gaussian, Inc., Wallingford CT, 2009.

16. Avogadro: an open-source molecular builder and visualization tool. Version 1.0.1. http://avogadro.openmolecules.net/. M. D Hanwell, D. E. Curtis, D. C. Lonie, T. Vandermeersch, E. Zurek and G. R. Hutchison, *J. Cheminform.*, 2012, **4**:17.

17. (a) A. D. Becke, *J. Chem. Phys.*, 1993, **98**, 5648–5652. (b) C. Lee, W. Yang, R. G. Parr, *Phys. Rev. B.* 1988, **37**, 785–789.

18. (a) M. M. Francl, W. J. Pietro, W. J. Hehre, J. S. Binkley, M. S. Gordon, D. J. DeFrees, J. A. Pople, *J. Chem. Phys.*, 1982, **77**, 3654–3655. (b) P. C. Hariharan, J. A. Pople, *Theor. Chem. Acc.*, 1973, **28**, 213–222.

19. L. A. Goj, E. D. Blue, S. A. Delp, T. B. Gunnoe, T. R. Cundari, A. W. Pierpont, J. L. Petersen and P. D. Boyle, *Inorg. Chem.*, 2006, **45**, 9032–9045.
